# Amino Acid Functionalization Influences the Binding of Thiophene‐Vinylene‐Benzothiazolium‐Based Fluorescent Ligands to Protein Deposits in Tissue Sections With Alzheimer’s Disease Pathology

**DOI:** 10.1002/cmdc.70558

**Published:** 2026-11-03

**Authors:** Linnea Björk, Ruben Vidal, Bernardino Ghetti, Mikael Lindgren, Therése Klingstedt, K. Peter R. Nilsson

**Affiliations:** ^1^ Department of Physics Chemistry and Biology Linköping University Linköping Sweden; ^2^ Department of Pathology and Laboratory Medicine Indiana University School of Medicine Indianapolis Indiana USA; ^3^ Department of Physics The Norwegian University of Science and Technology, NTNU Trondheim Norway

**Keywords:** Alzheimer´s disease, amyloid β, fluorescence, ligands, tau

## Abstract

Detecting and distinguishing between structurally diverse protein aggregates involved in various neurodegenerative diseases, such as Alzheimer’s disease (AD) and Parkinson’s disease (PD), remain key challenges in the development of molecular imaging tools. Recently, bi‐thiophene‐vinyl‐benzothiazoliums (bTVBTs), a class of thiophene‐based optical ligands, were synthesized and shown to enable selective labeling of tau pathology in AD brain tissue. Building on this framework, we report here the design and evaluation of a novel series of bTVBT ligands functionalized with L‐amino acids (glycine, alanine, valine, tyrosine, or glutamate) to explore their influence on ligand–filament interactions. When applied to sporadic AD brain tissue sections, the ligands displayed preferential binding to tau deposits when using 100 nM ligand, whereas all ligands bound to both amyloid‐β (Aβ) and tau aggregates at higher concentrations. The glycine‐functionalized bTVBT scaffold demonstrated the strongest fluorescence upon binding and was labeling all types of tau inclusions present in sAD brain tissue, whereas the glutamate‐functionalized ligand was the least potent. Thus, the chemical nature of the amino acid influenced the binding mode of the ligand to protein deposits. The observed trend in the ligands’ binding mode to the protein aggregates could also be correlated with distinct solvatochromic effects, indicating that certain amino acid side chains interfere with favorable interligand π–π stacking, thereby reducing binding specificity. Collectively, these results pinpoint that minor chemical alterations of the bTVBT scaffold influence the binding mode of the ligands to protein deposits, and we foresee that our findings will aid in designing ligands targeting proteinaceous aggregated species in AD and related neurodegenerative diseases.

## Graphical abstract

Bi‐thiophene‐vinyl‐benzothiazolium (bTVBT) ligands with distinct amino acid functionalities displayed differential binding to protein aggregates in human brain tissue sections with Alzheimer's disease pathology. bTVBT‐G‐OMe showed staining of GT‐38‐positive tau aggregates, whereas staining was weaker for bTVBT‐E‐diOMe. The binding mode of the ligands to protein aggregates could be correlated to distinct solvatochromic effects.

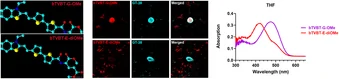

## 1 Introduction

Neurodegenerative diseases, such as Alzheimer’s disease (AD) and Parkinson’s disease (PD), are defined by the accumulation and progressive spread of misfolded protein aggregates within the brain. These pathological deposits—extracellular plaques composed of the amyloid‐β (Aβ) peptide and intracellular neurofibrillary tangles (NFTs) formed by the tau protein in AD and intracellular Lewy bodies comprised of the α‐synuclein protein in PD—share a characteristic cross β‐sheet fibrillar structure [1]. Over the years, a variety of ligands have been developed to recognize these structures, including fluorescent dyes and positron emission tomography tracers designed for in vivo imaging [2]. Despite these advances, the structural diversity among aggregates of the same protein has emerged as a major challenge for accurate detection and characterization [3].

Recent structural studies, particularly those employing cryo‐electron microscopy (cryo‐EM), have highlighted the polymorphic nature of Aβ and tau aggregates [4, 5, 6, 7, 8, 9, 10, 11, 12], both between and within individuals, potentially underlying the clinical and pathological heterogeneity observed in AD and related disorders [13, 14]. Such heterogeneity necessitates the development of molecular tools capable of distinguishing between aggregate variants rather than simply detecting their presence. In this context, thiophene‐based ligands, especially luminescent conjugated oligothiophenes (LCOs), have proven to be versatile tools for fluorescence imaging of protein aggregates [2]. Their conformational sensitivity and tunable optical properties allow not only for visualization but also for spectral discrimination of different types of aggregates [15, 16, 17, 18]. This feature is particularly advantageous when studying disease‐associated variations [15, 19, 20, 21] or age‐dependent changes in protein aggregation [22, 23]. Building upon these foundations, more recent ligands, such as bi‐thiophene‐vinyl‐benzothiazoliums (bTVBTs) and other thiophene‐vinylene‐based (TV) ligands, have been developed to preferentially bind specific aggregated species of Aβ or tau [24, 25, 26, 27]. The binding selectivity of these ligands can be governed by including certain heterocyclic moieties in the ligand structure [26]. For example, the tau selectivity exhibited by bTVBTs in sAD brain tissue sections was abolished when replacing the terminal thiophene unit with heterocyclic groups such as 1,3‐benzodioxole or 1,2‐dimethoxybenzene, resulting in ligands binding to deposits composed of tau as well as of Aβ [26]. Furthermore, when substituting the benzothiazole moiety in TV ligand HS‐335 with benzothiazolium, the selectivity of the resulting ligand HS‐332 changed from Aβ to tau on sAD brain tissue sections [26]. To study the interactions between the bTVBT scaffold and the tau filament in more detail, unbiased molecular dynamics (MD) simulations have been performed. By employing ligand bTVBT4 and the sAD tau filament structure previously determined by cryo‐EM [9], the major binding site was found in a hydrophobic pocket defined by amino acid residues isoleucine (I) 360, threonine (T) 361, and histidine (H) 362 [28]. Unbiased MD simulations also identified the same binding site for tau ligand HS‐332 [26].

In 2023, Tao et al. used cryo‐EM to examine the binding mechanisms of various small organic compounds, including classic dyes, clinical imaging tracers, and newly identified binders, to α‐syn filaments formed from recombinant protein [29]. The study revealed that the small molecules bound to the filaments through mechanisms that differed significantly from traditional ligand–protein interactions. Instead of the well‐defined ligand‐binding pockets found on native proteins, the binding sites for the ligands on amyloid filaments are shaped like grooves on the common cross‐β‐sheet structure and are therefore only restricted in two dimensions. To achieve increased specificity of the ligand–filament interaction, the results reported by Tao et al. showed the importance of established π–π interactions between the ligands upon binding [29]. The most favorable ligand orientation to achieve interligand π–π stacking was in a diagonal binding pattern, as this shortened the distance between the aromatic rings. This enabled parallel π–π interactions and thereby strong interligand connections that resulted in increased specificity of the ligand when binding to the filament. Ligands were also observed in horizontal or vertical binding modes in which the former provided some interligand connections but not as strong as for the diagonal orientation [29].

Chemical diversity of proteins is achieved due to a library of amino acids with different side‐chain characteristics, and the addition of different combinations of amino acids to the chemical structure of the pentameric LCO HS‐84 had a great impact on the ligand’s ability to recognize certain types of Aβ and tau deposits [30]. Herein, we employ a similar concept to generate a novel class of bTVBT‐based ligands functionalized with different L‐amino acids with the aim of pinpointing more details regarding the binding modes of bTVBT ligands to protein aggregates in sporadic AD (sAD) brain tissue sections. The amino acids used to functionalize the bTVBT scaffold included glycine (G), alanine (A), valine (V), tyrosine (Y), and glutamic adic (E). These amino acids were chosen to yield bTVBT ligands with minor chemical differences in hydrophobic, polar, and steric properties. When applied to brain tissue sections from three individuals with sAD using 100 nM ligand, the novel ligands showed selective binding toward tau deposits. At higher concentrations (10 μM), all ligands also bound to Aβ aggregates. However, at this higher concentration, the ligand spectral signatures from Aβ deposits were different from the signatures observed from tau pathologies, indicating that the ligands were binding differently to these protein aggregates. The binding mode toward tau aggregates was also modulated depending on the amino acid functionalization of the bTVBT scaffold, suggesting that the amino acid substitutions affected the ligand–filament interactions. In addition, the ligands exhibiting the most pronounced emission intensity when bound to tau deposits also displayed distinct solvatochromic behavior, signifying that the binding of the bTVBT scaffold to tau filaments in the sAD brain tissue might be dependent on interligand interactions through efficient stacking of the ligand. Overall, we anticipate that our findings will assist in designing ligands targeting distinct aggregated pathologies associated with neurodegenerative diseases, such as AD.

## 2 Results and Discussion

### 2.1 Synthesis and Optical Characterization of Amino Acid Functionalized Bi‐Thiophene‐Vinylene‐Benzothiazolium (bTVBT) Based Ligands

As the starting scaffold, we chose the previously reported tau‐selective ligand bTVBT2, as it has a synthetically accessible carboxylic acid side chain group suitable for coupling to amino acids [25]. To yield amino acid functionalized bTVBTs with distinct chemical characteristics, such as hydrophobicity, polarity, and size, a set of amino acids with different side chain properties were chosen; L‐glycine (G), L‐alanine (A), L‐valine (V), L‐glutamic acid (E), and L‐tyrosine (Y). As each amino acid residue was protected with methyl esters, the charge of the carboxylic acids was abolished. The synthesis started with hexafluorophosphate azabenzotriazole tetramethyl uronium (HATU)‐mediated amide coupling of the methyl ester‐protected amino acids to 5‐bromothiophene‐2‐carboxylic acid in DMF, affording amino acid functionalized monomer 1, which was subsequently coupled to (5‐formylthiophene‐2‐yl)boronic acid through a Suzuki–Miyaura cross‐coupling reaction. The formed thiophene dimer 2 was then reacted through a condensation reaction with 3‐ethyl‐2‐methylbenzothiazolium iodide using pyridine as a base in dry methanol, affording the final product bTVBT‐aa‐OMe (Scheme 1). In total, five different ligands denoted bTVBT‐G‐OMe, bTVBT‐A‐OMe, bTVBT‐V‐OMe, bTVBT‐Y‐OMe, and bTVBT‐E‐diOMe (Figure 1) were generated by this synthetic procedure.

**SCHEME 1:**
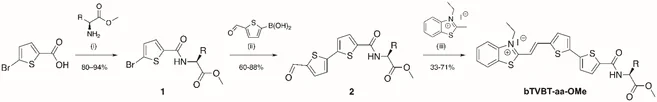
Synthesis of amino acid functionalized bTVBTs. General conditions and reagents: (i) DMF, DIPEA, HATU; (ii) PEPPSI‐IPr, K_2_CO_3_, 1,4‐dioxane:MeOH (4:1), 80 °C; and (iii) MeOH, pyridine, 75 °C.

**FIGURE 1:**
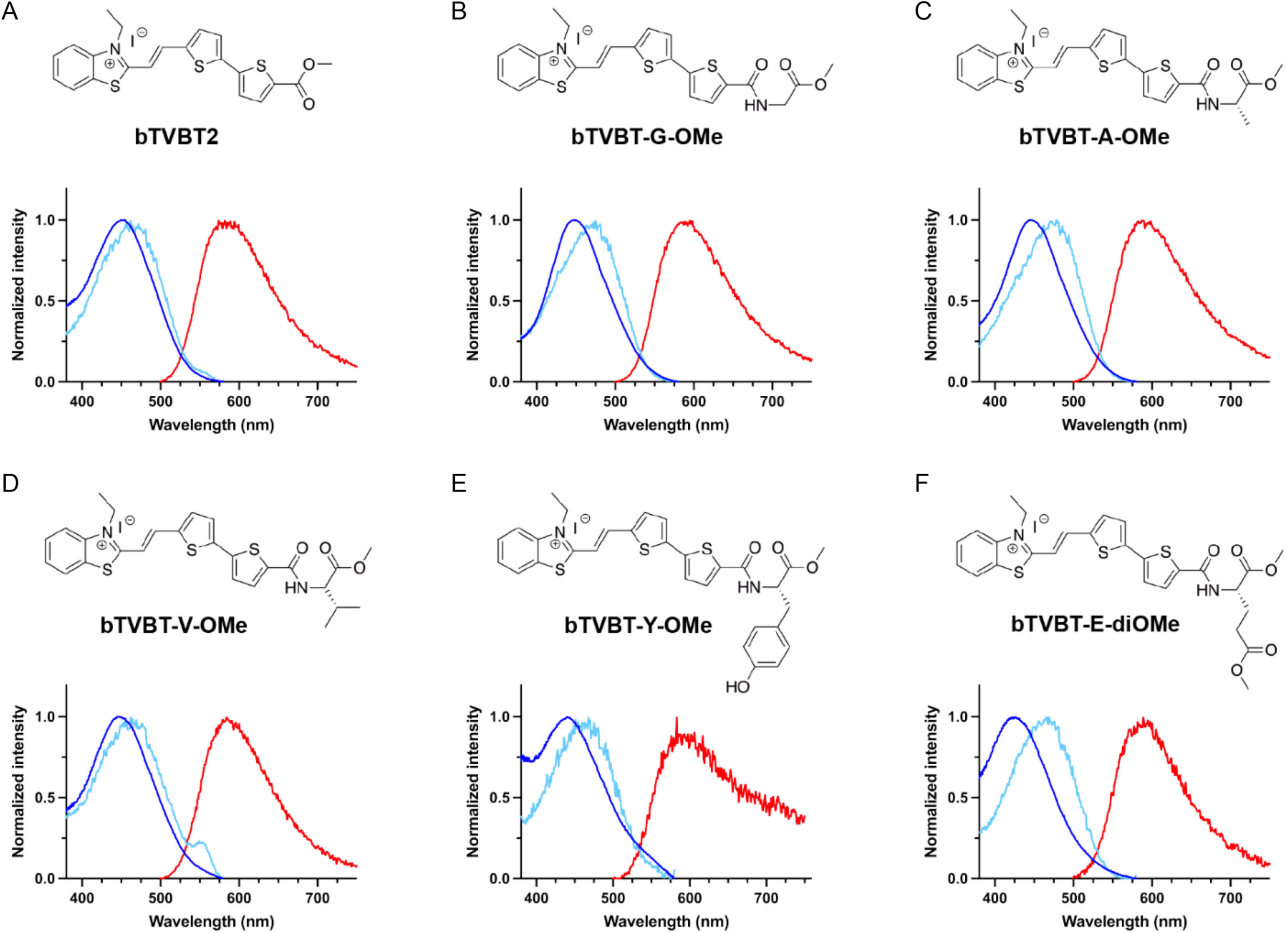
Chemical structure and photophysical properties of bTVBT2 (A) and the amino acid functionalized bTVBTs bTVBT‐G‐OMe (B), bTVBT‐A‐OMe (C), bTVBT‐V‐OMe (D), bTVBT‐Y‐OMe (E), and bTVBT‐E‐diOMe (F). For the optical characterization, the ligands were diluted to 30 μM in PBS. The absorption (dark blue line), excitation (light blue line) and emission (red line) spectra of the amino acid functionalized ligands resembled the spectral characteristics of bTVBT2 (A).

Stock solutions (1.5 mM) were prepared by dissolving each ligand in dimethyl sulfoxide (DMSO). For the optical characterization, the ligands were further diluted in phosphate‐buffered saline (10 mM phosphate, 140 mM NaCl, 2.7 mM KCl, pH 7.4, PBS) to 30 μM. The amino acid functionalized bTVBTs displayed absorption maxima between 427 and 447 nm, and the maxima were similar to the absorption maximum displayed by bTVBT2 (450 nm) (Figure 1). The emission characteristics of the functionalized bTVBTs were also comparable to those displayed by bTVBT2, with the latter ligand exhibiting an emission maximum at 582 nm (Figure 1A) and the former ligands demonstrating an emission maximum between 583 and 595 nm (Figure 1B–F). Furthermore, the amino acid functionalized bTVBTs showed excitation maxima in PBS between 462 and 478 nm. The excitation maximum of bTVBT2 was 462 nm. All amino acid functionalized bTVBTs displayed a similar quantum yield, approximately 0.3% (Table S1 and Figure S2), and these values were slightly lower than the quantum yield (0.61%) obtained for bTVBT2 diluted in PBS. In pure DMSO or diluted in THF, the quantum yields were slightly higher for all the ligands and comparable to those of bTVBT2 (Table S1). Hence, the optical characterization of the ligands showed that the amino acid functionalization of the bTVBT scaffold did not result in any major alterations of the photophysical properties as compared with the characteristics of earlier reported bTVBTs, such as bTVBT2 [25, 28].

### 2.2 Ligand Binding to Protein Deposits in AD Brain Tissue Sections

In 2017, we introduced the bTVBT scaffold for selective labeling of filamentous tau inclusions in sAD brain tissue [25]. By using unbiased MD simulations, the binding of ligand bTVBT4 (Figure S1) to the sAD tau filament structure was shown to occur at a hydrophobic pocket defined by amino acids I360, T361, and H362 [28], and the same binding site was also suggested for tau ligand HS‐332 [26]. Hence, the amino acid functionalized bTVBT‐based ligands are most likely to have the same binding mode, but the chemical nature of the amino acid functionality might influence the ligand–filament interaction, which would possibly shed light on the binding mechanism of the ligands. To explore the binding properties of the amino acid functionalized bTVBT ligands to Aβ and tau deposits, they were applied to frozen brain tissue sections from the parietal or frontal cortex of three individuals diagnosed with sAD (Table S2). The previously reported ligand bTVBT2 [25], sharing the bi‐thiophene‐vinylene‐benzothiazolium moiety as well as the methyl ester side chain (Figure 1A) with the novel ligands, was included as a positive control for labeling of tau filamentous inclusions, which are herein defined as structures positive with GT‐38 anti‐tau antibody. In case 1, when used at 100 nM, all included ligands showed binding to the different types of tau deposits present in sAD, that is NFTs, dystrophic neurites (DNs), and neuropil threads (NTs). However, in comparison with bTVBT2, which demonstrated strong fluorescence from all inclusion types and an apparent complete colocalization with the GT‐38 antibody (Figures 2A, S3, and S4), the amino acid‐functionalized bTVBT ligands showed a variation in fluorescence intensity upon binding, as well as different levels of colocalization with GT‐38. bTVBT‐G‐OMe and bTVBT‐A‐OMe demonstrated similar binding patterns and spectral properties to those of bTVBT2 on tau filamentous inclusions (Figures 2B,C, S3, and S4). Thus, similar to what has been reported for other bTVBTs [25, 26, 28], these two ligands showed an environment‐dependent spectral shift associated with a distinct conformation of the ligand, as well as an enhanced fluorescence intensity, when binding to the tau aggregates. However, compared with bTVBT2, they displayed a lower fluorescence intensity upon binding, and they also occasionally failed to label some immunopositive tau deposits, mainly DNs and NTs. The other three ligands, bTVBT‐V‐OMe, bTVBT‐Y‐OMe, and bTVBT‐E‐diOMe, showed much weaker fluorescence than bTVBT‐G‐OMe and bTVBT‐A‐OMe when binding to tau deposits (Figures 2D–F, S3, and S4). All of them demonstrated labeling of GT‐38 positive NFTs, DNs, and NTs but showed less staining, especially bTVBT‐E‐diOMe, which often failed to label NTs. Due to the weak fluorescence intensity, it was not possible to collect the emission spectra for these ligands when binding to tau. The tau staining in the two additional AD cases, cases 2 and 3, resembled the results obtained for case 1, as the most profound fluorescence was observed from aggregated tau pathologies stained by bTVBT‐G‐OMe and bTVBT‐A‐OMe (Figures S5–S10).

**FIGURE 2:**
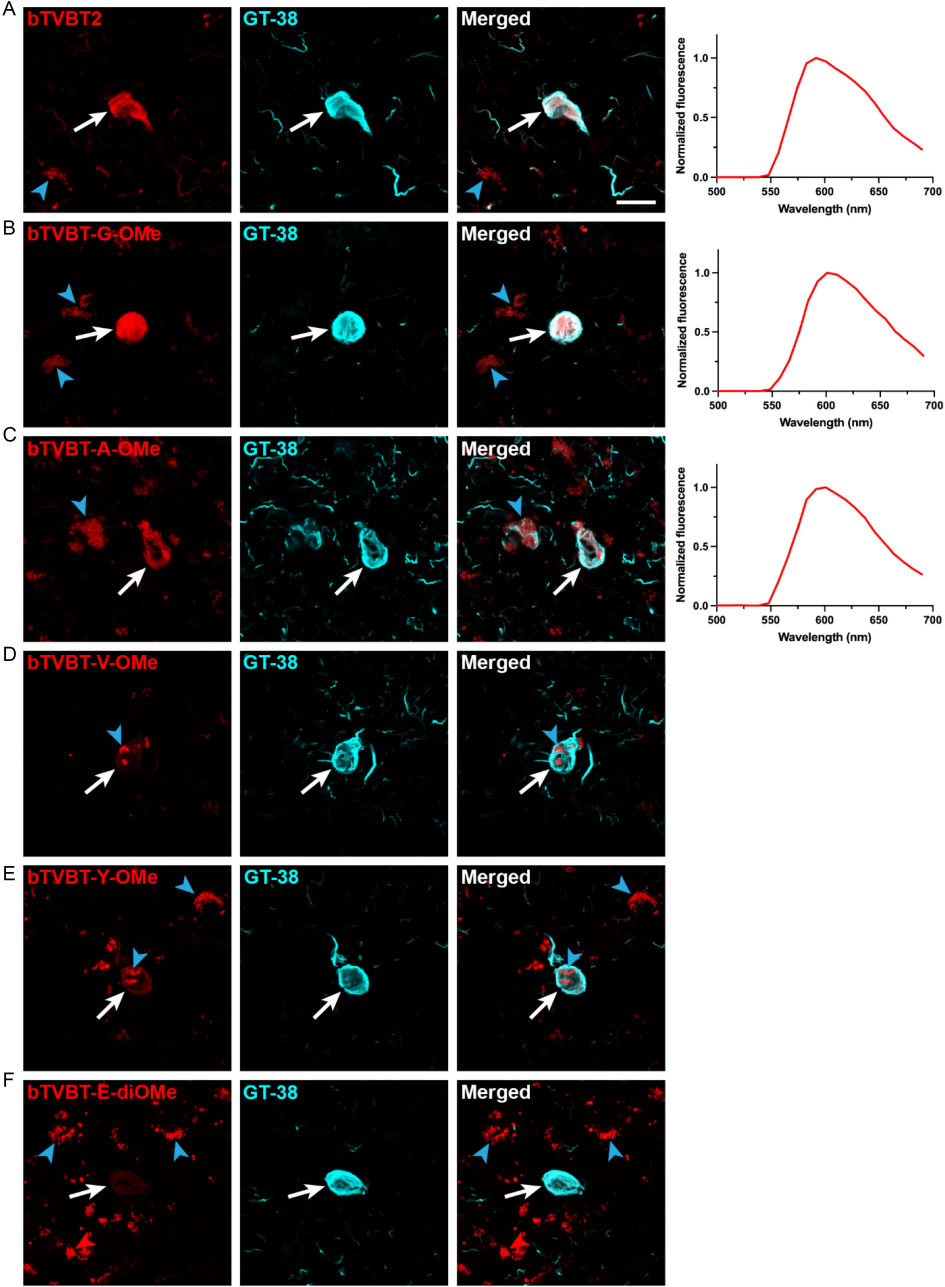
Fluorescence images of sAD brain tissue section (case 1) stained with 100 nM bTVBT2 (A) or 100 nM amino acid functionalized bTVBT ligands bTVBT‐G‐OMe (B), bTVBT‐A‐OMe (C), bTVBT‐V‐OMe (D), bTVBT‐Y‐OMe (E), or bTVBT‐E‐diOMe (F), all in red, together with GT‐38 anti‐tau antibody in cyan. bTVBT2, bTVBT‐G‐OMe, and bTVBT‐A‐OMe show strong staining of NFTs (white arrow). Autofluorescence from lipofuscin (blue arrowhead) can also be seen in the channel used to collect the fluorescence from the ligands, but the granular appearance makes it possible to distinguish lipofuscin from NFTs. The graphs to the right show the emission spectrum of bTVBT2 (A), bTVBT‐G‐OMe (B) and bTVBT‐A‐OMe (C) when binding to NFTs. Scale bar, 20 μm.

The staining result from using 100 nM ligand also revealed that all amino acid‐functionalized ligands, except bTVBT‐E‐diOMe, showed some labeling of Aβ plaques. However, the staining was limited as the number of stained Aβ plaques was low, and the ligand fluorescence was much weaker than observed for tau. The limited staining of Aβ aggregates for all ligands was also verified with the previously reported [31] Aβ selective ligand HS‐276 (Figure S1). Clearly, aggregated Aβ pathology only displayed the characteristic emission characteristics from HS‐276, whereas fluorescence from the bTVBT‐based ligands was observed from NFTs and other tau aggregates (Figure S11). Overall, the staining experiment with 100 nM ligand revealed that the amino acid‐functionalized bTVBT ligands were rather selective for tau aggregates, suggesting that the ligands have a similar selectivity toward tau deposits as bTVBT2 when used at this concentration for staining (Table 1). In addition, the ligands' binding properties toward tau aggregates were influenced by the chemical nature of the amino acid side chain substitution since the bTVBT scaffold functionalized with glycine or alanine showed binding patterns more similar to those of bTVBT2 and the anti‐tau antibody than those functionalized with the other included amino acids. From a chemical perspective, glycine and alanine have smaller R groups attached to the alpha carbon compared to valine, tyrosine, and glutamic acid, suggesting that steric effects exerted by the amino acid functionalization might influence the binding properties of the ligand.

**TABLE 1:** Qualitative assessment of the fluorescence observed from tau aggregates or Aβ deposits for the respective ligand when using 100 nM or 10 μM ligand for staining of human sAD brain tissues.

Ligand	100 nM ligand		10 μM ligand	
	Tau aggregates	Aβ deposits	Tau aggregates	Aβ deposits
bTVBT‐G‐OMe	+++	(−)^a^	+++	+++
bTVBT‐A‐OMe	+++	(−)^a^	+++	+++
bTVBT‐V‐OMe	++	(−)^a^	+++	+++
bTVBT‐E‐diOMe	++	(−)^a^	+++	+++
bTVBT‐Y‐OMe	++	+	+++	+++

a Staining was only observed from a few aggregates.

To investigate the binding mode of the amino acid functionalized bTVBTs further, we next performed staining of brain tissue sections using a higher concentration, 10 μM, of the ligands. As shown in Figure 3, at 10 μM concentration, all ligands displayed binding to both Aβ and tau aggregates. An increased fluorescence from some cell bodies as well as the background tissue was also observed. The latter was most severe for bTVBT‐V‐OMe, most likely due to the hydrophobic nature of the amino acid functionalization, making spectral imaging of this tissue sample not feasible. However, for all other ligands, spectral imaging revealed a redshift when they were bound to tau aggregates compared with when bound to Aβ deposits (Figure 3). Thus, at a higher concentration, the selectivity for tau was abolished (Table 1). As a selectivity toward aggregated tau pathologies was afforded for the ligands at lower staining concentrations (Figures 2 and S3–S11), the amino acid‐functionalized bTVBT ligands presumably have a higher affinity for tau aggregates than for Aβ deposits. In addition, the spectral difference observed for the respective ligand bound to Aβ deposits or tau aggregates signifies that the ligand binds in a slightly different fashion to these two types of protein aggregates. A similar spectral difference between Aβ deposits and tau aggregates was previously reported for thiophene‐vinyl‐benzothiazolium (TVBT) ligands having distinct heterocyclic motifs attached to the TVBT scaffold [26]. Furthermore, the selectivity for tau aggregates was also more pronounced for TVBT‐based ligands with distinct terminal heterocyclic moieties [26].

**FIGURE 3:**
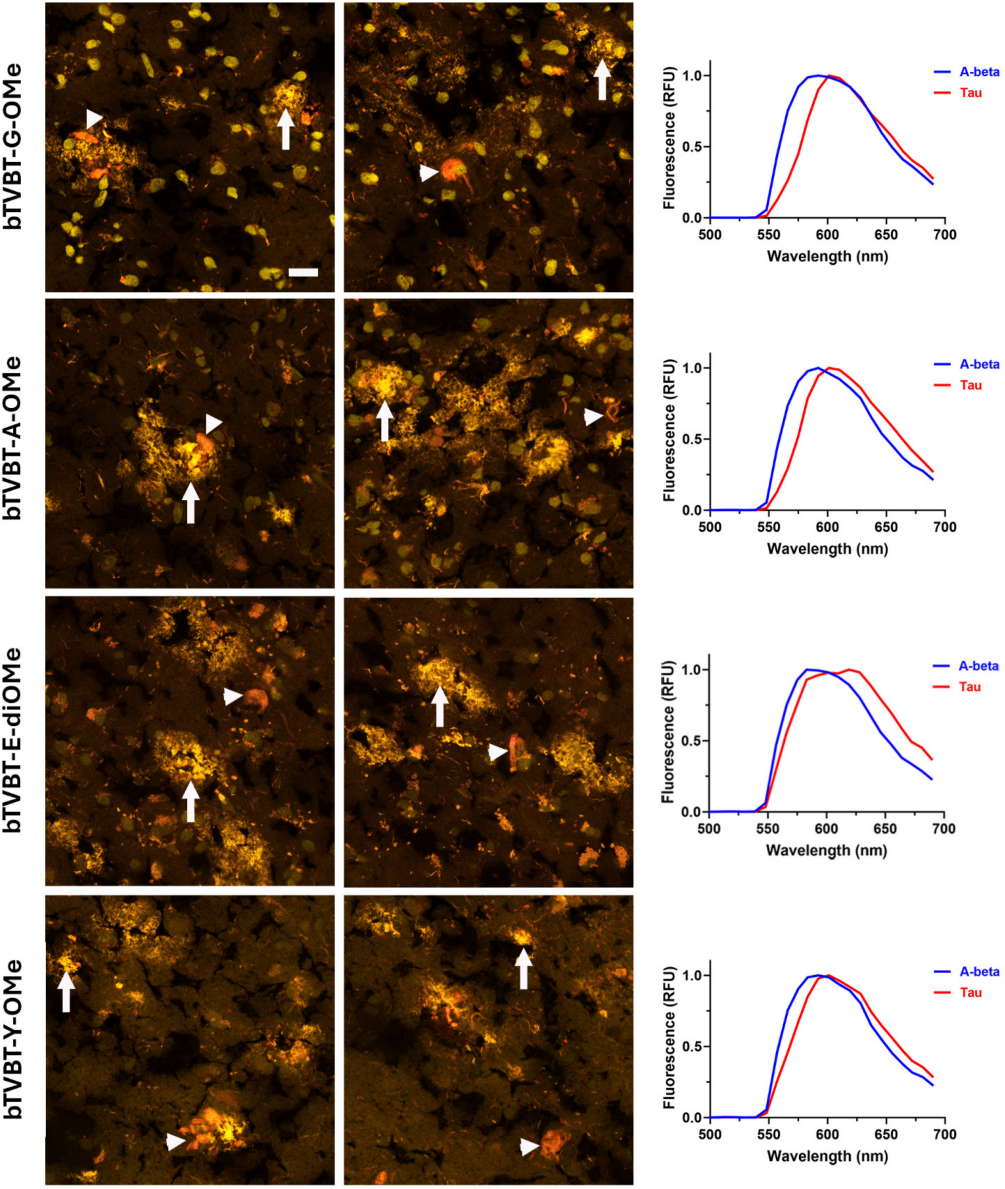
Representative fluorescence spectral images from two different areas of an sAD brain tissue section (case 1) stained with 10 μM of the respective amino acid functionalized bTVBT ligand bTVBT‐G‐OMe, bTVBT‐A‐OMe, bTVBT‐E‐diOMe or bTVBT‐Y‐OMe. All ligands show strong staining of tau aggregates (white arrowheads) and Aβ deposits (white arrows). The graphs to the right show the emission spectrum of the ligands bound to Aβ or tau aggregates, respectively. Scale bar, 20 μm.

### 2.3 Ligand Displacement With HS‐339

To further assess the influence of the amino acid functionalization on the binding mode of the bTVBT ligands to protein deposits, displacement experiments with a previously reported ligand, HS‐339 [26], which stains both aggregated Aβ and tau species, were performed. Human sAD tissue sections were prestained with 10 μM of the respective amino acid‐functionalized ligand followed by staining with 100 nM HS‐339 and analyzed with spectral imaging, as well as a channel mode setup detecting the characteristic emission from the bTVBT ligands or HS‐339 (Figure 4). For the tissue section prestained with bTVBT‐G‐OMe, both Aβ and tau aggregates displayed characteristic bTVBT‐G‐OMe emission, whereas weak fluorescence corresponding to HS‐339 was observed from a minority of Aβ deposits and some NFTs (Figure 4A). In contrast, in the section prestained with bTVBT‐A‐OMe, the majority of Aβ deposits displayed HS‐339 emission, whereas bTVBT‐A‐OMe fluorescence was only observed from some Aβ aggregates, as well as from aggregated tau pathologies, including NFTs (Figure 4B). Some of the latter was also stained by HS‐339. In the sections prestained with bTVBT‐E‐diOMe or bTVBT‐Y‐OMe, the Aβ aggregates only displayed HS‐339 emission, whereas colabeling of HS‐339 and the respective amino acid functionalized ligand was observed for some NFTs, and dominant bTVBT‐E‐diOMe or bTVBT‐Y‐OMe fluorescence was detected from other tau aggregate types (Figures 4C and S12). In contrast to bTVBT‐A‐OMe, other aggregated tau pathologies, such as DNs displaying HS‐339 fluorescence, could also be observed. Thus, a similar trend to that observed for the ligands’ ability to stain tau aggregates at 100 nM ligand (Figures 2 and S3–S10) was observed for the displacement studies with HS‐339 since it was harder to displace the ligands functionalized with glycine and alanine. In addition, it was easier to displace ligands bound to Aβ deposits compared with ligands bound to tau aggregates, suggesting that the bTVBT‐based ligands have a higher affinity for tau aggregates than for Aβ deposits.

**FIGURE 4:**
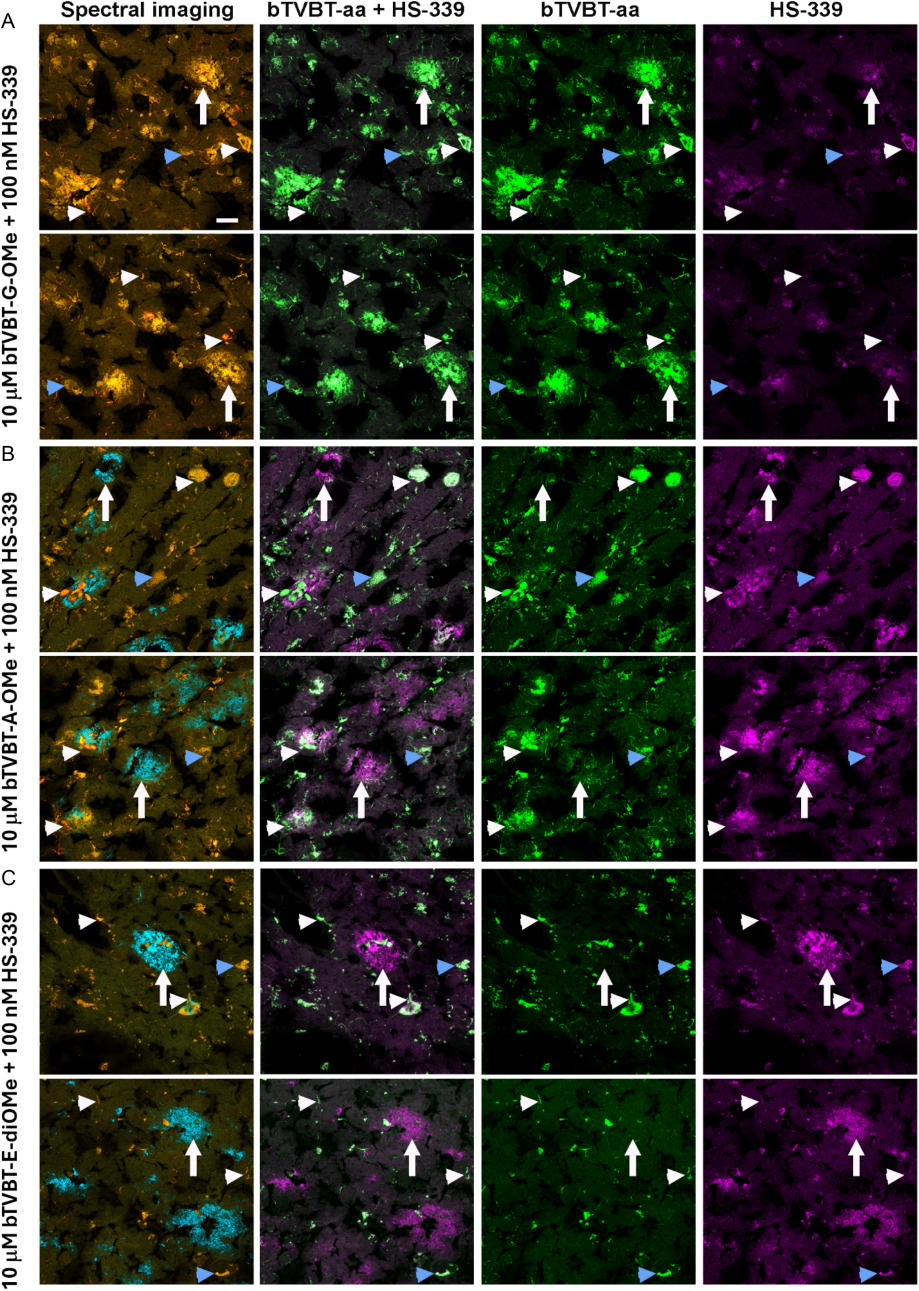
Fluorescence images of sAD brain tissue section prestained with 10 μM of the respective amino acid functionalized bTVBT ligand bTVBT‐G‐OMe (A), bTVBT‐A‐OMe (B) or bTVBT‐E‐diOMe (C) followed by 100 nM HS‐339. In the left panel, which shows the results of spectral imaging, HS‐339 labeled aggregated pathologies are seen in blue (Aβ) or blue/green (tau) whereas protein deposits labeled by amino‐acid‐functionalized bTVBT ligands display yellow/orange emission. In the channel mode images, HS‐339 labeled aggregated pathologies are seen in magenta whereas protein deposits labeled by amino‐acid‐functionalized bTVBT ligands are displayed in green. Representative Aβ deposits or tau aggregates are indicated with white arrows or white arrowheads, respectively. Autofluorescence from lipofuscin is indicated with blue arrowheads. Scale bar, 20 μm.

### 2.4 Ligand Solvatochromism

Cryo‐EM studies of several amyloid ligands bound to alpha‐synuclein (α‐synuclein) fibrils have suggested that the orientation of the bound ligands relative to the fibril axis might be vertical, horizontal, or diagonal [29]. For the two latter orientations, different forms of interligand π–π stacking modes, as well as a higher ligand specificity, were also suggested. Earlier studies have also shown that the binding mode of a ligand to a protein aggregate and its resulting spectral signature, as well as the ligand π–π stacking interactions, can be assessed by the solvatochromic behavior of the ligand in different solvents [22, 32, 33]. In this regard, the photophysical characteristics of the bTVBT ligands functionalized with amino acids dissolved in different solvents were explored. All ligands were dissolved in DMSO (1.5 mM ligand) and further diluted to a final concentration of 15 μM in methanol (MeOH), ethanol (EtOH), DMSO, acetonitrile (ACN), or tetrahydrofuran (THF). When comparing the absorption and emission characteristics of the ligands in different solvents, certain trends were observed (Table 2 and Figure 5A,B). First, bTVBT‐G‐OMe, bTVBT‐A‐OMe, and bTVBT‐V‐OMe showed different absorption characteristics in all solvents compared to bTVBT‐E‐diOMe and bTVBT‐Y‐OMe. For instance, in THF, the first three ligands displayed an absorption maximum at 475 nm (bTVBT‐G‐OMe) and 473 nm (bTVBT‐A‐OMe and bTVBT‐V‐OMe), whereas bTVBT‐E‐diOMe and bTVBT‐Y‐OMe both showed an absorption maximum at 420 nm and a shoulder at longer wavelengths (Figure 5A,B). A similar trend with a more redshifted absorption maximum for bTVBT‐G‐OMe, bTVBT‐A‐OMe, and bTVBT‐V‐OMe compared with the other ligands was also observed in MeOH, EtOH, DMSO, and ACN. This indicates that bTVBT‐G‐OMe, bTVBT‐A‐OMe, and bTVBT‐V‐OMe can adopt a distinct conformational state rendering unique optical signatures that cannot be induced in bTVBT‐E‐diOMe and bTVBT‐Y‐OMe under the same conditions. Since redshifted absorption can be a consequence of intermolecular π–π stacking in different arrangements [34, 35], it is plausible to suggest that, due to the steric effects exerted by the amino acids E and Y, bTVBT‐E‐diOMe and bTVBT‐Y‐OMe have a less tendency to form intermolecular π–π stacking in a similar fashion to bTVBT‐G‐OMe, bTVBT‐A‐OMe, and bTVBT‐V‐OMe. Second, this statement is also supported by the emission characteristics of the ligands in different solvents, as bTVBT‐G‐OMe, bTVBT‐A‐OMe, and bTVBT‐V‐OMe showed redshifted emission spectra with a maximum around 600 nm in all solvents, whereas bTVBT‐E‐diOMe and bTVBT‐Y‐OMe showed different emission profiles with less redshifted maxima and significantly higher emission intensities at shorter wavelengths (Figure 5C). In fact, in all solvents, distinct shoulders with an emission between 500 and 540 nm can be observed for bTVBT‐E‐diOMe and bTVBT‐Y‐OMe. In previous studies of thiophene‐based ligands, the emission at longer wavelengths has shown to be associated with an interchain stacking phenomenon, whereas the emission at shorter wavelengths has been assigned to an intrachain event [16, 22, 33]. Thus, since the emission at longer wavelengths, with an emission maximum around 600, was the dominant fluorescence signature for bTVBT‐G‐OMe, bTVBT‐A‐OMe, and bTVBT‐V‐OMe in all of the explored solvents, these ligands most likely have a higher tendency to form intermolecular π–π stacking compared to bTVBT‐E‐diOMe and bTVBT‐Y‐OMe. As the former three ligands also showed more pronounced staining of tau aggregates in tissue sections (Figures 2 and S3–S10), it is plausible that interligand π–π stacking is involved in the binding mode of bTVBT ligands to tau deposits. Furthermore, the concept of bTVBTs binding to tau in a nonmonomeric form was previously introduced by Shirani et al. following observations of a pronounced redshift in the ligands’ excitation maxima when binding to NFTs compared with the maxima obtained in PBS only [25]. The correlation between minor chemical alterations of the ligands and less tendency of ligands to form interligand π–π stacking when bound to protein aggregates has also been shown for anionic tetrameric oligothiophenes [22]. The ligand HS‐68 gave distinct spectral signatures when bound to different age‐related tau aggregates, whereas structural analogs of HS‐68, which had different patterns of anionic groups along the thiophene backbone, lacked the ability to distinguish tau deposits. Interestingly, similar to our observations herein, HS‐68 also displayed a unique solvatochromic behavior, associated with interligand π–π stacking, compared with the other tetrameric analogs [22].

**TABLE 2:** Absorption maximum of the bTVBT‐based ligands in different solvents.

Ligand	Abs_max_ MeOH	Abs_max_ EtOH	Abs_max_ DMSO	Abs_max_ ACN	Abs_max_ THF
bTVBT‐G‐OMe	447	456	471	453	475
bTVBT‐A‐OMe	451	460	463	452	473
bTVBT‐V‐OMe	450	458	469	454	473
bTVBT‐E‐diOMe	413	415	426	415	420
bTVBT‐Y‐OMe	407	417	434	407	420

**FIGURE 5:**
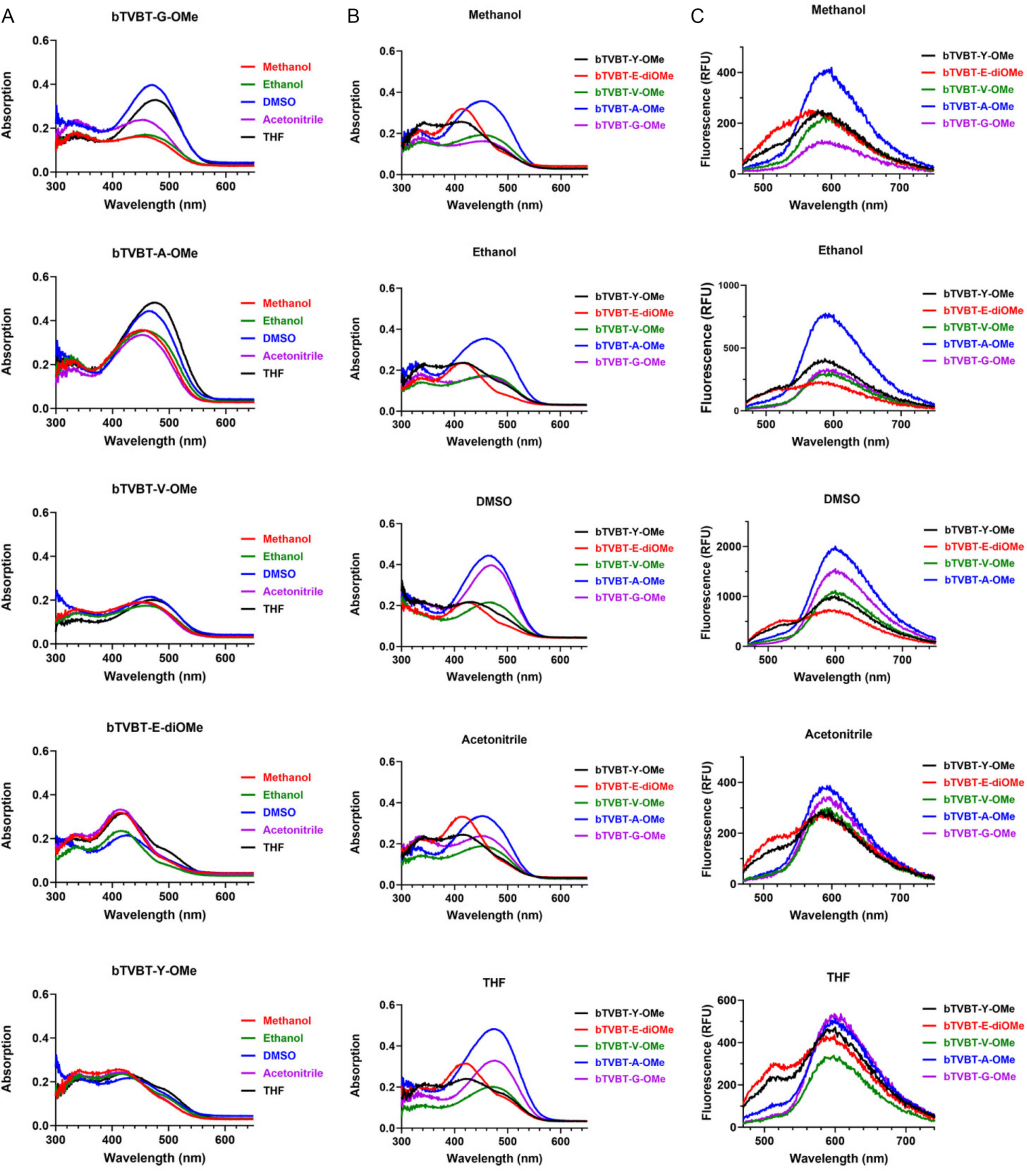
Absorption‐ and emission characteristics of the amino acid functionalized bTVBT‐based ligands in different solvents. (A) Absorption spectrum of 15 μM of the respective ligand in different solvents. (B) Comparison of the absorption spectrum for the respective ligand in different solvents. (C) Comparison of the emission spectrum of the respective ligand in different solvents. For excitation, a wavelength corresponding to the absorption maximum of the respective ligand in the different solvents was used.

Similar to structurally related ligands, interligand π–π stacking might influence the binding mode of bTVBT ligands to tau deposits. In 2021, Shi et al. reported the high‐resolution structure of ligand APN‐1607 binding to tau filaments extracted from the brain tissue of an individual with sAD [36]. The structure of APN‐1607 (Figure S1) resembles the bTVBT scaffold as it contains a benzothiazole moiety and a (di‐)vinylene bridge. Shi et al. identified two major binding sites for APN‐1607 on the tau (paired helical) filament, and one of these sites corresponded to a minor binding site shown for bTVBT4 (arginine (R) 349 and glutamine (Q) 341) by nonbiased MD simulations [28]. Interestingly, the result suggested that APN‐1607 bound parallel to the long helical axis of the filament and not in a diagonal pattern [36]. The bTVBT scaffold also shares some chemical similarities with amyloid ligands Thioflavin T and BF227a (Figure S1), with the former having a charged benzothiazolium moiety and the latter a vinylene bridge. These ligands were shown to bind to cryo‐EM‐derived structures of α‐synuclein fibrils with specific interligand interactions. BF227a was found to bind to the fibrils in a diagonal conformation that resulted in robust interligand π–π stacking and thus enhanced the ligand–protein interaction. ThT was found to bind in a slightly less efficient, horizontal pattern, where the ligand’s aromatic rings were rotated to achieve interligand π–π interactions [29]. It is hard to predict whether the bTVBTs bind in a diagonal or horizontal manner similar to APN‐1607, BF227a, or ThT, or in an entirely different way, and further experiments using cryo‐EM are necessary to resolve this matter. Such experiments are also necessary to determine the difference between the binding modes, as well as the observed difference in affinity, of bTVBT ligands for Aβ deposits versus tau aggregates. As suggested by Tao et al. [29], different forms of interligand π–π stacking modes might yield higher ligand specificity. Thus, the ability of the bTVBT‐based ligands to selectively stain tau deposits might be afforded by a distinct interligand π–π stacking mode. In fact, replacing the terminal thiophene moiety of the bTVBT scaffold with other heterocyclic moieties had a major impact on the selectivity of these ligands for Aβ or tau pathology in AD [26], indicating that the interligand π–π stacking mode might be an important chemical determinant to achieve selectivity toward distinct protein aggregates.

## 3 Conclusion

In conclusion, the functionalization of the bTVBT scaffold with different amino acids resulted in ligands exhibiting distinct binding properties for protein deposits in sAD brain tissue sections. The observations from staining of human AD brain tissue sections supported that both the affinity and selectivity toward tau deposits were influenced by the size of the amino acid. Furthermore, photophysical measurements suggested that the binding mode of the ligands might be influenced by the ligands’ ability to form interligand π–π interactions. Thus, as observed for structurally related ligands, interligand π–π interaction might be a central chemical determinant for achieving bTVBT‐based ligands displaying a high specificity toward distinct protein deposits.

## Funding

This work was supported by National Institutes of Health (Grants 2RF1NS110437‐06, P30‐AG010133, and P30‐AG072976), Vetenskapsrådet (Grant 2023–03275), and Hjärnfonden (Grant FO2022‐0072).

## Conflicts of Interest

The authors declare no conflicts of interest.

## Supporting information

Additional figures, material and methods, as well as NMR spectra, can be found in the supporting information. The authors have cited additional references within the Supporting Information [37].

## Data Availability

The data that support the findings of this study are available from the corresponding author upon reasonable request.

## References

[1] S. H. W. Scheres , B. Ryskeldi‐Falcon , and M. Goedert , “Molecular Pathology of Neurodegenerative Diseases by Cryo‐EM of Amyloids,” Nature 621 (2023): 701–710, 10.1038/s41586-023-06437-2. 37758888

[2] L. Björk , T. Klingstedt , and K. P. R. Nilsson , “Thiophene‐Based Ligands: Design, Synthesis and Their Utilization for Optical Assignment of Polymorphic‐Disease‐Associated Protein Aggregates,” Chembiochem 24 (2023): e202300044, 10.1002/cbic.v24.11. PMC10404026 36891883

[3] M. Fändrich , S. Nyström , K. P. R. Nilsson , A. Böckmann , H. LeVine , and P. Hammarström , “Amyloid Fibril Polymorphism: A Challenge for Molecular Imaging and Therapy,” Journal of Internal Medicine 283 (2018): 218–237, 10.1111/joim.2018.283.issue-3. PMC5820168 29360284

[4] Y. Shi , W. Zhang , Y. Yang , et al., “Structure‐Based Classification of Tauopathies,” Nature 598 (2021): 359–563, 10.1038/s41586-021-03911-7. PMC7611841 34588692

[5] M. Kollmer , W. Close , L. Funk , et al., “Cryo‐EM Structure and Polymorphism of aβ Amyloid Fibrils Purified From Alzheimer’s Brain Tissue,” Nature Communications 10 (2019): 4760, 10.1038/s41467-019-12683-8. PMC6820800 31664019

[6] Y. Yang , D. Arseni , W. Zhang , et al., “Cryo‐EM Structures of Amyloid‐β 42 Filaments From Human Brains,” Science 375 (2022): 167–172, 10.1126/science.abm7285. PMC7612234 35025654

[7] Y. Yang , W. Zhang , A. G. Murzin , et al., “Cryo‐EM Structures of Amyloid‐β Filaments With the Arctic Mutation (E22G) From Human and Mouse Brains,” Acta Neuropathologica 145 (2023): 325–333, 10.1007/s00401-022-02533-1. PMC9925504 36611124

[8] Y. Yang , A. G. Murzin , S. Peak‐Chew , et al., “Cryo‐EM Structures of aβ40 Filaments From the Leptomeninges of Individuals With Alzheimer’s Disease and Cerebral Amyloid Angiopathy,” Acta Neuropathologica Communications 11 (2023): 191, 10.1186/s40478-023-01694-8. PMC10694933 38049918

[9] A. W. P. Fitzpatrick , B. Falcon , S. He , et al., “Cryo‐EM Structures of Tau Filaments From Alzheimer’s Disease,” Nature 547 (2017): 185–190, 10.1038/nature23002. PMC5552202 28678775

[10] B. Falcon , W. Zhang , A. G. Murzin , et al., “Structures of Filaments From Pick’s Disease Reveal a Novel Tau Protein Fold,” Nature 561 (2018): 137–140, 10.1038/s41586-018-0454-y. PMC6204212 30158706

[11] B. Falcon , J. Zivanov , W. Zhang , et al., “Novel Tau Filament Fold in Chronic Traumatic Encephalopathy Encloses Hydrophobic Molecules,” Nature 568 (2019): 420–423, 10.1038/s41586-019-1026-5. PMC6472968 30894745

[12] W. Zhang , A. Tarutani , K. L. Newell , et al., “Novel Tau Filament Fold in Corticobasal Degeneration,” Nature 580 (2020): 283–287, 10.1038/s41586-020-2043-0. PMC7148158 32050258

[13] W. Qiang , W. M. Yau , J. X. Lu , J. Collinge , and R. Tycko , “Structural Variation in Amyloid‐β Fibrils From Alzheimer’s Disease Clinical Subtypes,” Nature 541 (2017): 217–221, 10.1038/nature20814. PMC5233555 28052060

[14] J. X. Lu , W. Qiang , W. M. Yau , C. D. Schwieters , S. C. Meredith , and R. Tycko , “Molecular Structure of β‐Amyloid Fibrils in Alzheimer’s Disease Brain Tissue,” Cell 154 (2013): 1257–1268, 10.1016/j.cell.2013.08.035. PMC3814033 24034249

[15] T. Klingstedt , B. Ghetti , J. L. Holton , H. Ling , K. P. R. Nilsson , and M. Goedert , “Luminescent Conjugated Oligothiophenes Distinguish Between α‐Synuclein Assemblies of Parkinson’s Disease and Multiple System Atrophy,” Acta Neuropathologica Communications 7 (2019): 193, 10.1186/s40478-019-0840-1. PMC6892142 31796099

[16] A. Åslund , C. J. Sigurdson , T. Klingstedt , et al., “Novel Pentameric Thiophene Derivatives for In Vitro and In Vivo Optical Imaging of a Plethora of Protein Aggregates in Cerebral Amyloidoses,” ACS Chemical Biology 4 (2009): 673–684, 10.1021/cb900112v. PMC2886514 19624097

[17] T. Klingstedt , A. Åslund , R. A. Simon , et al., “Synthesis of a Library of Oligothiophenes and Their Utilization as Fluorescent Ligands for Spectral Assignment of Protein Aggregates,” Organic & Biomolecular Chemistry 9 (2011): 8356–8370, 10.1039/c1ob05637a. PMC3326384 22051883

[18] T. Klingstedt , C. Blechschmidt , A. Nogalska , et al., “Luminescent Conjugated Oligothiophenes for Sensitive Fluorescent Assignment of Protein Inclusion Bodies,” Chembiochem 14 (2013): 607–616, 10.1002/cbic.v14.5. PMC3743175 23450708

[19] T. Klingstedt , L. Lantz , H. Shirani , et al., “Thiophene‐Based Ligands for Specific Assignment of Distinct aβ Pathologies in Alzheimer’s Disease,” ACS Chemical Neuroscience 15 (2024): 1581–1595, 10.1021/acschemneuro.4c00021. PMC10995944 38523263

[20] J. Rasmussen , J. Mahler , N. Beschorner , et al., “Amyloid Polymorphisms Constitute Distinct Clouds of Conformational Variants in Different Etiological Subtypes of Alzheimer’s Disease,” Proceedings of the National Academy of Sciences of the United States of America 114 (2017): 13018–13023, 10.1073/pnas.1713215114. PMC5724274 29158413

[21] H. Liu , C. Kim , T. Haldiman , et al., “Distinct Conformers of Amyloid Beta Accumulate in the Neocortex of Patients With Rapidly Progressive Alzheimer’s Disease,” Journal of Biological Chemistry 297 (2021): 101267, 10.1016/j.jbc.2021.101267. PMC8531671 34599965

[22] T. Klingstedt , H. Shirani , J. Mahler , et al., “Distinct Spacing Between Anionic Groups: An Essential Chemical Determinant for Achieving Thiophene‐Based Ligands to Distinguish β‐Amyloid or Tau Polymorphic Aggregates,” Chemistry 21 (2015): 9072–9082, 10.1002/chem.201500556. PMC4517144 26013403

[23] S. Nyström , K. M. Psonka‐Antonczyk , P. G. Ellingsen , et al., “Evidence for Age‐Dependent In Vivo Conformational Rearrangement Within aβ Amyloid Deposits,” ACS Chemical Biology 8 (2013): 1128–1133, 10.1021/cb4000376. 23521783

[24] T. Klingstedt , H. Shirani , F. Parvin , et al., “Dual‐Ligand Fluorescence Microscopy Enables Chronological and Spatial Histological Assignment of Distinct Amyloid‐β Deposits,” Journal of Biological Chemistry 301 (2025): 108032, 10.1016/j.jbc.2024.108032. PMC11731580 39615691

[25] H. Shirani , H. Appelqvist , M. Bäck , T. Klingstedt , N. J. Cairns , and K. P. R. Nilsson , “Synthesis of Thiophene‐Based Optical Ligands That Selectively Detect Tau Pathology in Alzheimer’s Disease,” Chemistry – A European Journal 23 (2017): 17127–17135, 10.1002/chem.v23.67. PMC5928317 28926133

[26] L. Björk , H. Shirani , Y. Todarwal , et al., “Distinct Heterocyclic Moieties Govern the Selectivity of Thiophene‐Vinylene‐Based Ligands Towards aβ or Tau Pathology in Alzheimer’s Disease,” European Journal of Organic Chemistry 26 (2023): e202300583, 10.1002/ejoc.v26.41. PMC10997339 38585413

[27] X. Wu , H. Shirani , R. Vidal , et al., “Distinct Chemical Determinants Are Essential for Achieving Ligands for Superior Optical Detection of Specific Amyloid‐β Deposits in Alzheimer’s Disease,” ChemistryOpen 13 (2024): e202400186, 10.1002/open.v13.12. PMC11625938 39508558

[28] Y. Todarwal , C. Gustafsson , N. N. Thi Minh , et al., “Tau Protein Binding Modes in Alzheimer’s Disease for Cationic Luminescent Ligands,” Journal of Physical Chemistry B 125 (2021): 11628–11636, 10.1021/acs.jpcb.1c06019. PMC8558859 34643404

[29] Y. Tao , W. Xia , Q. Zhao , et al., “Structural Mechanism for Specific Binding of Chemical Compounds to Amyloid Fibrils,” Nature Chemical Biology 19 (2023): 1235–1245, 10.1038/s41589-023-01370-x. 37400537

[30] L. Björk , M. Bäck , L. Lantz , et al., “Proteophenes ‐ Amino Acid Functionalized Thiophene‐Based Fluorescent Ligands for Visualization of Protein Deposits in Tissue Sections With Alzheimer’s Disease Pathology,” Chemistry – A European Journal 28 (2022): e202201557, 10.1002/chem.v28.62. PMC9643645 35950816

[31] T. Klingstedt , H. Shirani , B. Ghetti , R. Vidal , and K. P. R. Nilsson , “Thiophene‐Based Optical Ligands That Selectively Detect aβ Pathology in Alzheimer’s Disease,” Chembiochem 22 (2021): 2568–2581, 10.1002/cbic.v22.15. PMC8409278 34101954

[32] K. Cao , M. Farahi , M. Dakanali , et al., “Aminonaphthalene 2‐Cyanoacrylate (ANCA) Probes Fluorescently Discriminate Between Amyloid‐β and Prion Plaques in Brain,” Journal of the American Chemical Society 134 (2012): 17338–17341, 10.1021/ja3063698. PMC3480552 22866977

[33] R. A. Simon , H. Shirani , K. O. A. Åslund , et al., “Pentameric Thiophene‐Based Ligands that Spectrally Discriminate Amyloid‐β and Tau Aggregates Display Distinct Solvatochromism and Viscosity‐Induced Spectral Shifts,” Chemistry – A European Journal 20 (2014): 12537–12543, 10.1002/chem.v20.39. PMC4221846 25111601

[34] F. Würthner , T. E. Kaiser , and C. R. Saha‐Möller , “J‐Aggregates: From Serendipitous Discovery to Supramolecular Engineering of Functional Dye Materials,” Angewandte Chemie, International Edition 50 (2011): 3376–3410, 10.1002/anie.v50.15. 21442690

[35] N. J. Hestand and F. C. Spano , “Expanded Theory of H‐ and J‐Molecular Aggregates: The Effects of Vibronic Coupling and Intermolecular Charge Transfer,” Chemical Reviews 118 (2018): 7069–7163, 10.1021/acs.chemrev.7b00581. 29664617

[36] Y. Shi , A. G. Murzin , B. Falcon , et al., “Cryo‐EM Structures of Tau Filaments From Alzheimer’s Disease With PET Ligand APN‐1607,” Acta Neuropathologica 141 (2021): 697–708, 10.1007/s00401-021-02294-3. PMC8043864 33723967

[37] K. Rurack and M. Spieles , “Fluorescence Quantum Yields of a Series of Red and Near‐Infrared Dyes Emitting at 600‐1000 Nm,” Analytical Chemistry 83 (2011): 1232–1242, 10.1021/ac101329h. 21250654

