# Supplementary material for: Amino Acid Functionalization Influences the Binding of Thiophene‐Vinylene‐Benzothiazolium‐Based Fluorescent Ligands to Protein Deposits in Tissue Sections With Alzheimer’s Disease Pathology
Source: ChemMedChem. 2026 Nov 3;21(21):e70558. doi: 10.1002/cmdc.70558 (PMC13731923; doi:10.1002/cmdc.70558)

Amino Acid Functionalization Influences the Binding of Thiophene-Vinylene-Benzothiazolium-Based Fluorescent Ligands to Protein Deposits in Tissue Sections with Alzheimer's Disease Pathology

Linnea Björk, Ruben Vidal, Bernardino Ghetti, Mikael Lindgren
Therése Klingstedt, K. Peter R. Nilsson

Supporting Information

Table of contents:

Supplementary figures 2

Material and Methods 14

NMR-spectra 23

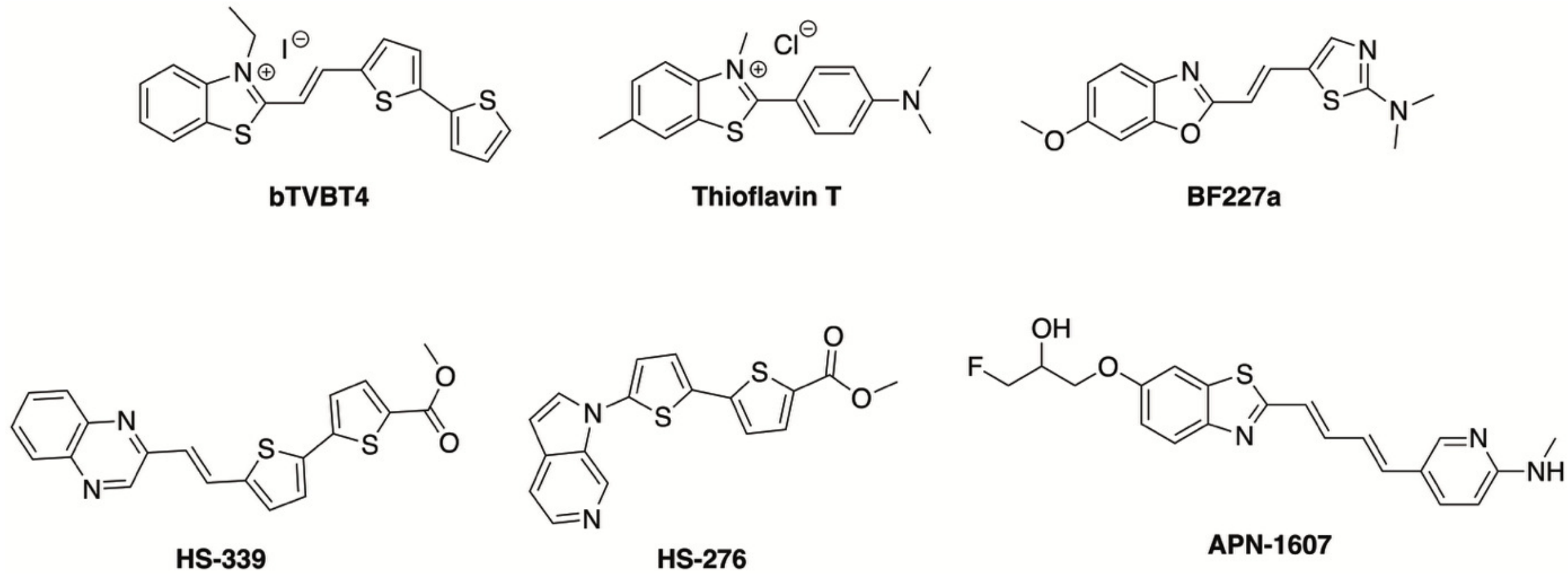

Supplemental Figure S1. Chemical structure of ligands bTVBT4, Thioflavin T, BF227a, HS-339, HS-276 and APN-1607.

Table S1. Quantium yield (QY) of the bTVBT based ligands in PBS, DMSO or THF. The values were determined using the slope method by plotting the integrated fluorescence intensity vs. the absorbance at λ_{ex} for a series of low weakly absorbing samples to avoid inner filter effects (OD < 0.1).[37] As a slope reference calibration data using Coumarin 153 in EtOH (QY = 0.54 ± 0.03) and/or MeOH (QY = 0.39 ± 0.03) were plotted. For representative data, see Figure S2.

Ligand	PBS	DMSO	THF
bTVBT2	0.61 ± 0.03	1.55 ± 0.08	0.80 ± 0.04
bTVBT-G-OMe	0.25 ± 0.02	1.50 ± 0.09	0.71 ± 0.09
bTVBT-A-OMe	0.33 ± 0.02	1.17 ± 0.07	0.58 ± 0.04
bTVBT-V-OMe	0.34 ± 0.02	1.46 ± 0.10	0.54 ± 0.08
bTVBT-E-diOMe	0.31 ± 0.03	1.35 ± 0.08	0.54 ± 0.04
bTVBT-Y-OMe	0.34 ± 0.04	1.61 ± 0.09	0.60 ± 0.06

A)

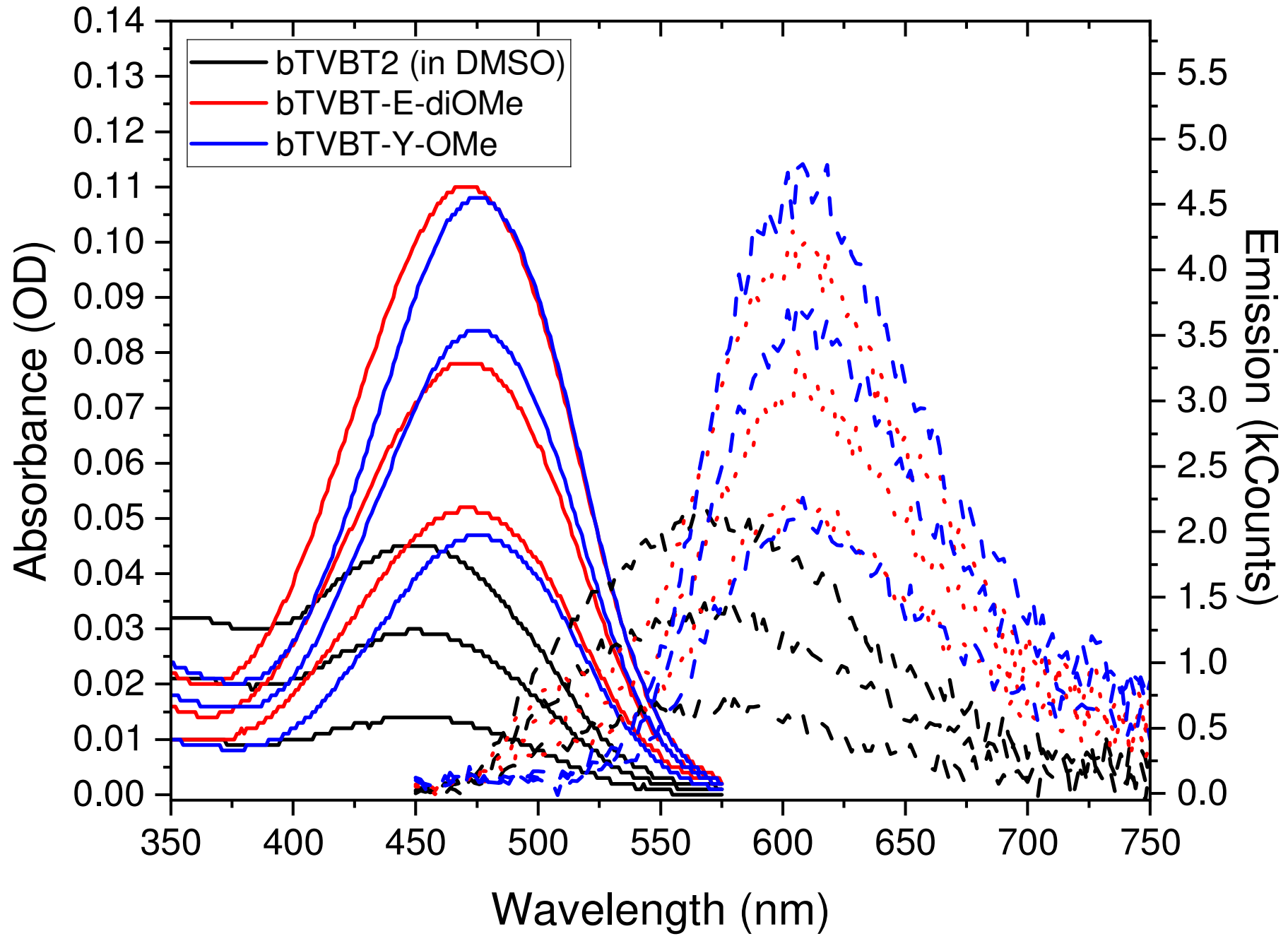

B)

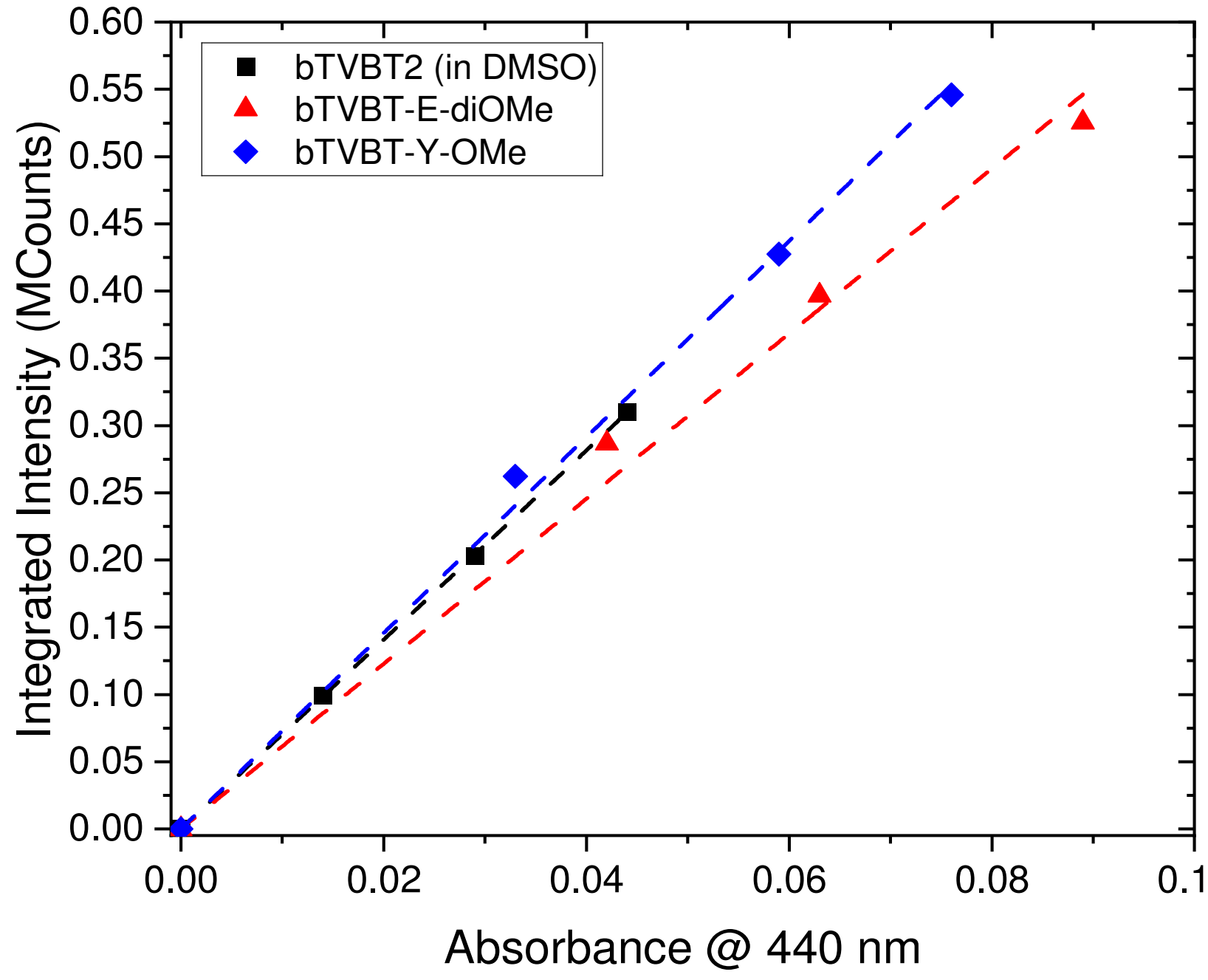

Supplemental Figure S2. (A) Representative absorption and emission spectra for quantum yield calculations using DMSO as solvent (λ_{ex} = 440 nm; slits 2 nm both for excitation and emission.). The fluorescence spectra were background subtracted using a 'solvent-only' sample with identical settings. (B) Associated slope plots: integrated fluorescence vs. absorbance at 440 nm. In general, the spectral features as well as the pertinent QY values, see Table S1, were very similar for all the ligands.

Table S2. Clinical parameters for the AD cases.

Case no	Age	Sex	Braak	Thal	PMI (h)
1	80	Female	5	3	9.0
2	79	Female	6	4	3.0
3	63	Male	6	5	6.0

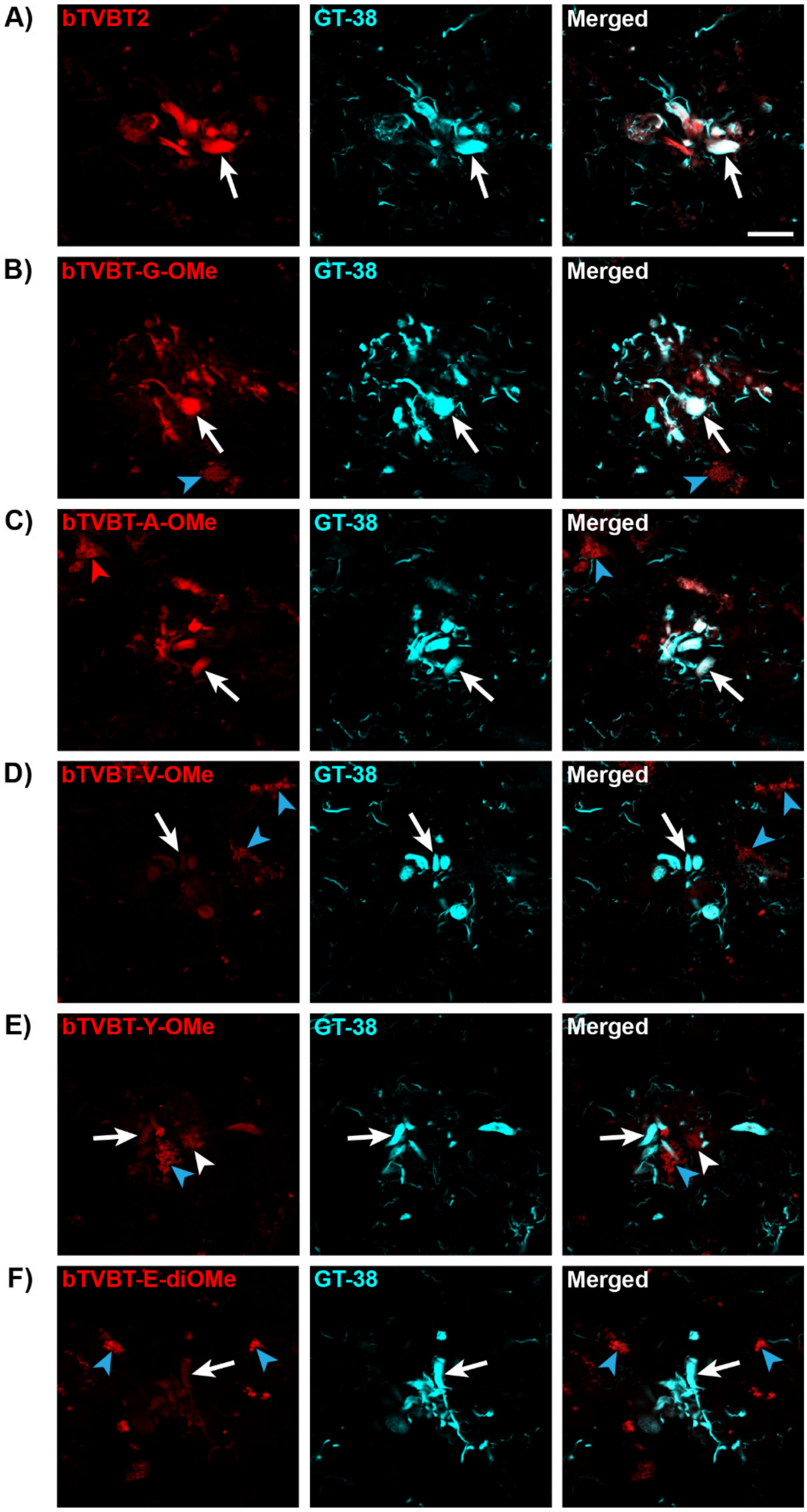

Supplemental Figure 3. Fluorescence images of sAD brain tissue section (case 1) stained with 100 nM bTVBT2 (A) or amino-acid functionalized bTVBT ligand bTVBT-G-OMe (B), bTVBT-A-OMe (C), bTVBT-V-OMe (D), bTVBT-Y-OMe (E) or bTVBT-E-diOMe (F), all in red, together with GT-38 anti-tau antibody in cyan. bTVBT2, bTVBT-G-OMe and bTVBT-A-OMe show strong staining of DNs (white arrow). Autofluorescence from lipofuscin (blue arrowhead) can also be seen in the channel used to collect the fluorescence from the ligands, but the granular appearance makes it possible to distinguish lipofuscin from DNs. All amino functionalized bTVBTs, except bTVBT-E-diOMe, showed staining of a limited number of Aβ plaques. For all ligands except bTVBT-Y-OMe, the fluorescence from the labelled Aβ plaques was much weaker than from the tau deposits. When stained with bTVBT-Y-OMe, the fluorescence intensity was similar from Aβ and tau, and the labelling of Aβ can be seen in E (white arrowhead). Scalebar, 20 μm.

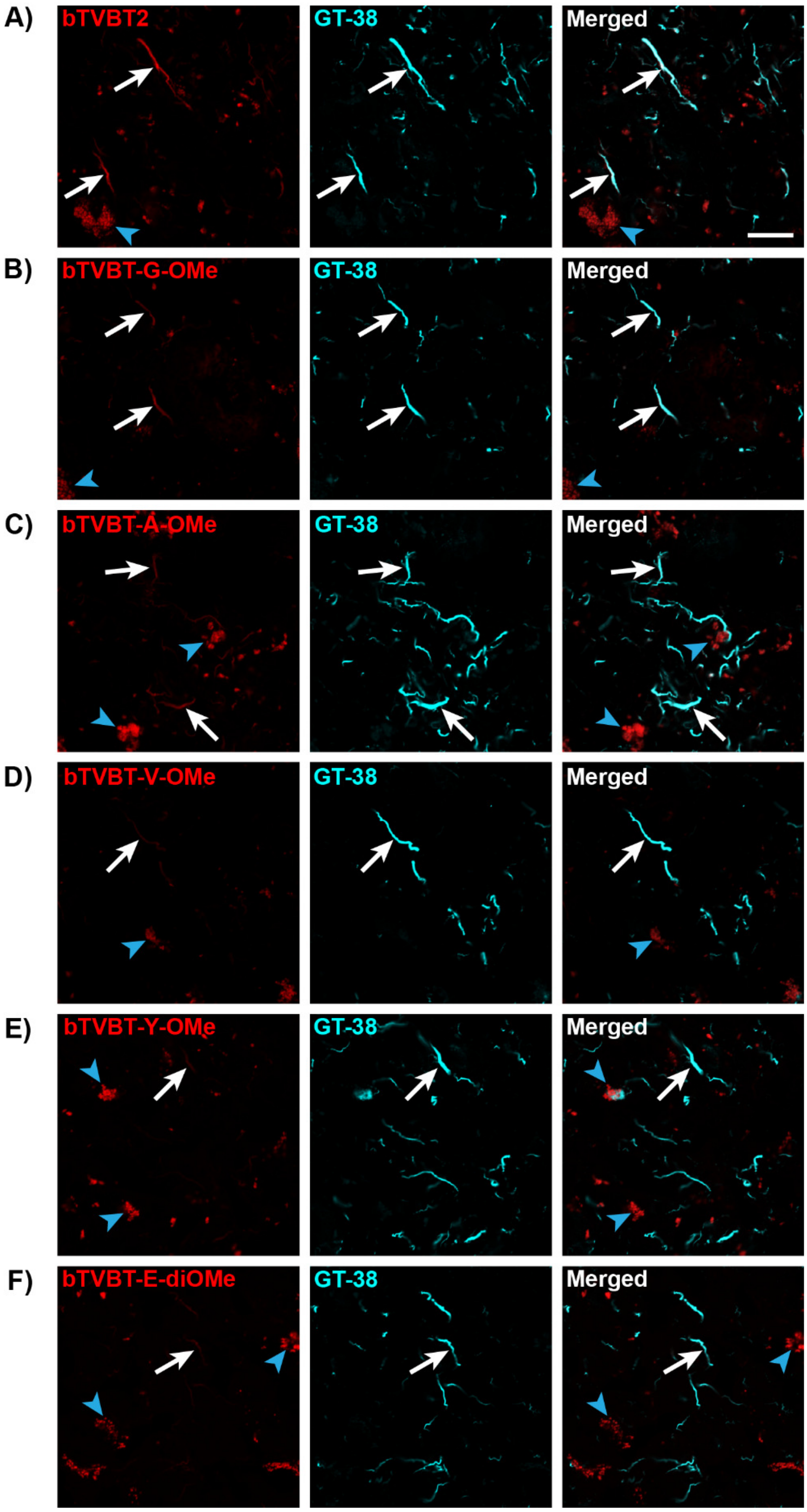

Supplemental Figure 4. Fluorescence images of sAD brain tissue section (case 1) stained with 100 nM bTVBT2 (A) or amino-acid functionalized bTVBT ligand bTVBT-G-OMe (B), bTVBT-A-OMe (C), bTVBT-V-OMe (D), bTVBT-Y-OMe (E) or bTVBT-E-diOMe (F), all in red, together with GT-38 anti-tau antibody in cyan. bTVBT2, bTVBT-G-OMe and bTVBT-A-OMe show staining of NTs (white arrow). Autofluorescence from lipofuscin (blue arrowhead) can also be seen in the channel used to collect the fluorescence from the ligands, but the granular appearance makes it possible to distinguish lipofuscin from NTs. Scalebar, 20 µm.

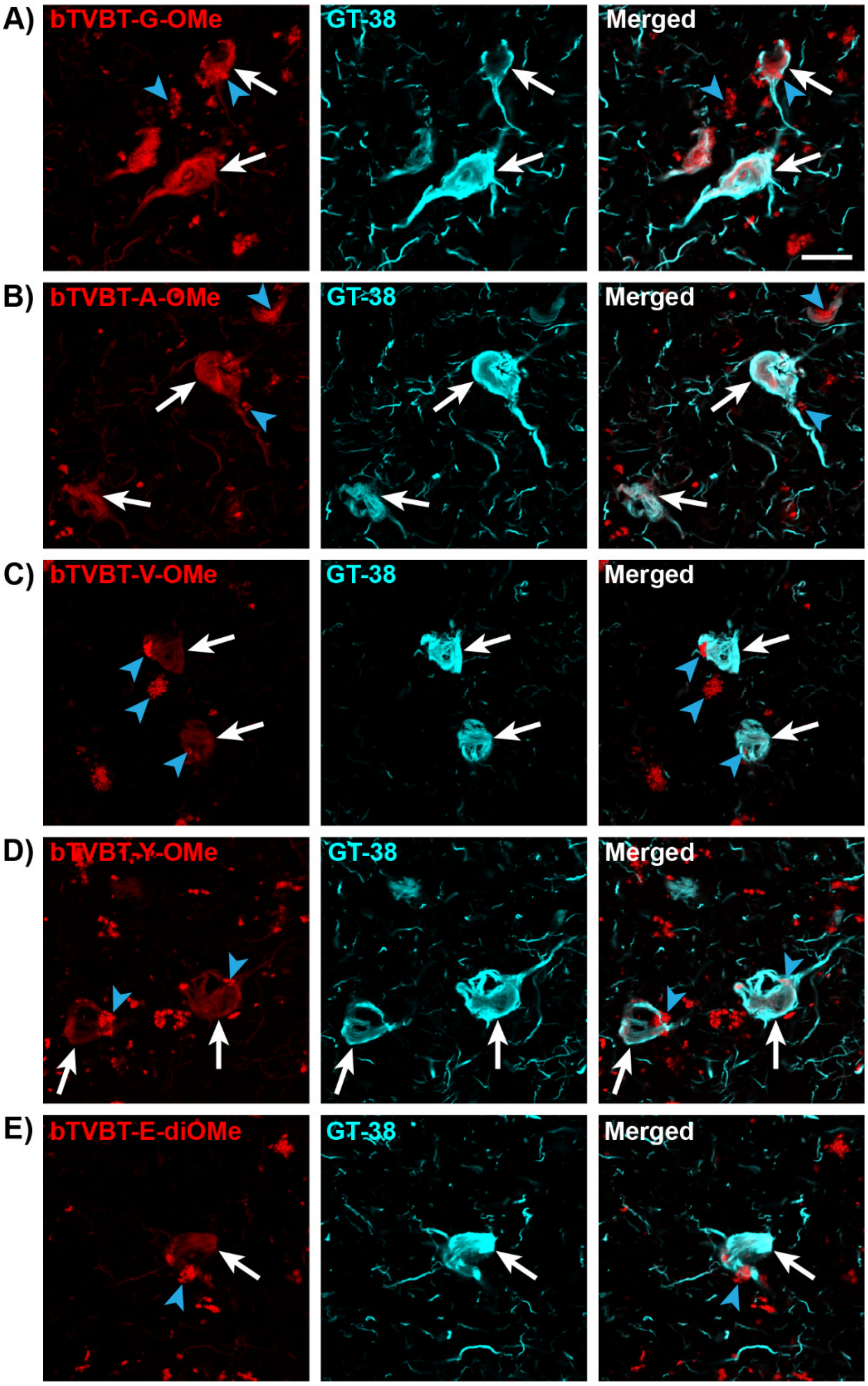

Supplemental Figure 5. Fluorescence images of sAD brain tissue section (case 2) stained with 100 nM amino-acid functionalized 100 nM bTVBT ligands bTVBT-G-OMe (A), bTVBT-A-OMe (B), bTVBT-V-OMe (C), bTVBT-Y-OMe (D) or bTVBT-E-diOMe (E), all in red, together with GT-38 anti-tau antibody in cyan. Representative NFTs are indicated with white arrow. Autofluorescence from lipofuscin (blue arrowhead) can also be seen in the channel used to collect the fluorescence from the ligands, but the granular appearance makes it possible to distinguish lipofuscin from NFTs. Scalebar, 20 µm.

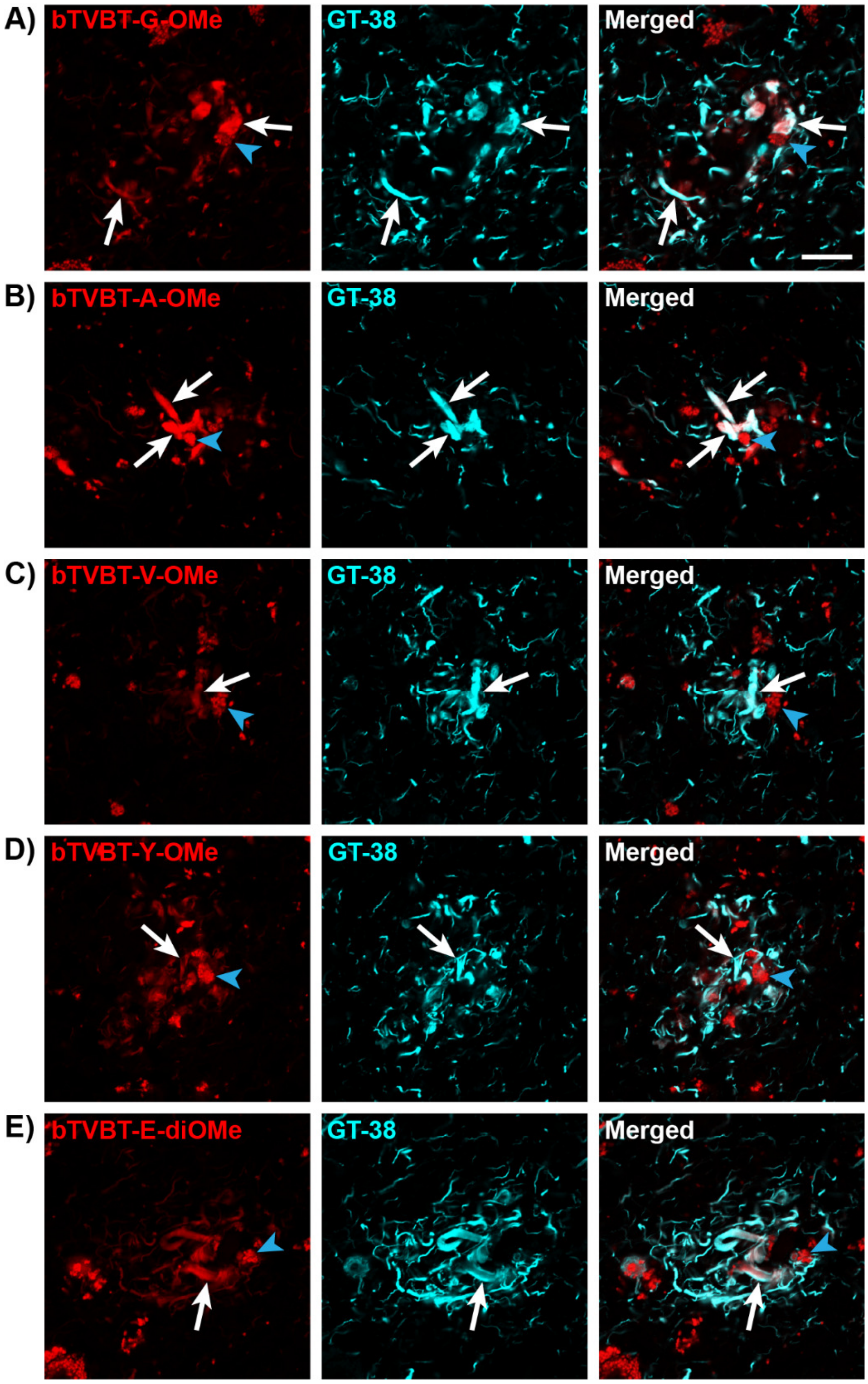

Supplemental Figure 6. Fluorescence images of sAD brain tissue section (case 2) stained with 100 nM amino-acid functionalized bTVBT ligand bTVBT-G-OMe (A), bTVBT-A-OMe (B), bTVBT-V-OMe (C), bTVBT-Y-OMe (D) or bTVBT-E-diOMe (E), all in red, together with GT-38 anti-tau antibody in cyan. Representative DNs are indicated withwhite arrows. Autofluorescence from lipofuscin (blue arrowhead) can also be seen in the channel used to collect the fluorescence from the ligands, but the granular appearance makes it possible to distinguish lipofuscin from DNs. Scalebar, 20 µm.

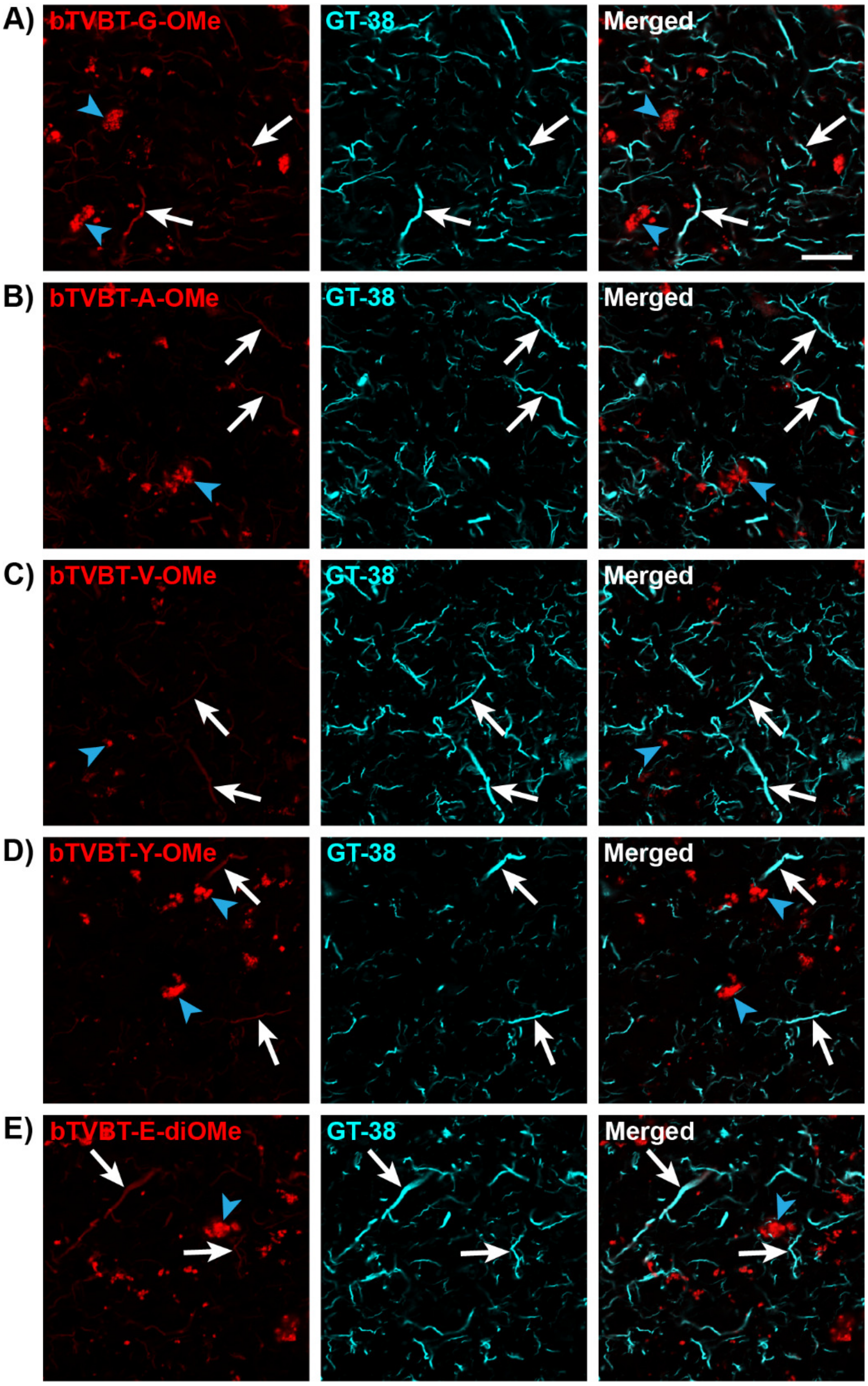

Supplemental Figure 7. Fluorescence images of sAD brain tissue section (case 2) stained with 100 nM amino-acid functionalized bTVBT ligand bTVBT-G-OMe (A), bTVBT-A-OMe (B), bTVBT-V-OMe (C), bTVBT-Y-OMe (D) or bTVBT-E-diOMe (E), all in red, together with GT-38 anti-tau antibody in cyan. Representative NTs are indicated with white arrows. Autofluorescence from lipofuscin (blue arrowhead) can also be seen in the channel used to collect the fluorescence from the ligands, but the granular appearance makes it possible to distinguish lipofuscin from NTs. Scalebar, 20 µm.

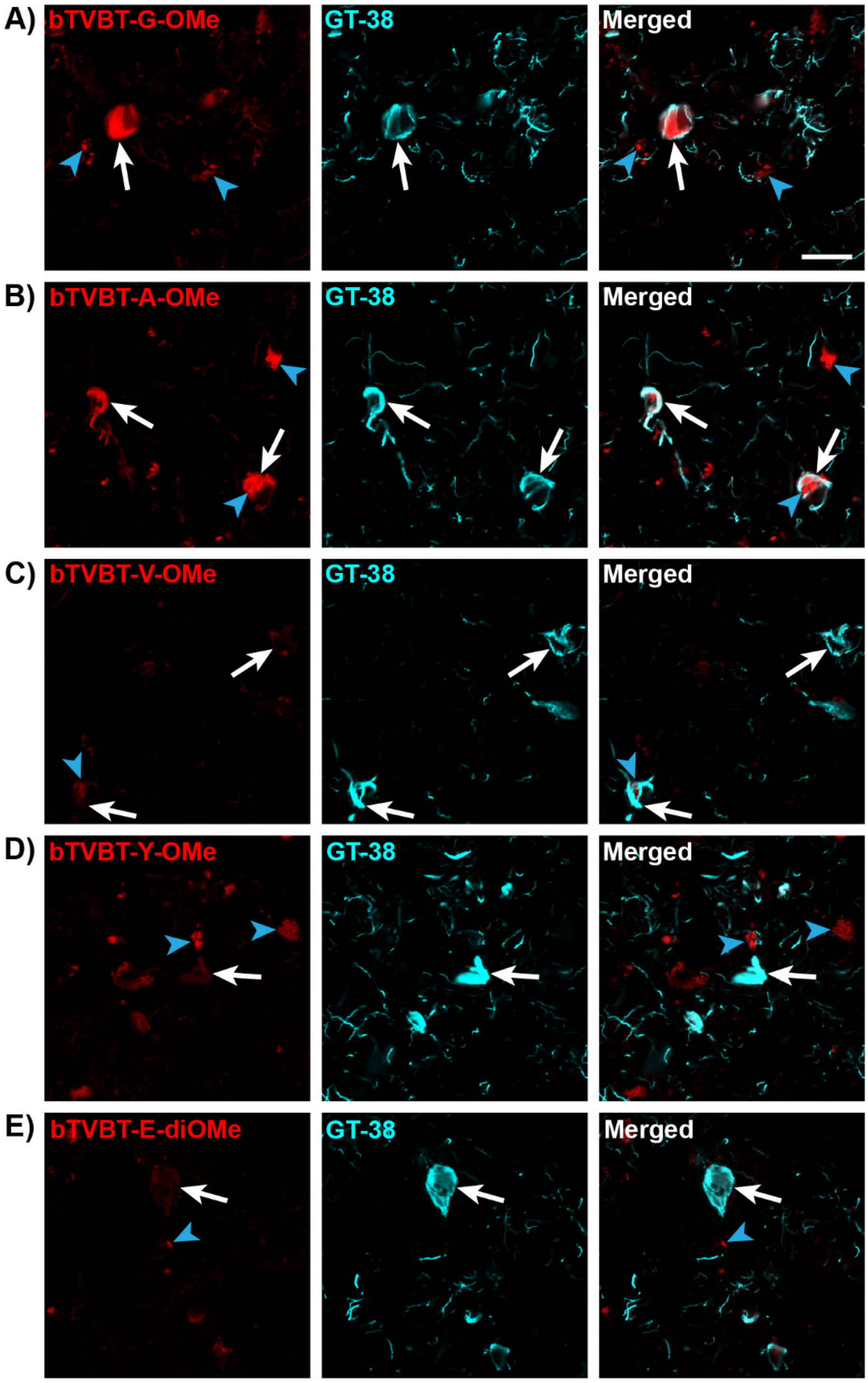

Supplemental Figure 8. Fluorescence images of sAD brain tissue section (case 3) stained with 100 nM amino-acid functionalized 100 nM bTVBT ligands bTVBT-G-OMe (A), bTVBT-A-OMe (B), bTVBT-V-OMe (C), bTVBT-Y-OMe (D) or bTVBT-E-diOMe (E), all in red, together with GT-38 anti-tau antibody in cyan. Representative NFTs are indicated with white arrow. Autofluorescence from lipofuscin (blue arrowhead) can also be seen in the channel used to collect the fluorescence from the ligands, but the granular appearance makes it possible to distinguish lipofuscin from NFTs. Scalebar, 20 µm.

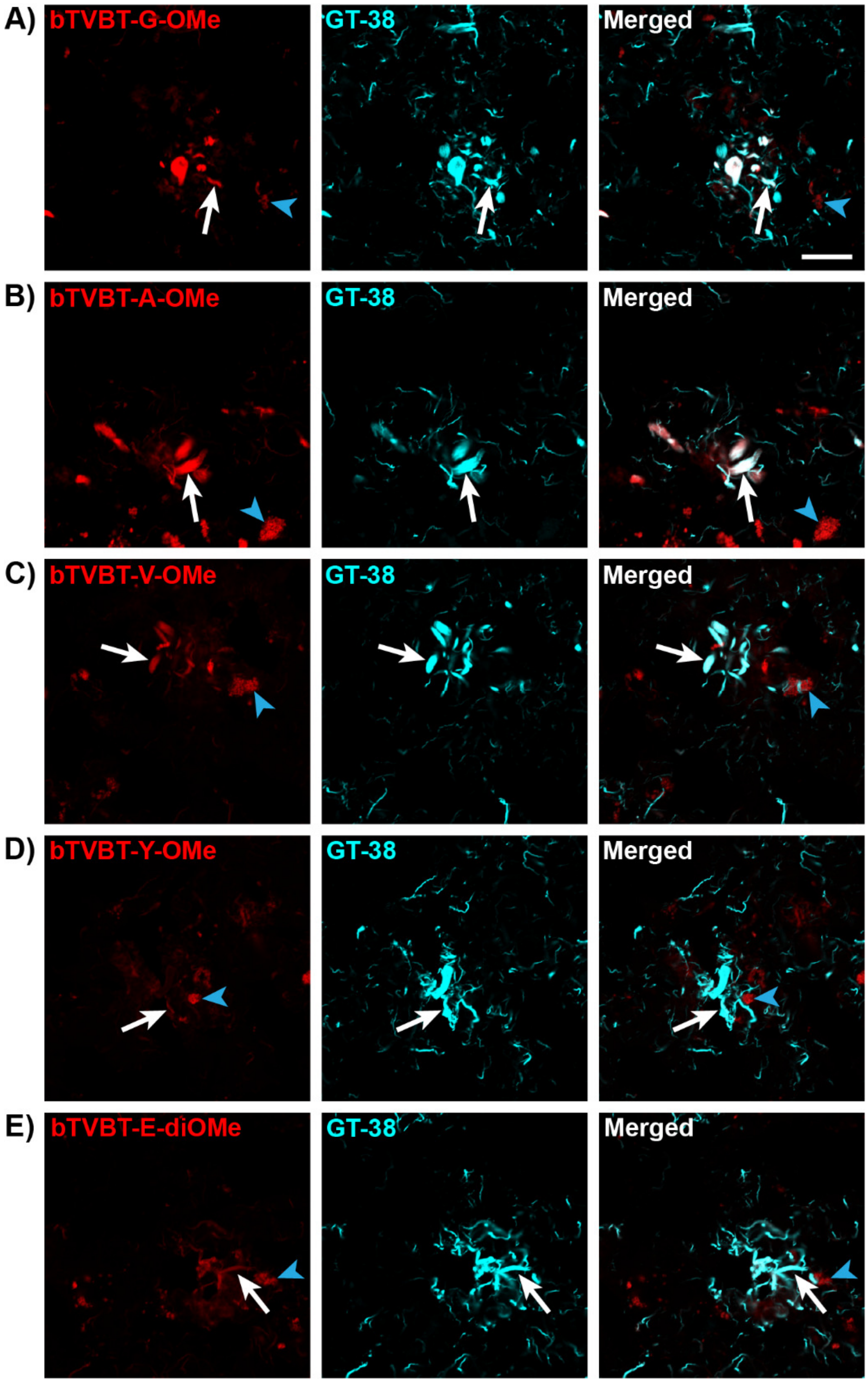

Supplemental Figure 9. Fluorescence images of sAD brain tissue section (case 3) stained with 100 nM amino-acid functionalized bTVBT ligand bTVBT-G-OMe (A), bTVBT-A-OMe (B), bTVBT-V-OMe (C), bTVBT-Y-OMe (D) or bTVBT-E-diOMe (E), all in red, together with GT-38 anti-tau antibody in cyan. Representative DNs are indicated withwhite arrows. Autofluorescence from lipofuscin (blue arrowhead) can also be seen in the channel used to collect the fluorescence from the ligands, but the granular appearance makes it possible to distinguish lipofuscin from DNs. Scalebar, 20 µm.

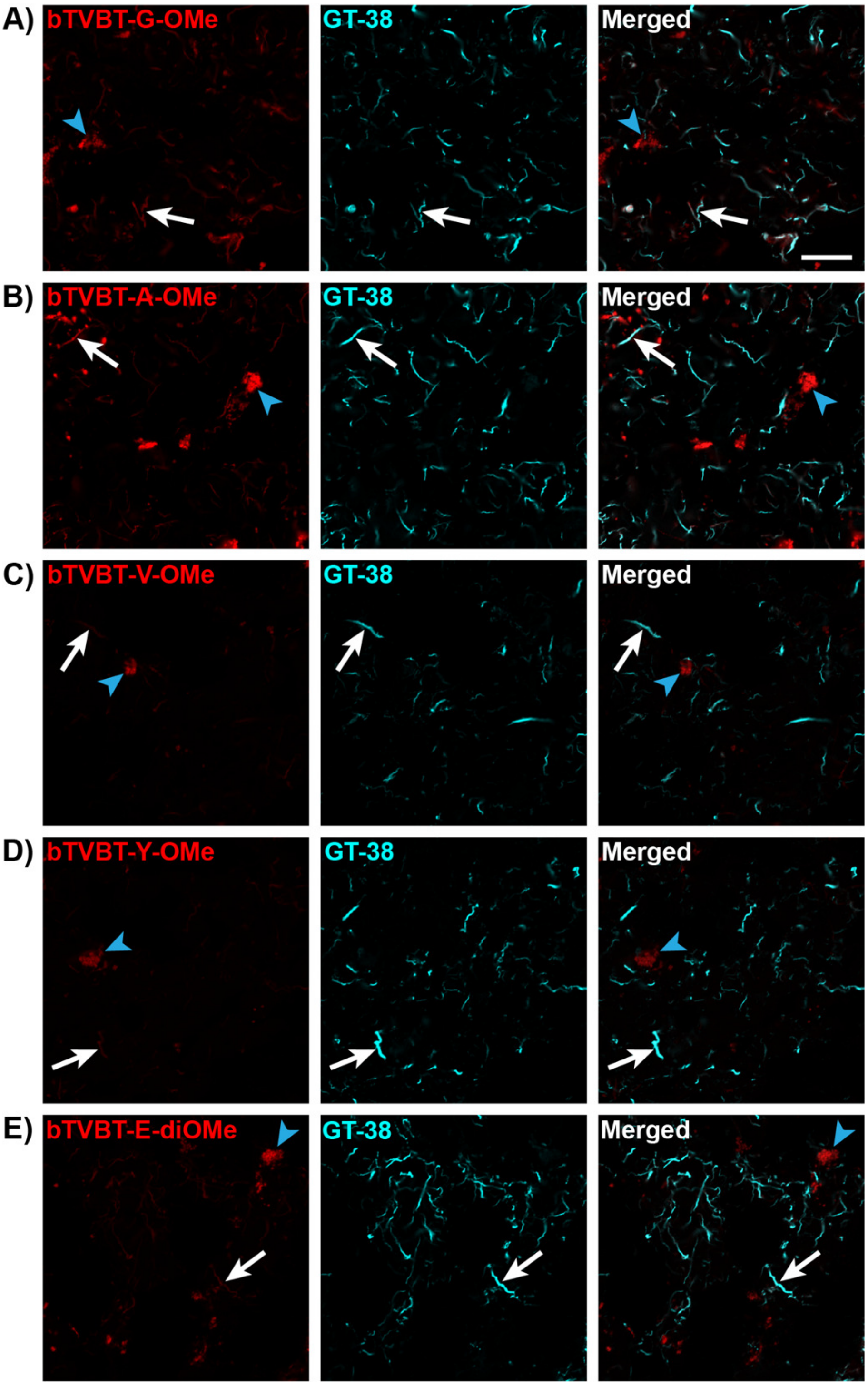

Supplemental Figure 10. Fluorescence images of sAD brain tissue section (case 3) stained with 100 nM amino-acid functionalized bTVBT ligand bTVBT-G-OMe (A), bTVBT-A-OMe (B), bTVBT-V-OMe (C), bTVBT-Y-OMe (D) or bTVBT-E-diOMe (E), all in red, together with GT-38 anti-tau antibody in cyan. Representative NTs are indicated with white arrows. Autofluorescence from lipofuscin (blue arrowhead) can also be seen in the channel used to collect the fluorescence from the ligands, but the granular appearance makes it possible to distinguish lipofuscin from NTs. Scalebar, 20 μm.

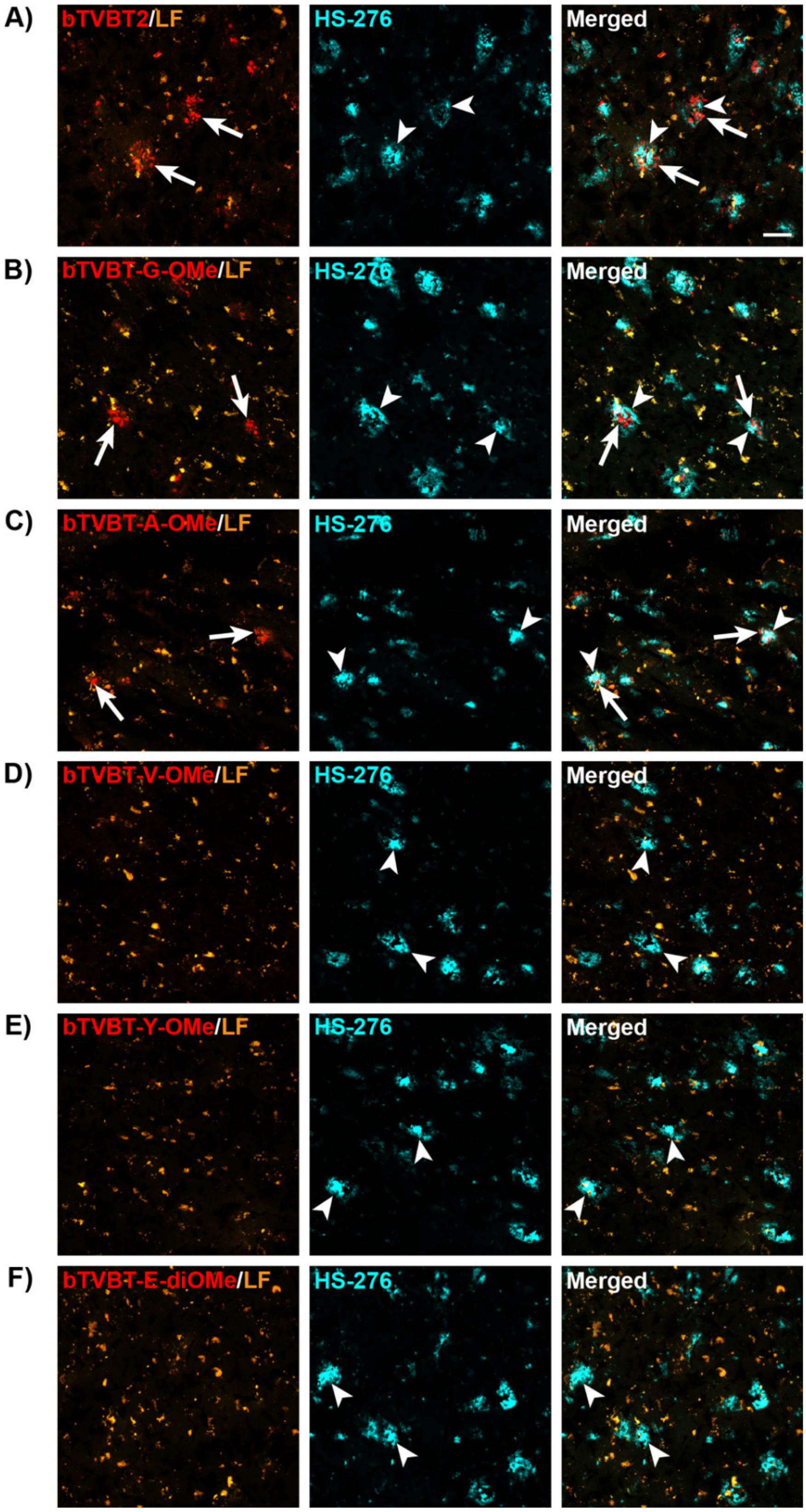

Supplemental Figure 11. Fluorescence images of sAD brain tissue section (case 1) stained with 100 nM bTVBT2 (A) or amino-acid functionalized bTVBT ligand bTVBT-G-OMe (B), bTVBT-A-OMe (C), bTVBT-V-OMe (D), bTVBT-Y-OMe (E) or bTVBT-E-diOMe (F), all in red, together with the Aβ aggregate selective ligand HS-276 in cyan. bTVBT2, bTVBT-G-OMe and bTVBT-A-OMe show staining of DNs (white arrow), whereas in sections stained with bTVBT-V-OMe, bTVBT-Y-OMe or bTVBT-E-diOMe together with HS-276, only HS-276 staining of Aβ plaques (white arrowhead) can be seen. The orange structures represent autofluorescent lipofuscin. Scalebar, 20 μm.

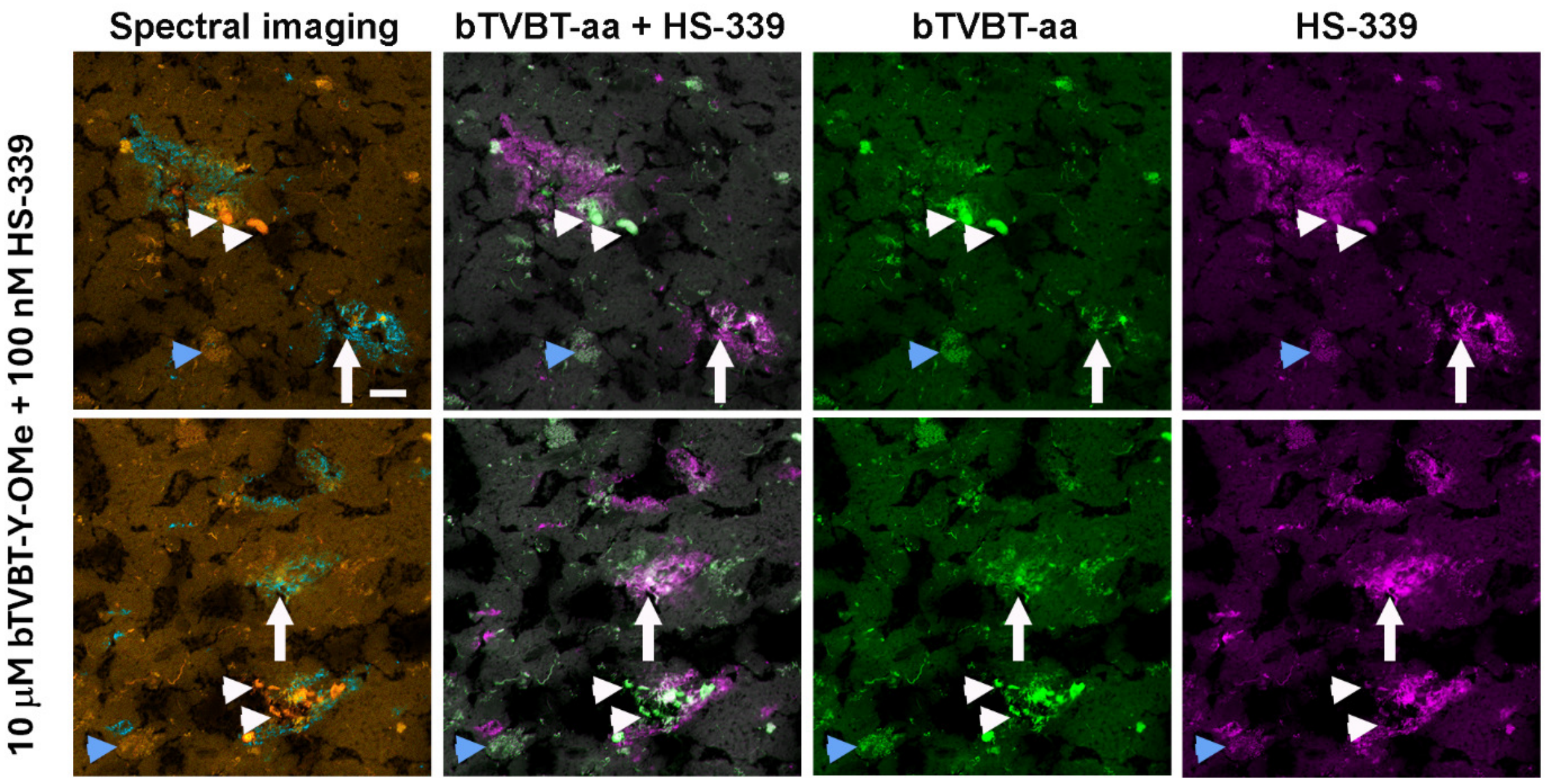

Supplemental Figure 12. Fluorescence images of an sAD brain tissue section (case 1) pre-stained with 10 μM bTVBT-Y-OMe followed by 100 nM HS-339. In the left panel, which shows spectral imaging results, HS-339 labelled aggregated pathologies are seen in blue whereas protein deposits labelled by bTVBT-Y-OMe display yellow/orange emission. In channel mode images, HS-339 labelled aggregated pathologies are seen in magenta whereas protein deposits labelled by bTVBT-Y-OMe are displayed in green. Representative Aβ deposits or tau aggregates are indicated with white arrows or white arrowheads, respectively. Autofluorescence from lipofuscin is indicated with blue arrowheads. Scalebar, 20 μm.

Materials and Methods

Synthesis of amino acid functionalized bi-thiophene-vinylene-benzothiazolium (bTVBT) based ligands

Synthesis of bTVBT2, HS-339 and HS-276 have been published elsewhere.[25,26,31] NMR-spectra of newly synthesized compounds can be found further down in the Supporting information.

General methods

NMR spectra were recorded on a Bruker instrument (^{1}H 500 MHz, ^{13}C 126 MHz) and chemical shifts were designated on the ppm scale using residual undeuterated solvent peak as reference. All reagents and solvents were purchased from commercial sources and used as received without further purification. TLC was carried out on Merck pre-coated 60 F254 aluminium plates using UV-light (λ=254 nm and 366 nm) for visualization and column chromatography was carried out on silica gel (60 Å, 0.040-0.063 mm, 230-400 mesh particle size, Sigma Aldrich, Merck KGaA). Purification on automated flash column chromatography system was accomplished by Biotage® Selekt instrument through Biotage® Sfär silica or C18 columns. Analytical high-performance liquid chromatography/mass spectrometry (HPLC/MS) was performed on a Waters system equipped with a Waters 1525 gradient pump, 2998 Photodiode Array Detector, 2424 Evaporative Light Scattering Detector, SQD 2 Mass Detector and an XBridge® C18 column (4.6×50 mm, 3.5 µm). The purity of the final compounds was assessed by HPLC–ELSD, and all final compounds showed a chromatographic purity of >95%. LC-QTOF-MS analysis for high resolution MS data was conducted on an Agilent 1290 Infinity ultra-high-performance LC system coupled to an Agilent 6550 iFunnel QTOF. A 6-min gradient was used on an EclipsePlusC18 RRHD column (1.8 uM, 2.1 X 50 mm), using 0.1 % formic acid in water (A) and 0.1 % formic acid in acetonitrile (B) with a gradient of A : B from 80 : 20 to 5 : 95.

General procedure for amide coupling

To a solution of the 5-bromothiophene-2-carboxylic acid in DMF (40 mL/mmol) and the desired amino acid (1.5 equiv.), DIPEA (3 equiv.) and HATU (1.5 equiv.) were added, and the reaction was left to stir until finished. Extracting µL aliquots analyzed by HPLC-MS monitored the reaction. The residue was extracted with ethyl acetate, washed with H_2O and Brine, dried over $MgSO_4$, and the solvent evaporated under reduced pressure. The crude product was

purified through automated flash column chromatography with appropriate solvents to yield the desired product.

General procedure for Suzuki-Miyaura coupling

A mixture of the thiophene bromide, (5-formylthiophen-2-yl)boronic acid, K_2CO_3 (5 equiv./halide), and PEPPS-IPr (5 mol %) in 1,4-dioxane/methanol (8 : 2, 8 mL/mmol) was heated to 80 °C until analysis on LC–MS indicated completion of reaction. After cooling to room temperature pH was adjusted using HCl (1M), and the residue was extracted with DCM, washed with water and brine. The combined organic phase was dried over $MgSO_4$, and the solvent was evaporated. The crude product was subjected to column chromatography, either manually or automated, with appropriate solvent to give the desired product.

General procedure for condensation reaction

A few drops of pyridine were added to a cold solution of the thiophene aldehyde (1 equiv.) and the 3-ethyl-2-methylbenzothiazolium iodide (1 equiv.) in anhydrous MeOH, under nitrogen flow. The mixture was refluxed until completion of the reaction (monitored by LC-MS). The solvent was evaporated in vacuo to provide a dark red solid, which was crystallized from appropriate solvent. The red solid was collected by filtration, washed with cold MeOH and dried in vacuum to afford desired final products. All final compounds were isolated as the E-isomer.

Br-thiophene-Glu-diOMe

5-bromothiophene-2-carboxylic acid (100 mg, 0.483 mmol) together with H-Glu(OMe)-OMe·HCl (153 mg, 0.724 mmol) were set for general procedure of amide coupling, before purification using heptane/ethyl acetate as eluent gave Br-thiophene-Glu-diOMe (161 mg, 92%) as a light-yellow oil.

^{1}H NMR (500 MHz, CD_3OD) δ 7.56 (d, *J* = 4.0 Hz, 1H), 7.16 (d, *J* = 4.0 Hz, 1H), 4.59 (dd, *J* = 9.5, 5.2 Hz, 1H), 3.74 (s, 3H), 3.65 (s, 3H), 2.48 (t, *J* = 7.3 Hz, 2H), 2.31 – 2.23 (m, 1H), 2.11-2.03 (m, 1H). ^{13}C NMR (126 MHz, CD_3OD) δ 174.8, 173.4, 163.2, 141.3, 132.4, 130.6, 119.5, 53.5, 52.9, 52.2, 31.2, 27.3. LCMS (ESI): m/z calcd for $C_{12}H_{14}BrNO_5S$ (M+H): 364.0 Found: 364.2

Formyl-bi-thiophene-Glu-diOMe

Br-thiophene-Glu-diOMe (52.9 mg, 0.145 mmol) together with (5-formylthiophen-2-yl)boronic acid (23.8 mg, 0.153 mmol) were set for general procedure of Suzuki-Miyaura

coupling, before purification by flash column chromatography using dichloromethane/methanol (4%) as eluent gave the pure formyl-bi-thiophene-Glu-diOMe (42.0 mg, 73%).

^{1}H NMR (500 MHz, $CDCl_3$) δ 9.88 (s, 1H), 7.68 (d, *J* = 4.0 Hz, 1H), 7.49 (d, *J* = 4.0 Hz, 1H), 7.31 (d, *J* = 4.0 Hz, 1H), 7.29 (d, *J* = 4.0 Hz, 1H), 7.13 (d, *J* = 7.3 Hz, 1H), 4.78-4.74 (m, 1H), 3.79 (s, 3H), 3.68 (s, 3H), 2.54 – 2.46 (m, 2H), 2.32 – 2.27 (m, 1H), 2.16 – 2.11 (m, 1H). ^{13}C NMR (126 MHz, $CDCl_3$) δ 182.7, 174.0, 172.3, 161.2, 145.7, 143.1, 140.8, 139.2, 137.2, 129.2, 126.3, 125.7, 52.9, 52.6, 52.2, 30.4, 27.0. LCMS (ESI): m/z calcd for $C_{17}H_{17}NO_6S_2$ (M+H): 396.1 Found: 396.3

bTVBT-Glu-diOMe

Formyl-bi-thiophene-Glu-diOMe (42.0 mg, 0.106 mmol) together with 3-ethyl-2-methylbenzothiazolium iodide (34.0 mg, 0.112 mmol) were set for general procedure of condensation reaction, before crystallization from methanol gave bTVBT-Glu-diOMe (36.6 mg, 50%) as a dark red solid. HPLC purity: >99%

^{1}H NMR (500 MHz, DMSO) δ 8.97 (d, *J* = 7.5 Hz, 1H), 8.42 (d, *J* = 15.4 Hz, 1H), 8.36 (d, *J* = 8.1 Hz, 1H), 8.24 (d, *J* = 8.4 Hz, 1H), 7.92 (d, *J* = 4.0 Hz, 1H), 7.88 (d, *J* = 4.0 Hz, 1H), 7.84 (d, *J* = 8.6 Hz, 1H), 7.77 (t, *J* = 7.7 Hz, 1H), 7.69 (d, *J* = 15.4 Hz, 1H), 7.65 (d, *J* = 4.0 Hz, 1H), 7.59 (d, *J* = 3.9 Hz, 1H), 4.90 (t, *J* = 7.3 Hz, 2H), 4.43 (ddd, *J* = 9.6, 7.5, 5.4 Hz, 1H), 3.65 (s, 3H), 2.45 (t, *J* = 7.4 Hz, 2H), 2.12 (dtt, *J* = 11.7, 8.4, 4.7 Hz, 1H), 1.99 (ddt, *J* = 14.0, 9.7, 7.1 Hz, 1H), 1.46 (t, *J* = 7.2 Hz, 3H). ^{13}C NMR (126 MHz, $CDCl_3$:CD_3OD) δ 173.7, 172.1, 170.0, 161.8, 144.9, 142.1, 140.6, 140.3, 139.1, 138.4, 137.6, 130.0, 129.7, 128.8, 127.8, 126.6, 126.5, 123.9, 115.6, 109.3, 52.3, 52.2, 51.7, 44.9, 30.2, 26.2, 14.0. HRMS (ESI): m/z calcd for $C_{27}H_{27}N_2O_5S_3^+$ (M+): 555.1077 Found: 555.1063

Br-thiophene-Tyr-OMe

5-bromothiophene-2-carboxylic acid (100 mg, 0.483 mmol) together with H-Tyr-OMe (141 mg, 0.724 mmol) were set for general procedure of amide coupling, before purification using heptane/ethyl acetate as eluent gave Br-thiophene-Tyr-OMe (148 mg, 80%) as a white powder.

^{1}H NMR (500 MHz, CD_3OD) δ 7.48 (d, *J* = 4.0 Hz, 1H), 7.12 (d, *J* = 4.0 Hz, 1H), 7.04 (d, *J* = 8.6 Hz, 2H), 6.69 (d, *J* = 8.6 Hz, 2H), 4.70 (dd, *J* = 9.2, 5.8 Hz, 1H), 3.70 (s, 3H), 3.15 (dd, *J* = 14.0, 5.8 Hz, 1H), 2.98 (dd, *J* = 14.0, 9.2 Hz, 1H). ^{13}C NMR (126 MHz, CD_3OD) δ 173.6, 163.0, 157.4, 141.4, 132.3, 131.2, 130.5, 128.9, 119.4, 116.3, 56.1, 52.7, 37.4. LCMS (ESI): m/z calcd for $C_{15}H_{14}BrNO_4S$ (M+H): 384.0 Found: 384.2

Formyl-bi-thiophene-Tyr-OMe

Br-thiophene-Tyr-OMe (30.0 mg, 0.0781 mmol) together with (5-formylthiophen-2-yl)boronic acid (12.2 mg, 0.0781 mmol) were set for general procedure of Suzuki-Miyaura coupling, before purification by flash column chromatography using dichloromethane/methanol (3%) as eluent gave the pure formyl-bi-thiophene-Tyr-OMe (28.6 mg, 88%) as a yellow-brown oil.

^{1}H NMR (500 MHz, $CDCl_3$) δ 9.85 (s, 1H), 7.67 (d, *J* = 4.0 Hz, 1H), 7.38 (d, *J* = 3.9 Hz, 1H), 7.27 (d, *J* = 4.0 Hz, 1H), 7.22 (d, *J* = 3.9 Hz, 1H), 6.97 (d, *J* = 8.4 Hz, 2H), 6.75 (d, *J* = 8.4 Hz, 2H), 6.65 (d, *J* = 7.9 Hz, 1H), 5.01 (dt, *J* = 7.9, 5.7 Hz, 1H), 3.78 (s, 3H), 3.18 (dd, *J* = 14.1, 5.7 Hz, 1H), 3.11 (dd, J = 14.1, 5.7 Hz, 1H). ^{13}C NMR (126 MHz, $CDCl_3$) δ 182.9, 172.3, 161.0, 155.5, 145.7, 143.0, 140.8, 138.8, 137.4, 130.5, 129.5, 127.1, 126.4, 125.8, 115.8, 53.9, 52.8, 37.3. LCMS (ESI): m/z calcd for $C_{20}H_{17}NO_5S_2$ (M+H): 416.1 Found: 416.2

bTVBT-Tyr-OMe

Formyl-bi-thiophene-Tyr-OMe (28.0 mg, 0.0674 mmol) together with 3-ethyl-2-methylbenzothiazolium iodide (20.6 mg, 0.0674 mmol) were set for general procedure of condensation reaction, before crystallization from methanol gave bTVBT-Tyr-OMe (15.6 mg, 33%) as a dark red solid. HPLC purity: >97%

^{1}H NMR (500 MHz, DMSO) δ 9.32 (s, 1H), 8.99 (d, *J* = 7.8 Hz, 1H), 8.41 (d, *J* = 15.5 Hz, 1H), 8.36 (d, *J* = 8.2 Hz, 1H), 8.23 (d, *J* = 8.2 Hz, 1H), 7.92 (d, *J* = 3.9 Hz, 1H), 7.83 (d, *J* = 3.9 Hz, 2H), 7.75 (t, *J* = 7.8 Hz, 1H), 7.68 (d, *J* = 15.5 Hz, 1H), 7.63 (d, *J* = 3.9 Hz, 1H), 7.56 (d, *J* = 3.9 Hz, 1H), 7.07 (d, *J* = 8.0 Hz, 2H), 6.65 (d, *J* = 8.0 Hz, 2H), 4.89 (d, *J* = 7.4 Hz, 2H), 4.59 – 4.51 (m, 1H), 3.63 (s, 3H), 3.04 (dd, *J* = 14.0, 5.3 Hz, 1H), 2.94 (dd, *J* = 14.0, 10.1 Hz, 1H), 1.45 (t, *J* = 7.4 Hz, 3H). ^{13}C NMR (126 MHz, DMSO) δ 172.3, 170.7, 160.8, 156.1, 143.0, 141.0, 139.8, 139.6, 139.0, 137.1, 130.2, 129.8, 128.6, 128.4, 127.6, 127.4, 126.9, 124.5, 116.7, 115.3, 111.7, 54.8, 52.3, 44.6, 35.7, 14.3. HRMS (ESI): m/z calcd for $C_{30}H_{27}N_2O_4S_3^+$ (M+): 575.1127 Found: 575.1115

Br-thiophene-Val-OMe

5-bromothiophene-2-carboxylic acid (100 mg, 0.483 mmol) together with H-Val-OMe·HCl (121 mg, 0.724 mmol) were set for general procedure of amide coupling, before purification using heptane/ethyl acetate as eluent gave Br-thiophene-Val-OMe (146 mg, 94%).

^{1}H NMR (500 MHz, MeOD) δ 7.63 (d, *J* = 4.0 Hz, 1H), 7.16 (d, *J* = 4.0 Hz, 1H), 4.41 (d, *J* = 7.1 Hz, 1H), 3.74 (s, 3H), 2.23 (h, *J* = 6.9 Hz, 1H), 1.00 (dd, *J* = 11.0, 6.8 Hz, 6H). ^{13}C NMR (126 MHz, MeOD) δ 173.5, 163.4, 141.5, 132.3, 130.7, 119.5, 60.1, 52.5, 31.7, 19.6, 19.1. LCMS (ESI): m/z calcd for $C_{11}H_{14}BrNO_3S$ (M+H): 320.0 Found: 322.2

Formyl-bi-thiophene-Val-OMe

Br-thiophene-Tyr-OMe (53.0 mg, 0.166 mmol) together with (5-formylthiophen-2-yl)boronic acid (27.1 mg, 0.174 mmol) were set for general procedure of Suzuki-Miyaura coupling, before purification by flash column chromatography using heptane/ethyl acetate as eluent gave the pure formyl-bi-thiophene-Val-OMe (35.0 mg, 60%).

^{1}H NMR (500 MHz, $CDCl_3$) δ 9.89 (s, 1H), 7.69 (d, *J* = 3.9 Hz, 1H), 7.49 (d, *J* = 3.9 Hz, 1H), 7.32 (d, *J* = 3.9 Hz, 1H), 7.30 (d, *J* = 3.9 Hz, 1H), 6.51 (d, *J* = 8.6 Hz, 1H), 4.74 (dd, *J* = 8.6, 4.9 Hz, 1H), 3.79 (s, 3H), 2.27 (pd, *J* = 6.9, 4.9 Hz, 1H), 1.00 (dd, *J* = 12.5, 6.9 Hz, 6H). ^{13}C NMR (126 MHz, $CDCl_3$) δ 182.7, 172.6, 161.1, 145.7, 143.1, 140.6, 139.3, 137.2, 129.1, 126.3, 125.7, 57.6, 52.5, 31.8, 19.1, 18.1. LCMS (ESI): m/z calcd for $C_{16}H_{17}NO_4S_2$ (M+H): 352.1 Found: 352.3

bTVBT-Val-OMe

Formyl-bi-thiophene-Val-OMe (35.0 mg, 0.0996 mmol) together with 3-ethyl-2-methylbenzothiazolium iodide (30.4 mg, 0.0996 mmol) were set for general procedure of condensation reaction, before crystallization from methanol gave bTVBT-Val-OMe (45.0 mg, 71%) as a dark red solid. HPLC purity: >98%

^{1}H NMR (500 MHz, $CDCl_3$) δ 8.44 (d, *J* = 15.0 Hz, 1H), 8.13 (d, *J* = 3.9 Hz, 1H), 8.11 (d, *J* = 8.1 Hz, 1H), 7.75 (d, *J* = 4.2 Hz, 1H), 7.72 (d, *J* = 11.0 Hz, 1H), 7.67 (t, *J* = 7.8 Hz, 1H), 7.57 (t, *J* = 7.6 Hz, 1H), 7.45 (d, *J* = 3.8 Hz, 1H), 7.21 (d, *J* = 3.9 Hz, 1H), 7.07 (d, *J* = 3.8 Hz, 1H), 6.71 (d, *J* = 8.5 Hz, 1H), 5.11 (d, *J* = 7.6 Hz, 2H), 4.70 (dd, *J* = 8.5, 5.2 Hz, 1H), 3.82 (s, 3H), 2.30 (td, *J* = 7.1, 5.4 Hz, 1H), 1.59 (t, *J* = 7.0 Hz, 3H), 1.03 (t, *J* = 6.6 Hz, 6H).

^{13}C NMR (126 MHz, $CDCl_3$) δ 173.1, 170.7, 162.4, 145.7, 142.8, 140.8, 139.9, 139.0, 138.2, 130.7, 130.2, 129.5, 128.4, 127.3, 127.1, 124.5, 116.3, 109.8, 58.8, 52.5, 45.4, 31.4, 19.3, 18.6, 14.5. HRMS (ESI): m/z calcd for $C_{26}H_{27}N_2O_3S_3^+$ (M+): 511.1178 Found: 511.1164

Br-thiophene-Gly-OMe

5-bromothiophene-2-carboxylic acid (100 mg, 0.483 mmol) together with H-Gly-OMe·HCl (91.0 mg, 0.724 mmol) were set for general procedure of amide coupling, before purification using dichloromethane/methanol as eluent gave Br-thiophene-Gly-OMe (114 mg, 85%) as a white oily solid.

^{1}H NMR (500 MHz, $CDCl_3$) δ 7.29 (d, *J* = 4.0 Hz, 1H), 7.04 (d, *J* = 4.0 Hz, 1H), 4.19 (d, *J* = 5.0 Hz, 2H), 3.80 (s, 3H). ^{13}C NMR (126 MHz, $CDCl_3$) δ 170.4, 160.9, 139.6, 130.9, 128.7, 118.7, 52.7, 41.7. LCMS (ESI): m/z calcd for $C_8H_8BrNO_3S$ (M+H): 277.9 Found: 278.1

Formyl-bi-thiophene-Gly-OMe

Br-thiophene-Gly-OMe (114 mg, 0.410 mmol) together with (5-formylthiophen-2-yl)boronic acid (67.1 mg, 0.430 mmol) were set for general procedure of Suzuki-Miyaura coupling, before purification by automated flash column chromatography using dichloromethane/methanol as eluent gave the pure formyl-bi-thiophene-Gly-OMe (27.0 mg, 21%).

^{1}H NMR (500 MHz, MeOD:$CDCl_3$) δ 9.84 (s, 1H), 7.79 (d, *J* = 4.0 Hz, 1H), 7.64 (d, *J* = 4.0 Hz, 1H), 7.39 (d, *J* = 4.0 Hz, 1H), 7.36 (d, *J* = 4.0 Hz, 1H), 4.10 (s, 2H), 3.75 (s, 3H). ^{13}C NMR (126 MHz, MeOD:$CDCl_3$) δ 184.2, 171.2, 163.3, 146.6, 143.3, 141.2, 139.8, 138.8, 130.4, 127.1, 126.4, 52.6, 41.9. LCMS (ESI): m/z calcd for $C_{13}H_{11}NO_4S_2$ (M+H): 310.0 Found: 310.2

bTVBT-Gly-OMe

Formyl-bi-thiophene-Gly-OMe (27.0 mg, 0.0873 mmol) together with 3-ethyl-2-methylbenzothiazolium iodide (26.6 mg, 0.0873 mmol) were set for general procedure of condensation reaction, before crystallization from methanol gave bTVBT-Gly-OMe (35.6 mg, 68%) as a dark red solid. HPLC purity: >99%

^{1}H NMR (500 MHz, DMSO) δ 9.11 (t, *J* = 5.9 Hz, 1H), 8.35 (d, *J* = 15.3 Hz, 1H), 8.28 (d, *J* = 8.1 Hz, 1H), 8.18 (d, *J* = 8.5 Hz, 1H), 7.91 – 7.80 (m, 2H), 7.78 – 7.68 (m, 2H), 7.67 – 7.57 (m, 2H), 7.54 (d, *J* = 4.0 Hz, 1H), 4.84 (q, *J* = 7.2 Hz, 2H), 3.98 (d, J = 5.9 Hz, 2H), 3.64 (s, 3H), 1.45 (t, *J* = 7.2 Hz, 3H). ^{13}C NMR (126 MHz, DMSO) δ 170.8, 161.8, 143.3, 141.3, 140.3, 139.7, 139.4, 137.4, 130.5, 130.2, 129.0, 128.6, 127.7, 127.4, 124.6, 116.9, 111.8, 52.5, 45.0, 41.6, 14.6. HRMS (ESI): m/z calcd for $C_{23}H_{21}N_2O_3S_3^+$ (M+): 469.0709 Found: 469.0710

Br-thiophene-Ala-OMe

5-bromothiophene-2-carboxylic acid (100 mg, 0.483 mmol) together with H-Ala-OMe·HCl (101 mg, 0.724 mmol) were set for general procedure of amide coupling, before purification using heptane/ethyl acetate as eluent gave Br-thiophene-Ala-OMe (96.0 mg, 68%).

^{1}H NMR (500 MHz, $CDCl_3$) δ 7.26 (d, *J* = 4.0 Hz, 1H), 7.00 (d, *J* = 4.0 Hz, 1H), 6.73 (d, *J* = 7.4 Hz, 1H), 4.72 (p, *J* = 7.3 Hz, 1H), 3.77 (s, 3H), 1.48 (d, *J* = 7.2 Hz, 3H). ^{13}C NMR (126 MHz, $CDCl_3$) δ 173.7, 160.4, 140.0, 130.8, 128.5, 118.6, 52.8, 48.5, 18.5. LCMS (ESI): m/z calcd for $C_9H_{10}BrNO_3S$ (M+H): 292.0 Found: 292.1

Formyl-bi-thiophene-Ala-OMe

Br-thiophene-Ala-OMe (96.0 mg, 0.329 mmol) together with (5-formylthiophen-2-yl)boronic acid (53.8 mg, 0.345 mmol) were set for general procedure of Suzuki-Miyaura coupling, before

purification by automated flash column chromatography using heptane/ethyl acetate as eluent gave the pure formyl-bi-thiophene-Ala-OMe (80.0 mg, 75%).

^{1}H NMR (500 MHz, $CDCl_3$) δ 9.87 (s, 1H), 7.68 (d, J = 3.9 Hz, 1H), 7.46 (d, J = 4.0 Hz, 1H), 7.30 (d, J = 4.0 Hz, 1H), 7.27 (d, J = 3.9 Hz, 1H), 6.76 (d, J = 7.4 Hz, 1H), 4.77 (p, J = 7.3 Hz, 1H), 3.80 (s, 3H), 1.52 (d, J = 7.2 Hz, 3H). ^{13}C NMR (126 MHz, $CDCl_3$) δ 182.7, 173.7, 160.7, 145.7, 143.0, 140.6, 139.3, 137.2, 129.1, 126.3, 125.6, 52.9, 48.7, 18.7. LCMS (ESI): m/z calcd for $C_{14}H_{13}NO_4S_2$ (M+H): 324.0 Found: 324.2

bTVBT-Ala-OMe

Formyl-bi-thiophene-Ala-OMe (20.0 mg, 0.0619 mmol) together with 3-ethyl-2-methylbenzothiazolium iodide (18.9 mg, 0.0619 mmol) were set for general procedure of condensation reaction, before crystallization from methanol gave bTVBT-Ala-OMe (17.0 mg, 45%) as a dark red solid. HPLC purity: >97%

^{1}H NMR (500 MHz, $CDCl_3$:Acetone) δ ^{1}H NMR (500 MHz, Acetone) δ 8.41 (d, J = 15.3 Hz, 1H), 8.30 (dd, J = 8.0, 2.4 Hz, 1H), 8.17 (dd, J = 8.6, 2.3 Hz, 1H), 7.89 – 7.87 (m, 1H), 7.86 – 7.82 (m, 1H), 7.79 – 7.70 (m, 3H), 7.41 (dd, J = 4.0, 2.2 Hz, 1H), 7.36 (dd, J = 4.1, 2.2 Hz, 1H), 5.09 (q, J = 6.9 Hz, 2H), 4.63 (q, J = 7.3 Hz, 1H), 3.69 (s, 3H), 1.65 (t, J = 7.2 Hz, 3H), 1.46 (d, J = 7.3 Hz, 3H). ^{13}C NMR (126 MHz, $CDCl_3$:Acetone) δ 173.1, 171.0, 161.0, 145.1, 142.3, 138.9, 137.9, 130.3, 129.6, 129.1, 127.0, 126.6, 124.6, 116.4, 110.6, 52.2, 48.7, 45.4, 17.4, 14.5. HRMS (ESI): m/z calcd for $C_{24}H_{23}N_2O_3S_3^+$ (M+): 483.0865 Found: 483.0861

Optical characterization and solvatochromism

Stock solutions (1.5 mM) were prepared by dissolving bTVBT2, bTVBT-G-OMe, bTVBT-A-OMe, bTVBT-V-OMe, bTVBT-Y-OMe and bTVBT-E-diOMe in DMSO. For the optical characterization, the ligands were further diluted in phosphate buffered saline (PBS, 10 mM phosphate, 140 mM NaCl, 2.7 mM KCl, pH 7.4) to 30 μM or in methanol, ethanol, DMSO, acetonitrile and THF to 15 μM. The absorbance, excitation and emission spectrum of each ligand was recorded using a Tecan Infinite M1000 Pro plate reader (Tecan). When collecting the excitation spectra, the emission wavelength was 590 nm, and when collecting the emission spectra, the excitation wavelength was 470 nm or corresponding to the absorption maximum of the respective ligand. Quantium yield (QY) of the bTVBT based ligands was determined in PBS, DMSO or THF. The values were determined using the slope method by plotting the integrated fluorescence intensity vs. the absorbance at λ_{ex} for a series of low weakly absorbing samples to avoid inner filter effects (OD < 0.1).[37]

Ligand staining of AD brain tissue sections

The collection of postmortem human brain tissue and the experimental procedures in which the tissue was included were reviewed and approved by the Indiana University Institutional Review Board and the Swedish Ethical Review Authority (Dnr 2020-01197). Informed consent was obtained from the patient or their next of kin.

Frozen brain sections (10 µm thickness of parietal cortex) of an individual diagnosed with sporadic AD (case 1, **Table S2**) were fixed with 99,7 % EtOH for 10 min, incubated 10 min in 50 % EtOH followed by 10 min in dH_2O at RT and then 10 min in PBS. The sections were incubated with 10 µM of the respective ligand in PBS for 30 min at RT. The sections were then washed with PBS three times and mounted with Dako mounting medium for fluorescence (Agilent). The mounting medium was allowed to solidify at least overnight before sealing the cover slips with nail polish. The sections were analyzed using an inverted Zeiss LSM 780 laser scanning confocal microscope (Zeiss) using an excitation at 535 nm and the emission spectra were collected between 540 nm to 690 nm.

For the competition experiments with HS-339, the sections were stained as described followed by an incubation with 100 nM HS-339 after washing three times with PBS. For the dual-ligand staining experiment, the sections were stained with 100 nM of the respective bTVBT-based ligand together with 100 nM HS-276. The sections were then washed with PBS three times and mounted with Dako mounting medium for fluorescence (Agilent). The mounting medium was allowed to solidify at least overnight before sealing the cover slips with nail polish. The sections were analyzed using an inverted Zeiss LSM 780 laser scanning confocal microscope (Zeiss) using an excitation at 405 nm (HS-339) and 535 nm (bTVBT based ligands) and the emission spectra were collected between 440 nm to 690 nm. For the channel mode set up the following settings were used:

HS-339:	Excitation: 405 nm	Emission: 410-514 nm
HS-276:	Excitation: 405 nm	Emission: 410-514 nm
bTVBT based ligands:	Excitation: 535 nm	Emission: 540-647 nm

Ligand and antibody double staining of AD brain tissue sections

For the ligand and antibody double staining experiment, frozen brain sections from three individuals diagnosed with sporadic AD (Table S2) were used. The thickness of the sections was 10 µm and the brain regions used were parietal (case 1 and 2) and frontal cortex (case 3). The brain sections were fixed with pre-chilled 70% EtOH for 3 min at 4°C. The sections were then incubated in dH_2O for 2x2 min and in PBS for 10 min at RT before the blocking buffer composed of PBS with 0.1% triton X-100 (PBS-T) and 5% normal goat serum (Merck) was

added. After 1 h, the blocking buffer was removed, and the sections were incubated with mouse monoclonal anti-tau antibody GT-38 (Abcam, ab246808), diluted 1:1000 in blocking buffer, over night at 4°C. The sections were washed with PBS-T for 3x10 min and then incubated with goat anti-mouse secondary antibody conjugated with Alexa 488 (ThermoFisher), diluted 1:500 in blocking buffer, for 1 h at RT. After washing in PBS for 3x10 min, the sections were stained for 30 min at RT with 100 nM bTVBT2, bTVBT-G-OMe, bTVBT-A-OMe, bTVBT-V-OMe, bTVBT-Y-OMe or bTVBT-E-diOMe diluted in PBS. The sections were then washed with PBS 3 times and mounted using Dako mounting medium for fluorescence (Agilent). The staining result was analysed using a Zeiss 780 confocal laser scanning microscope (Zeiss).

^{1}H NMR Br-thiophene-Glu-diOMe

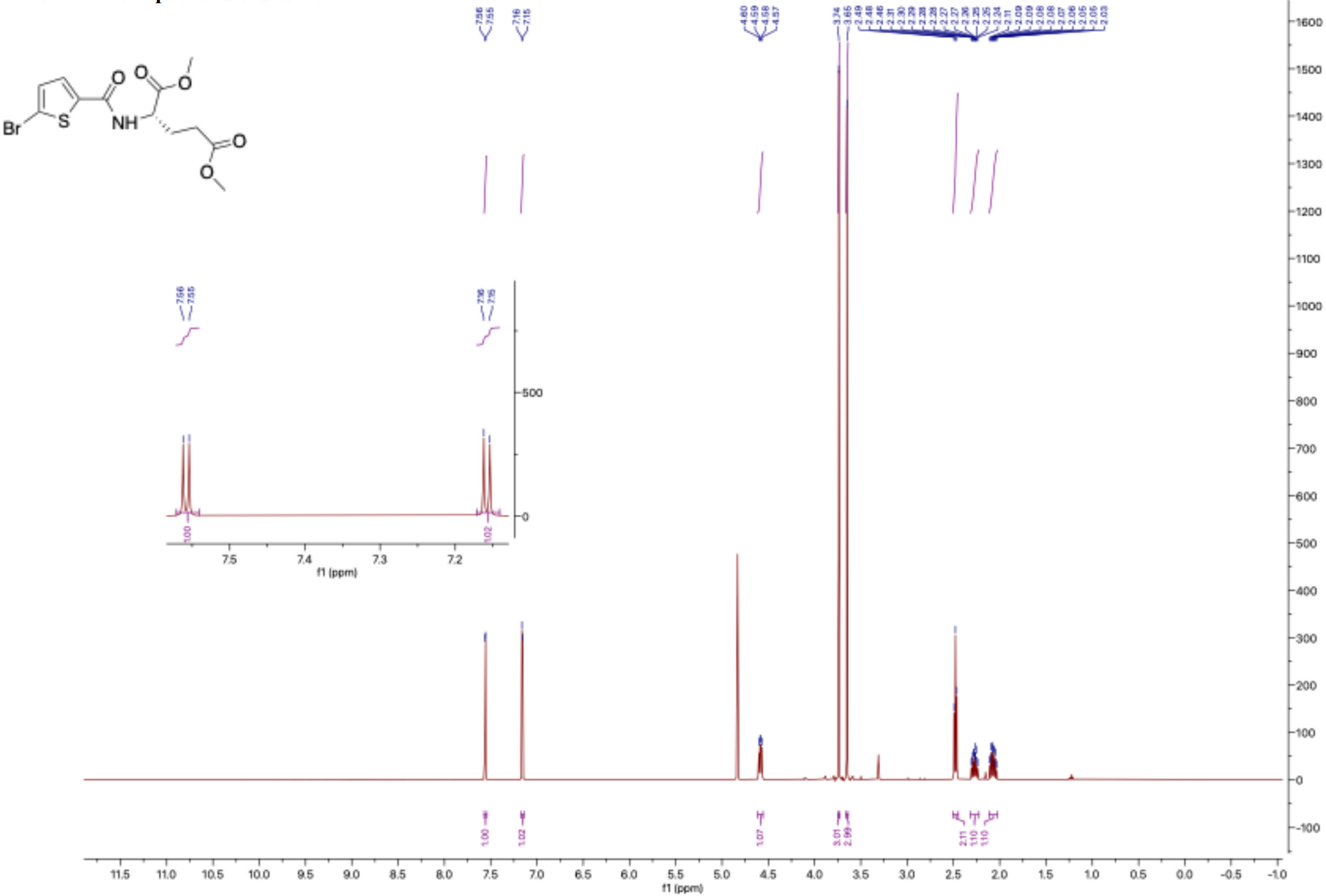

^{13}C NMR Br-thiophene-Glu-diOMe

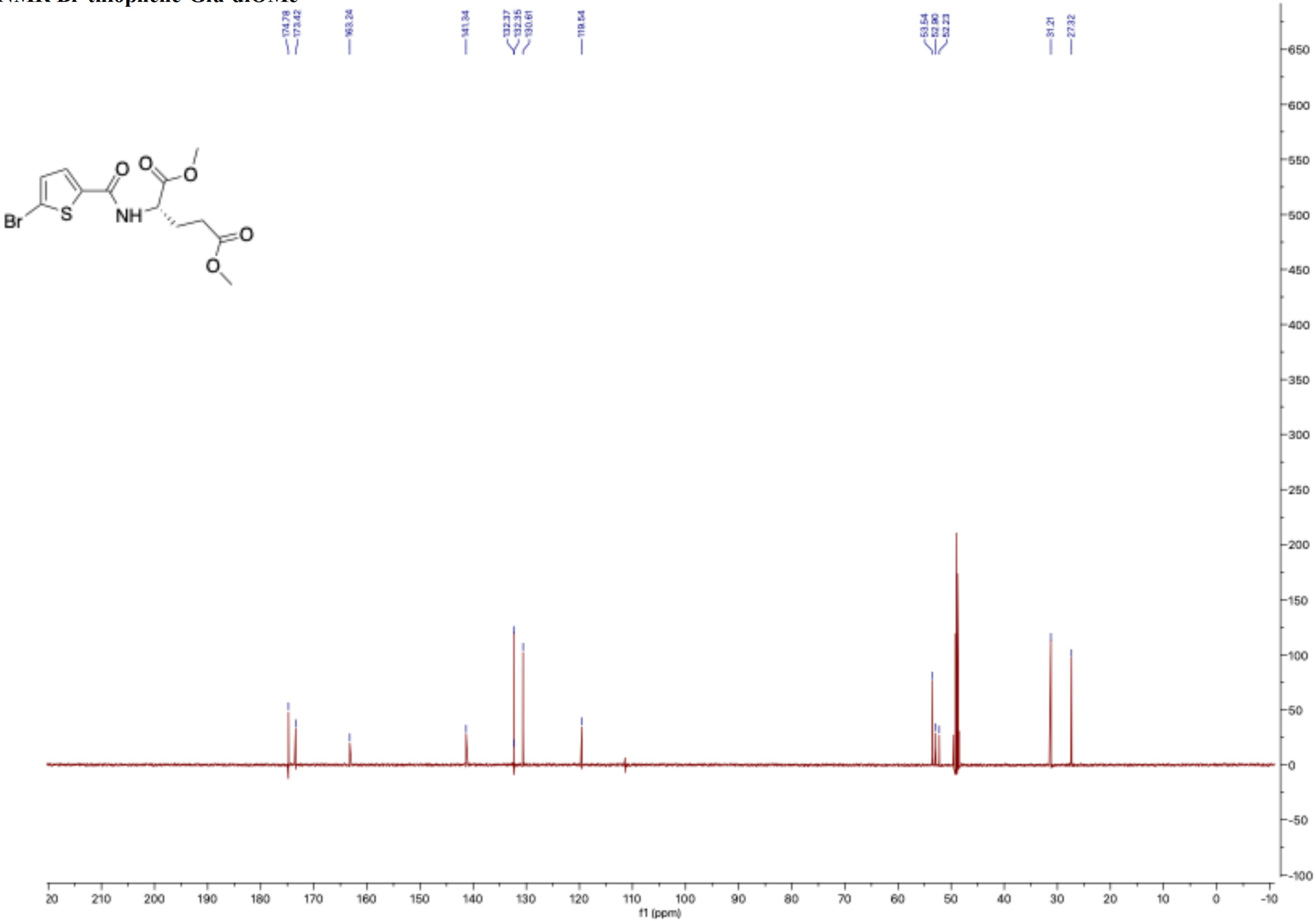

^{1}H NMR Formyl-bi-thiophene-Glu-diOMe

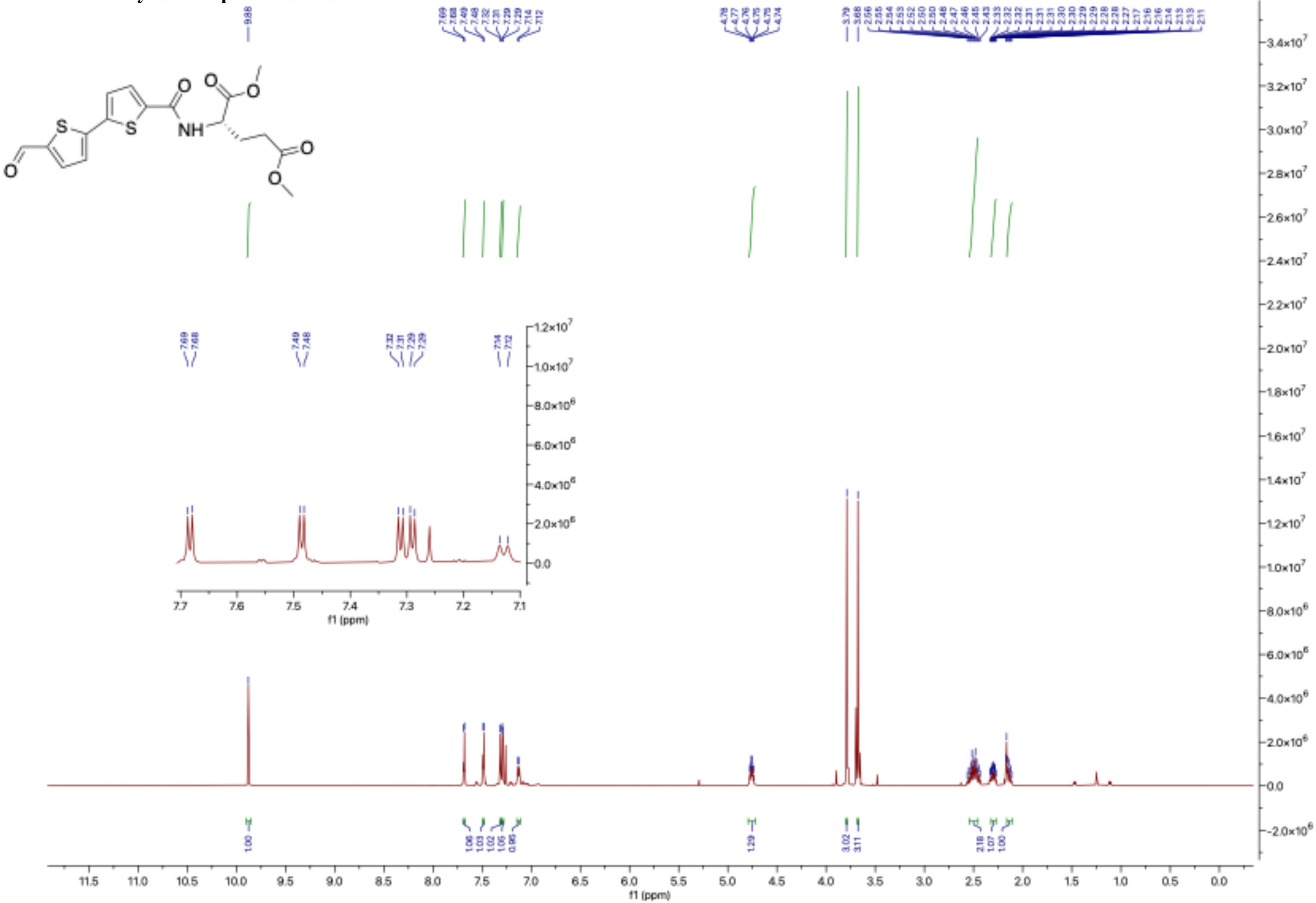

^{13}C NMR Formyl-bi-thiophene-Glu-diOMe

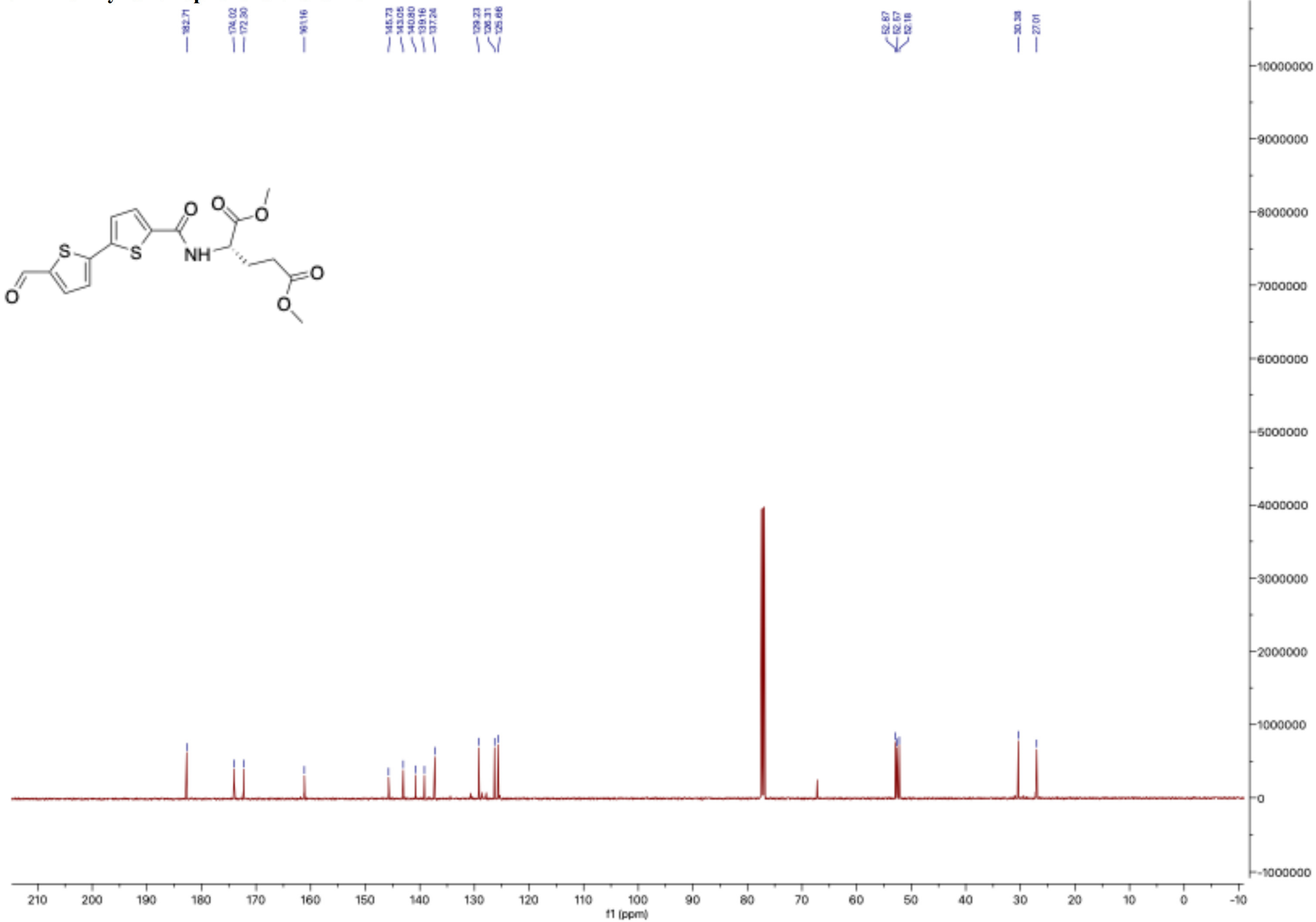

^1H NMR bTVBT-Glu-diOMe

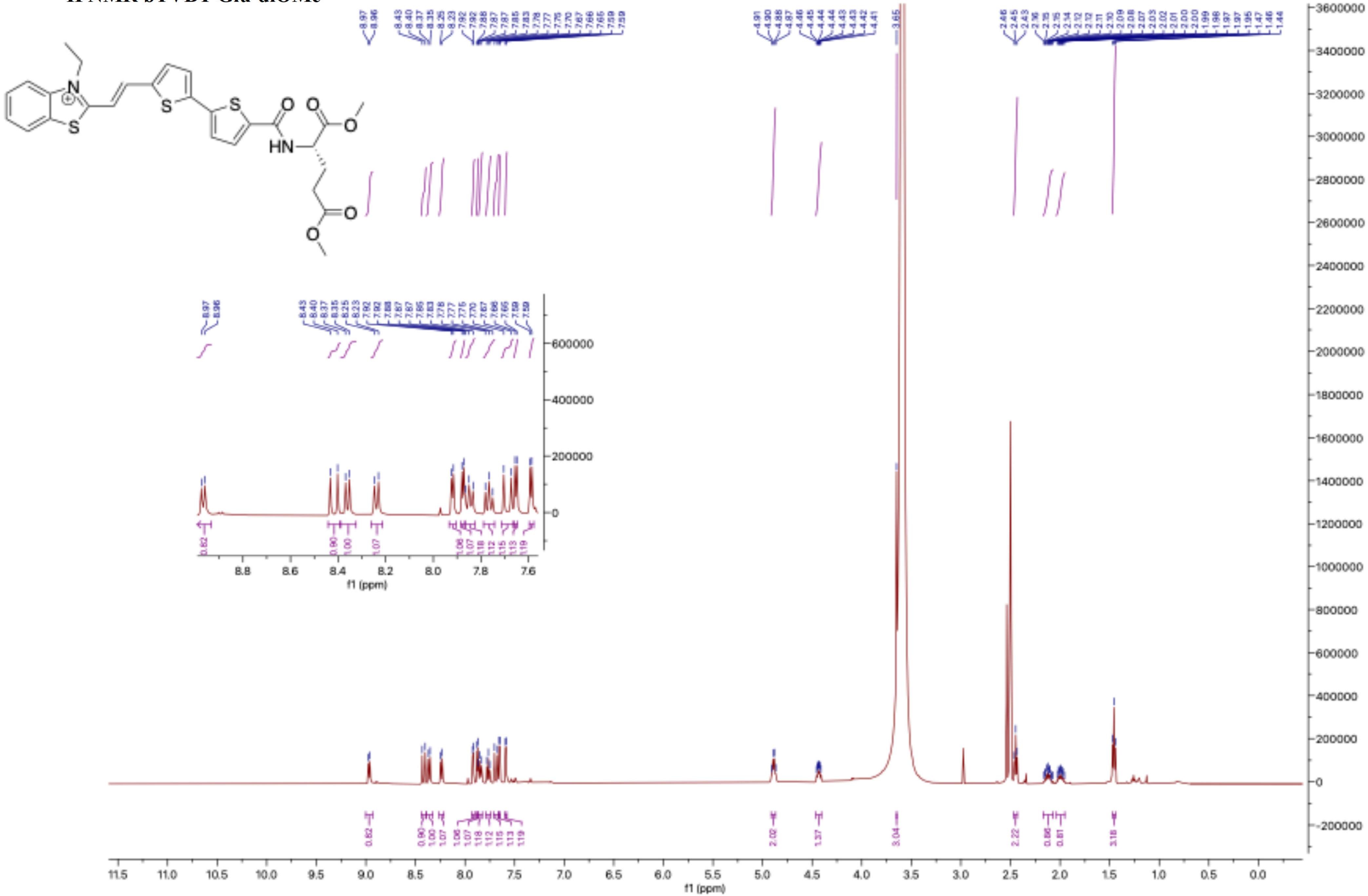

^{13}C NMR bTVBT-Glu-diOMe

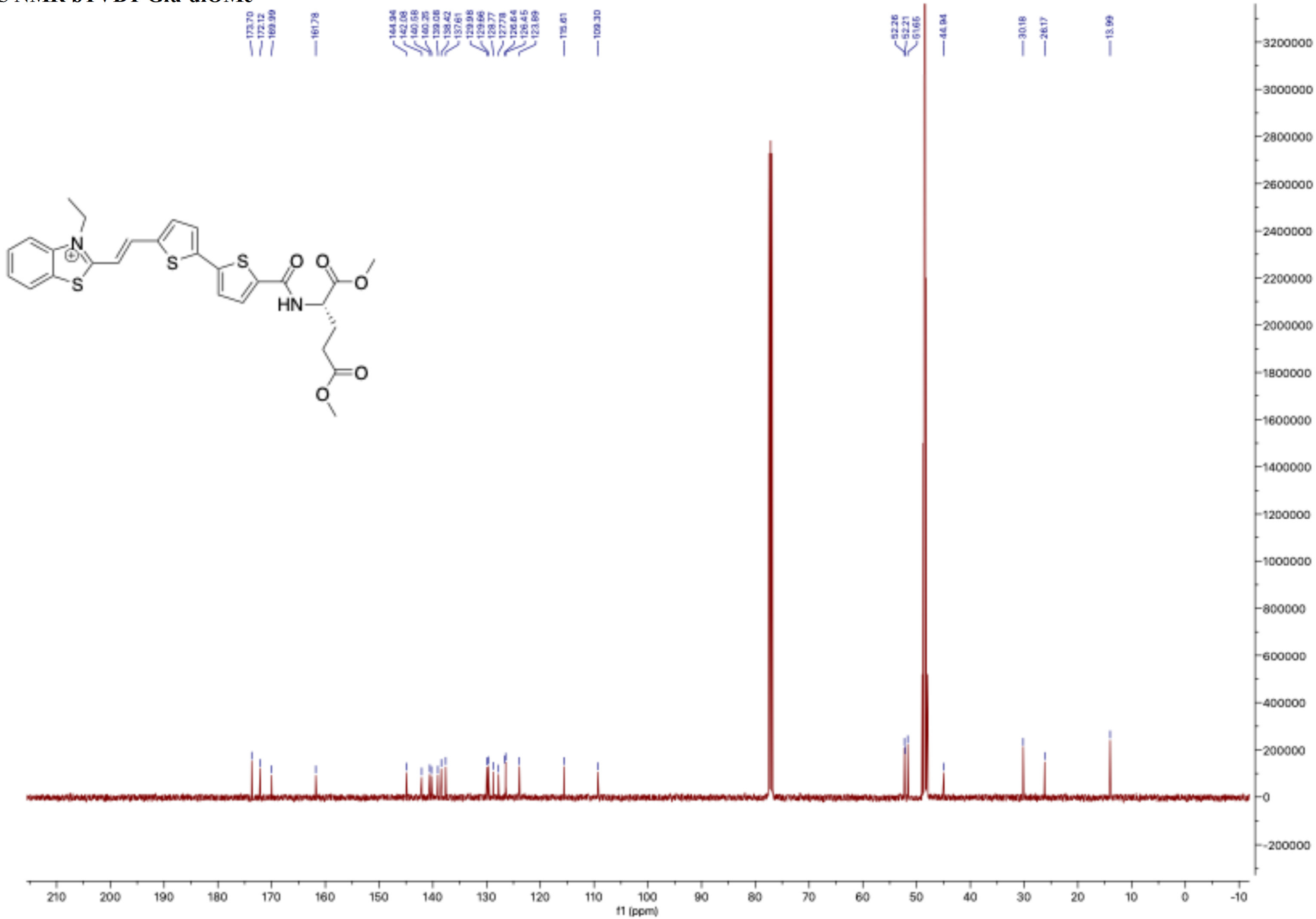

^{1}H NMR Br-thiophene-Tyr-OMe

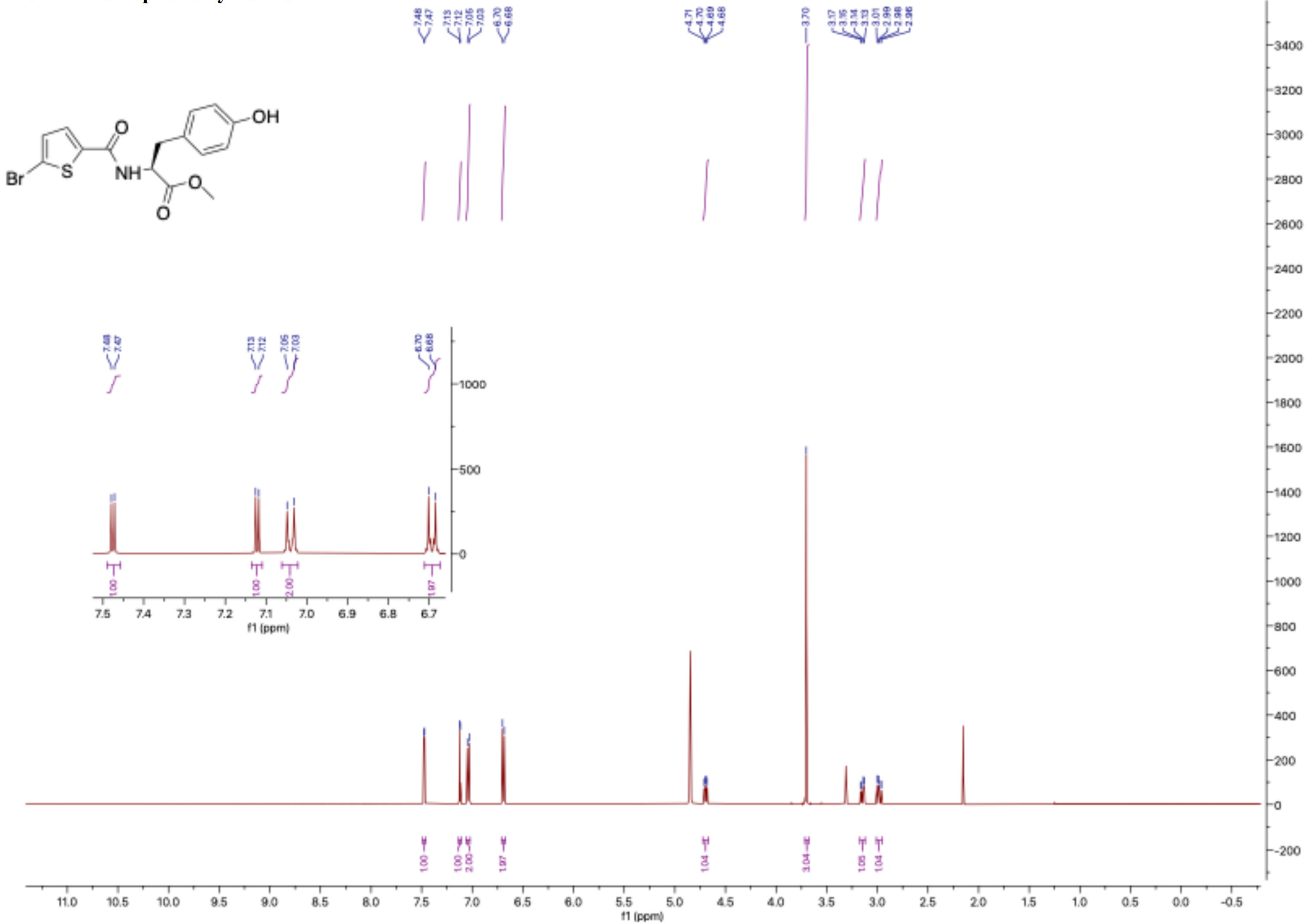

^{13}C NMR Br-thiophene-Tyr-OMe

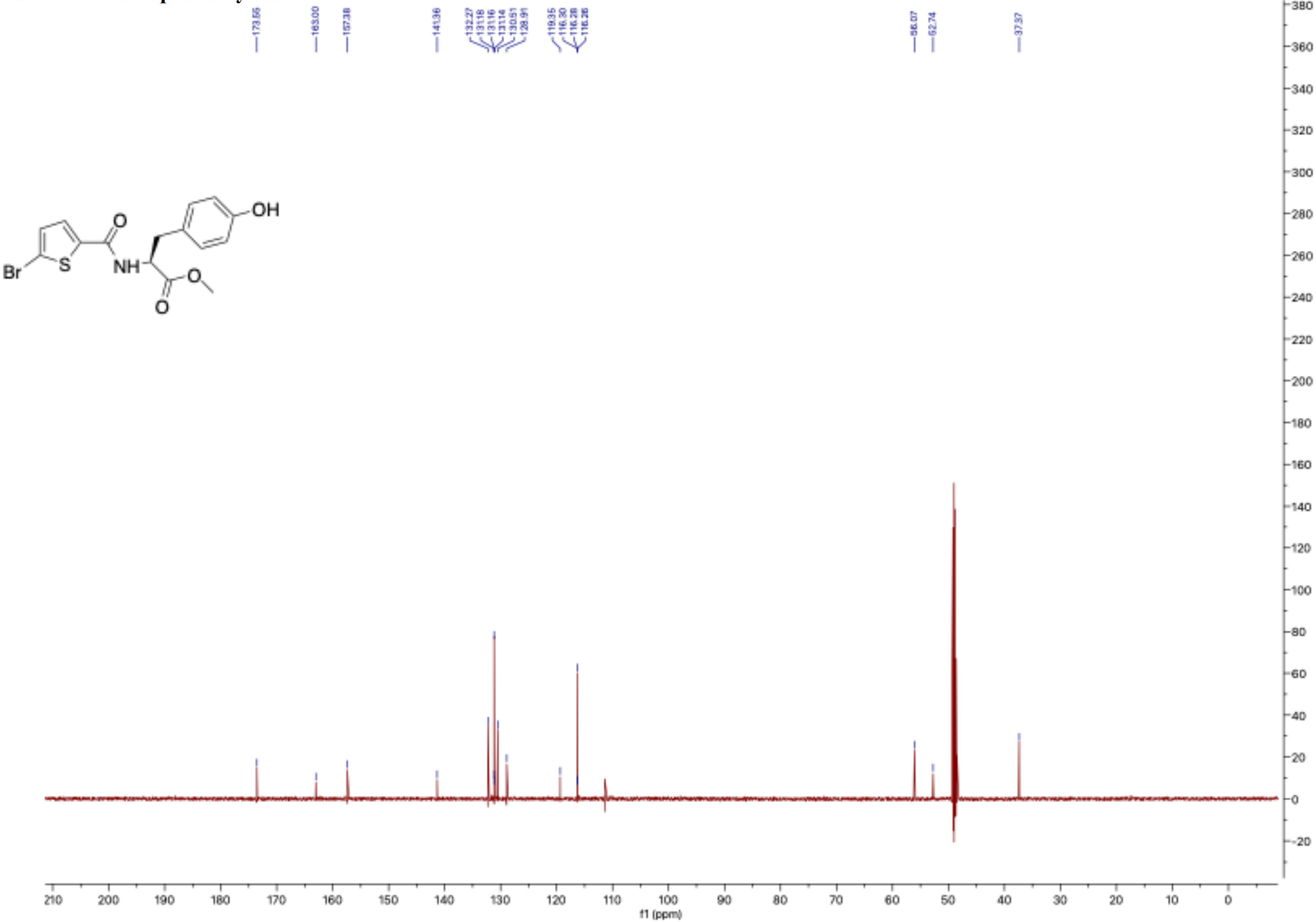

^{1}H NMR Formyl-bi-thiophene-Tyr-OMe

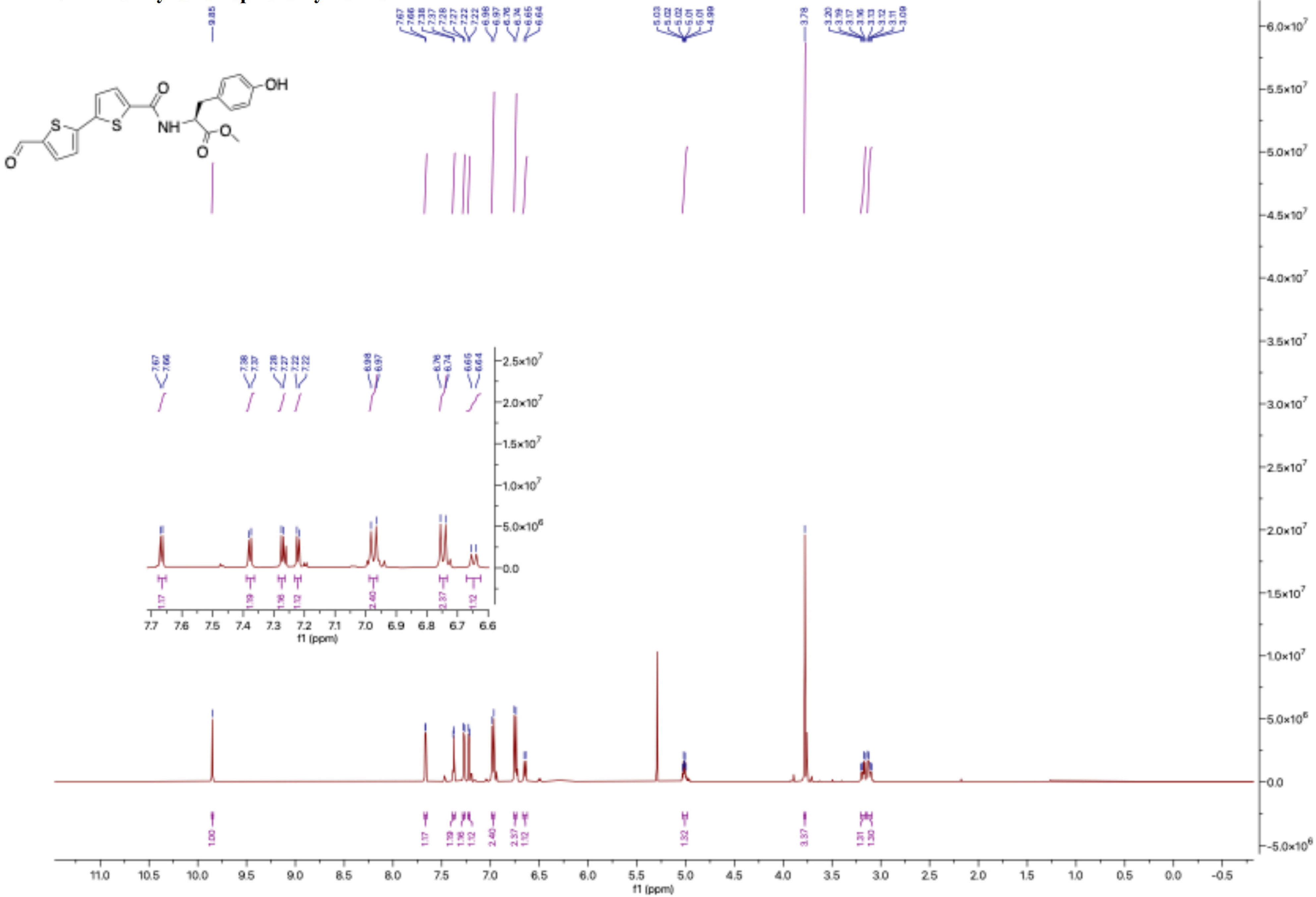

^{13}C NMR Formyl-bi-thiophene-Tyr-OMe

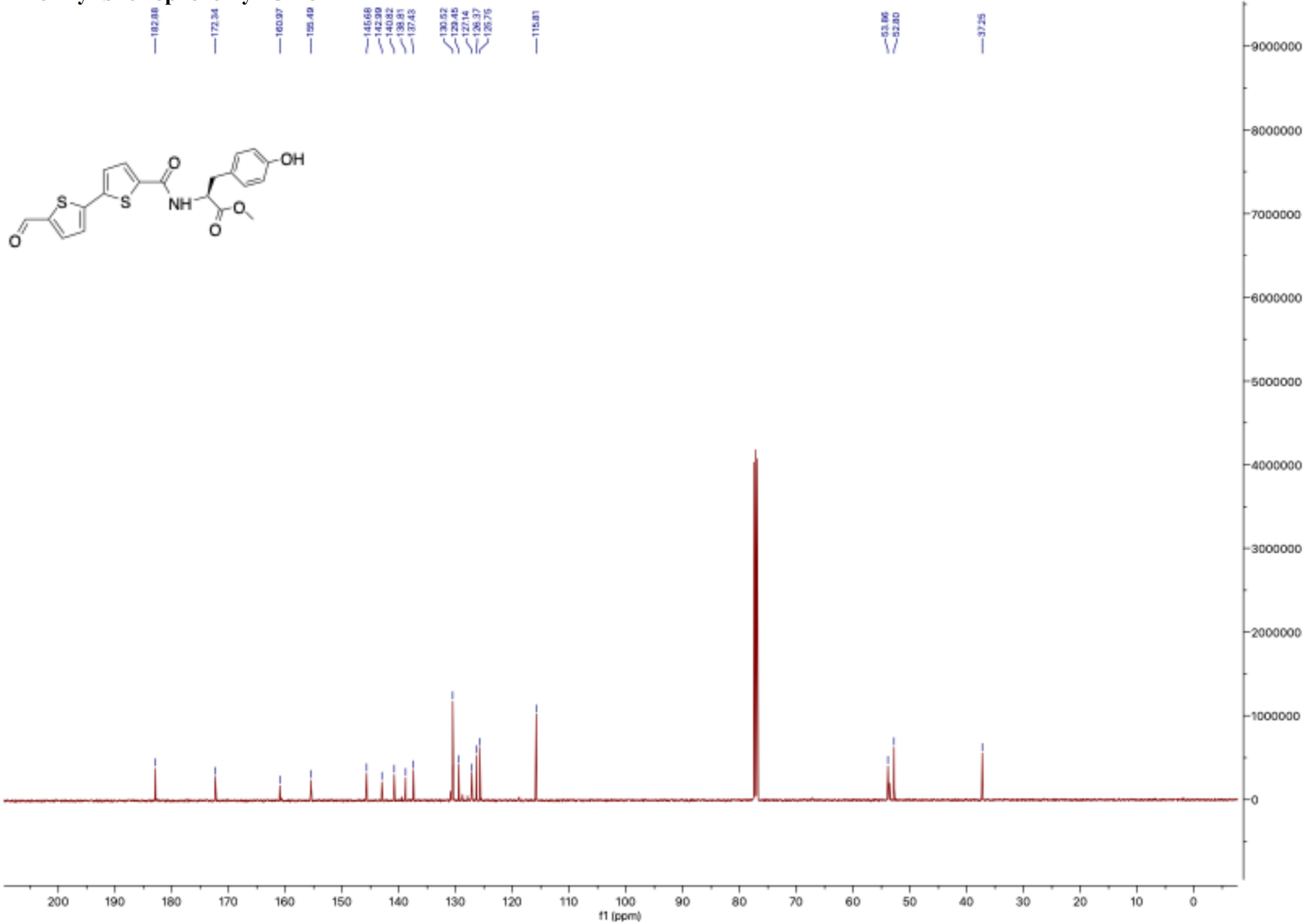

^{1}H NMR bTVBT-Tyr-OMe

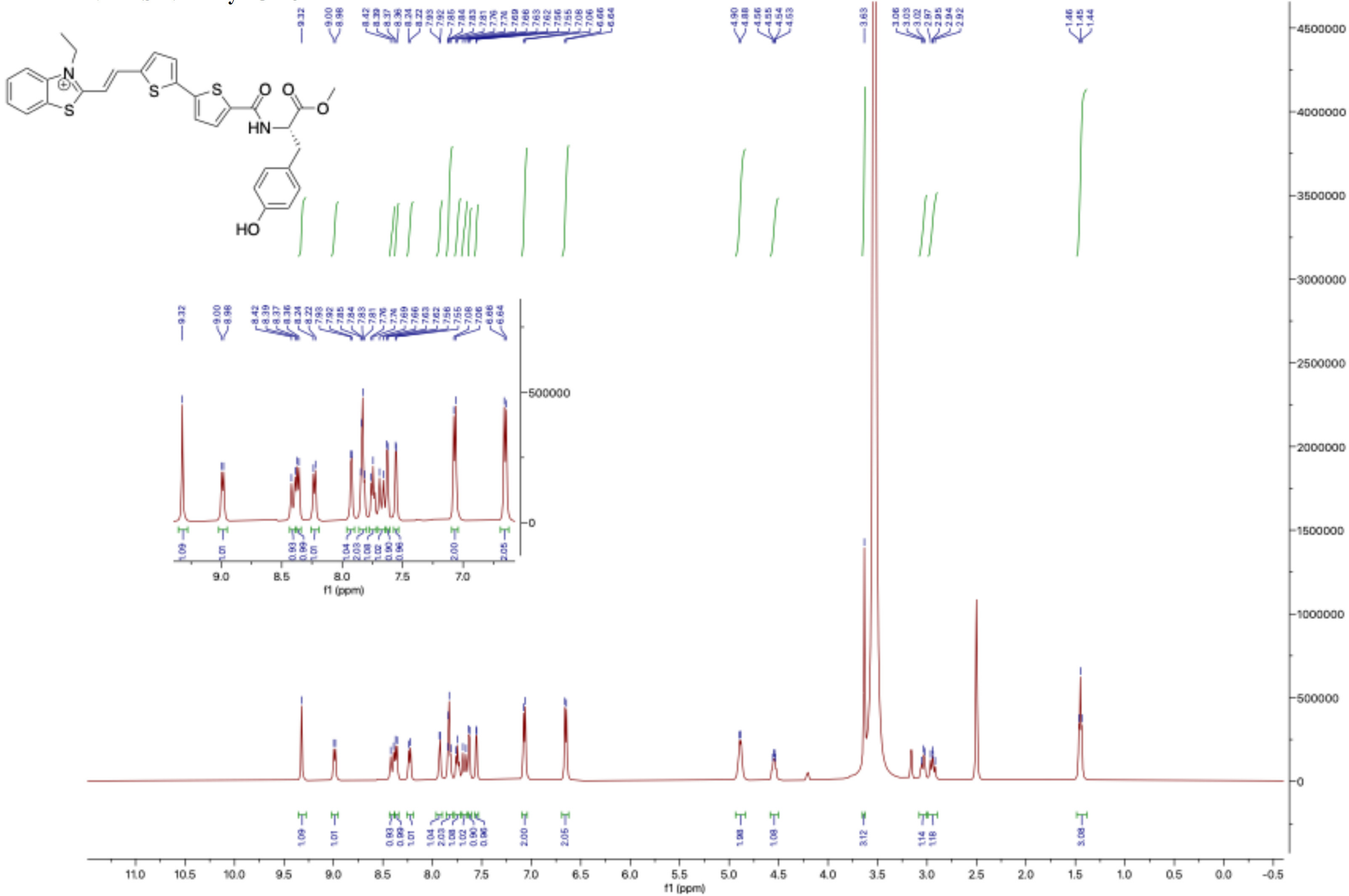

^{13}C NMR bTVBT-Tyr-OMe

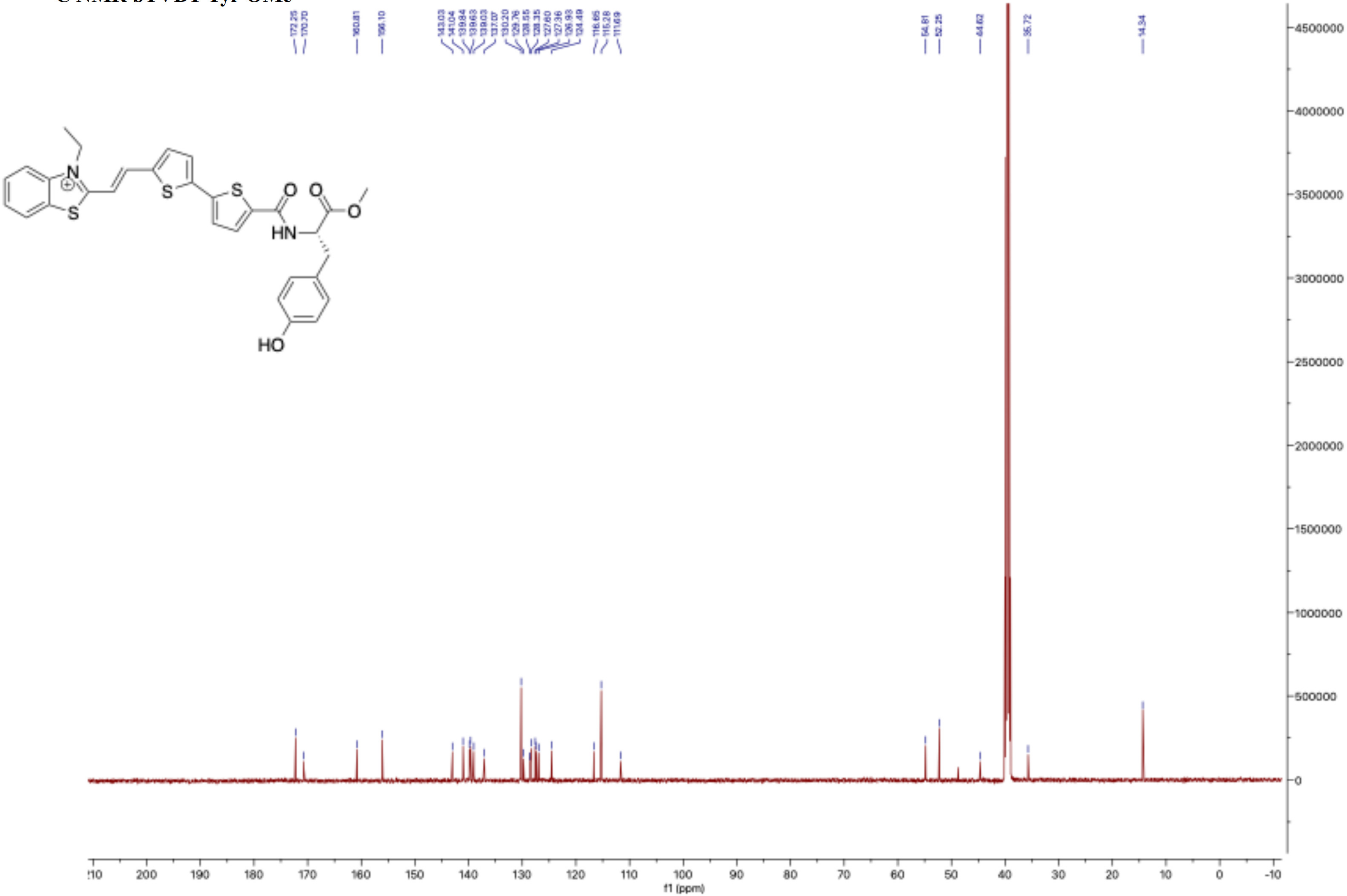

^{1}H NMR Br-thiophene-Val-OMe

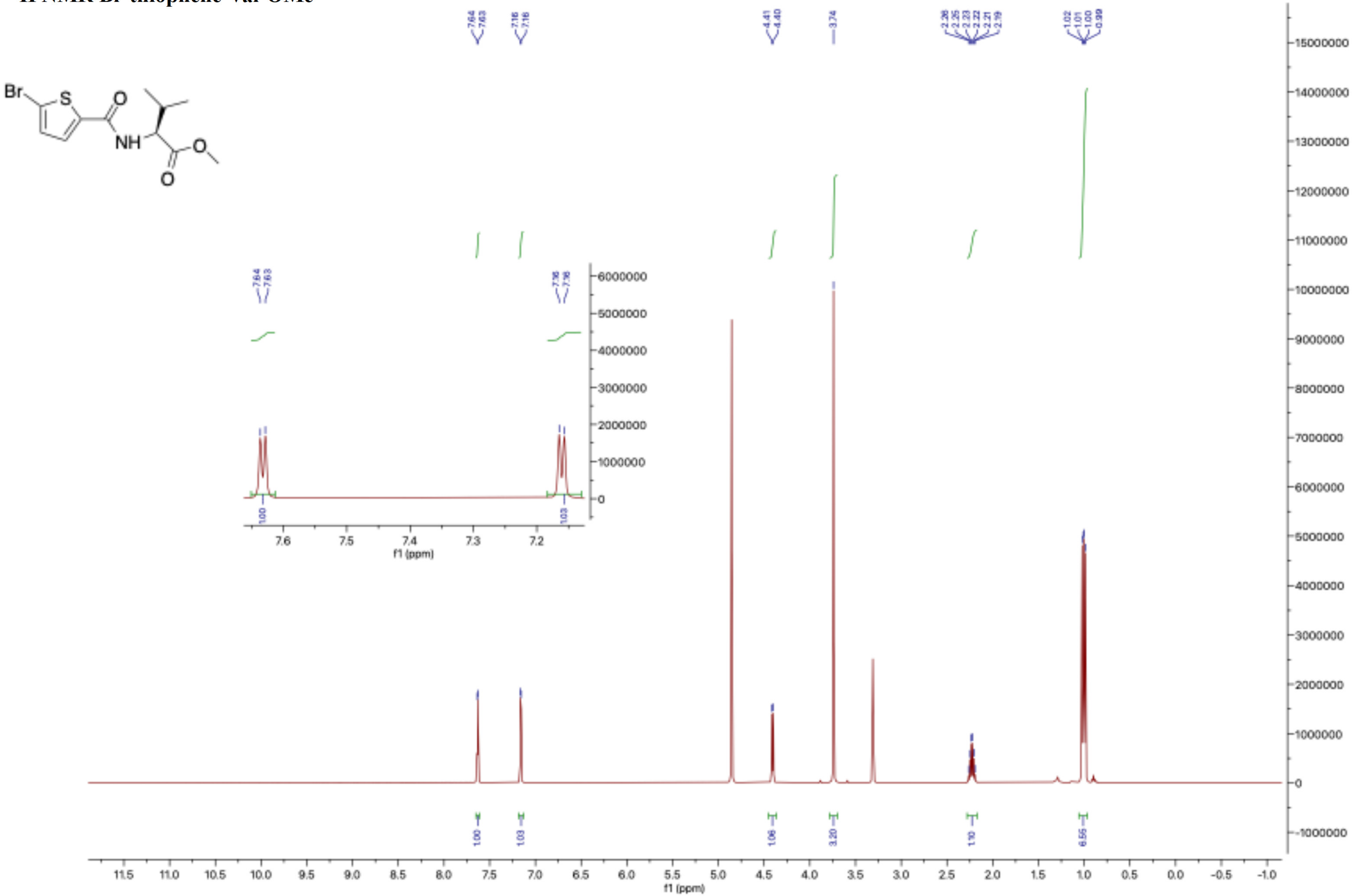

^{13}C NMR Br-thiophene-Val-OMe

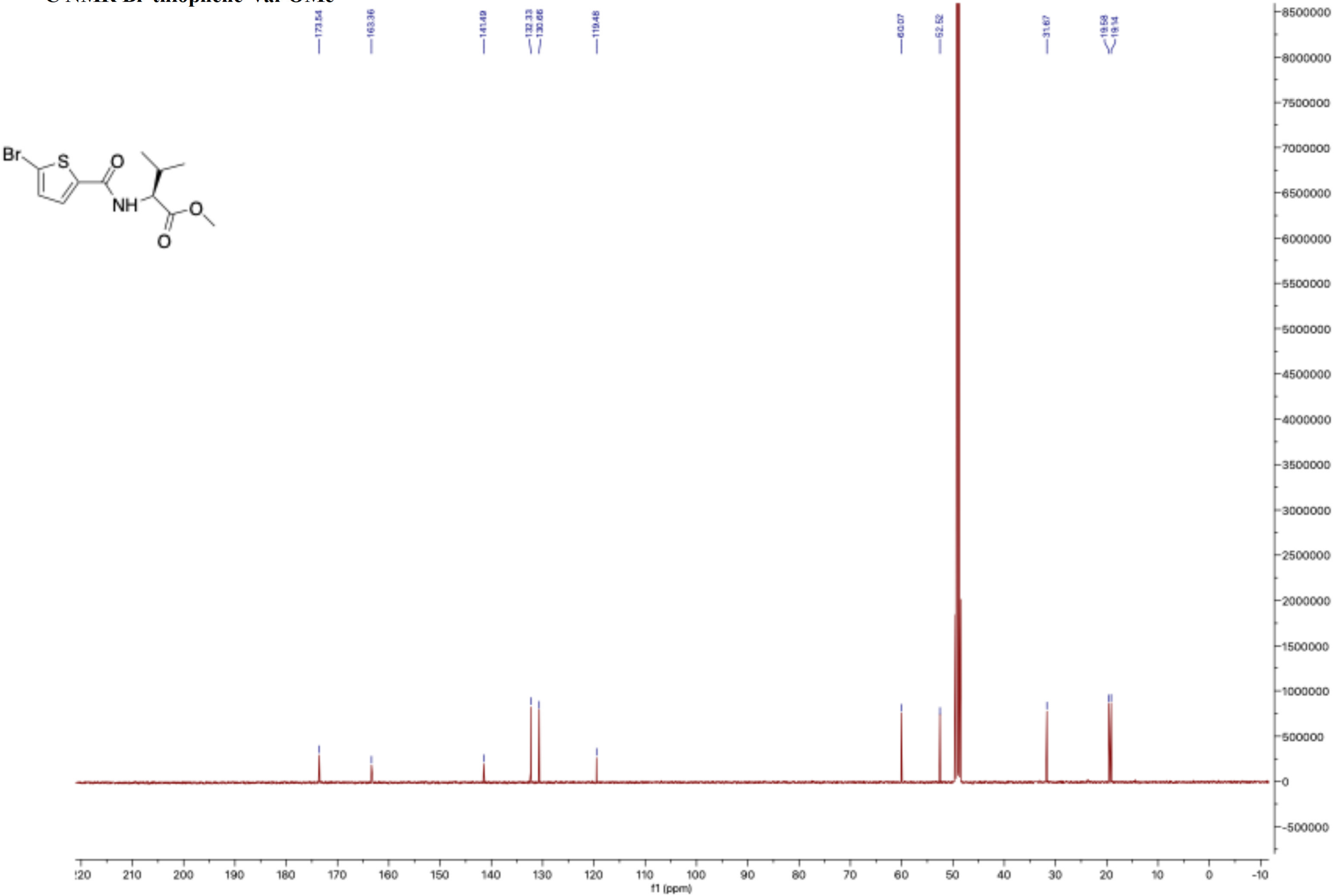

^{1}H NMR Formyl-bi-thiophene-Val-OMe

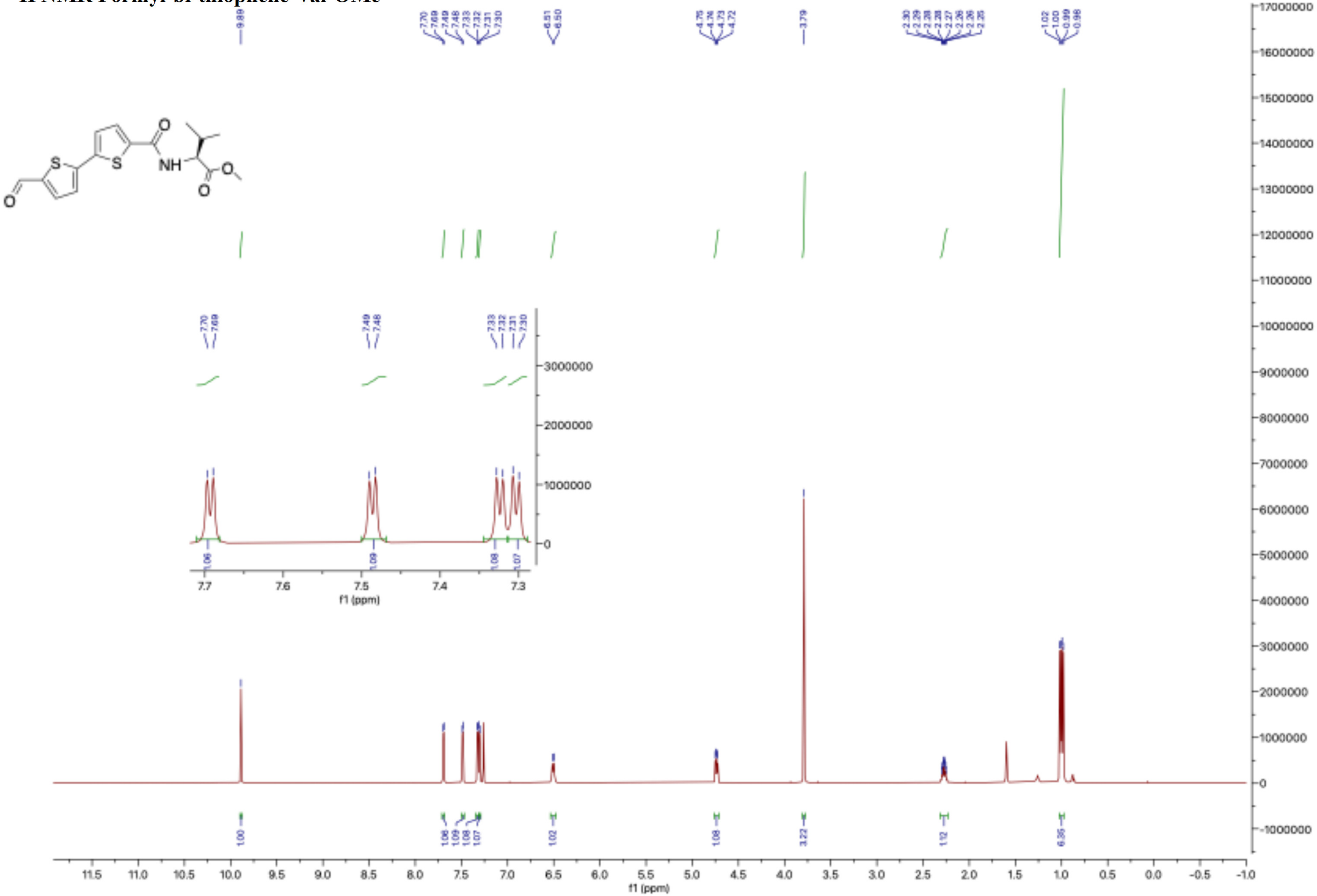

^{13}C NMR Formyl-bi-thiophene-Val-OMe

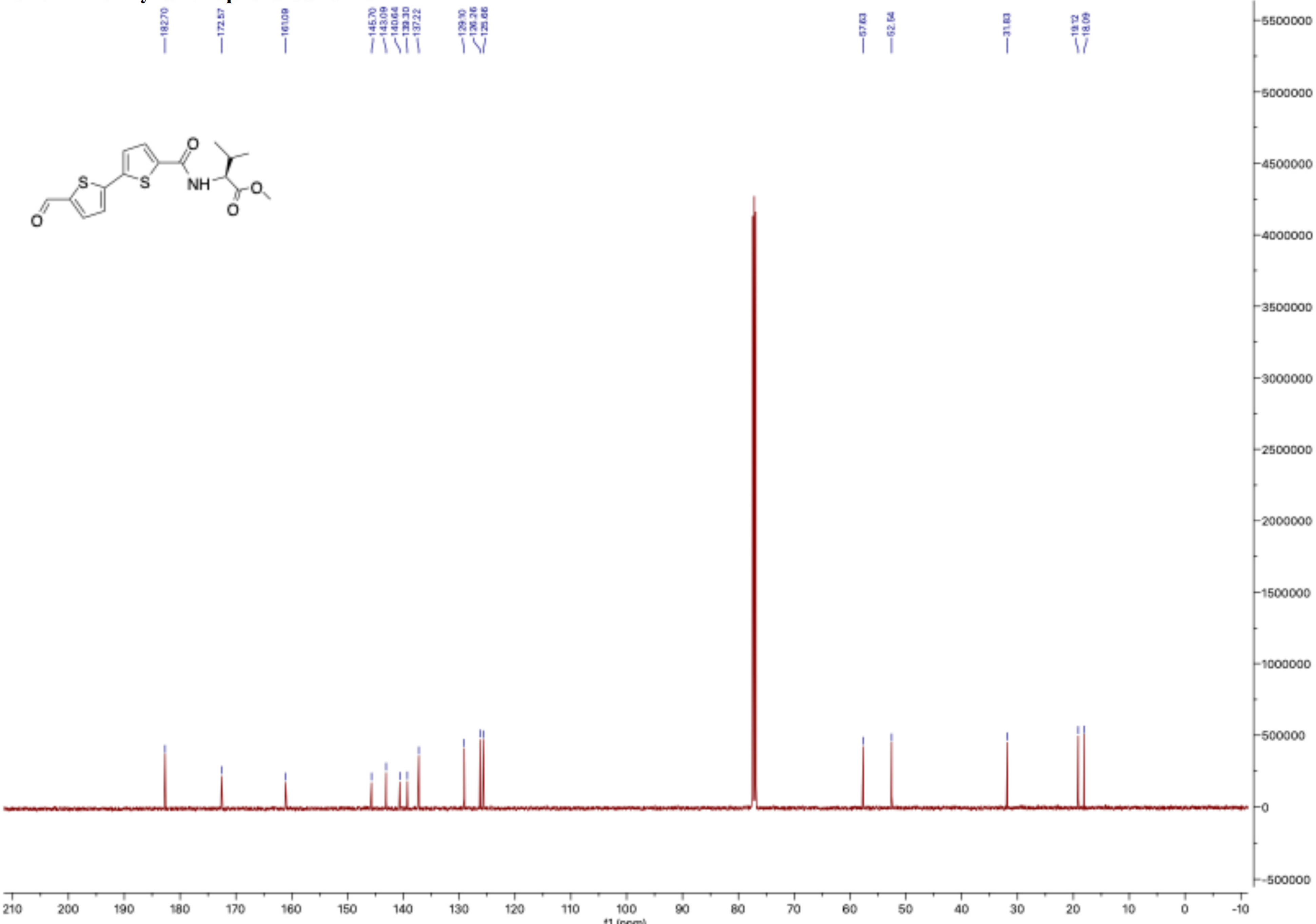

^{1}H NMR bTVBT-Val-OMe

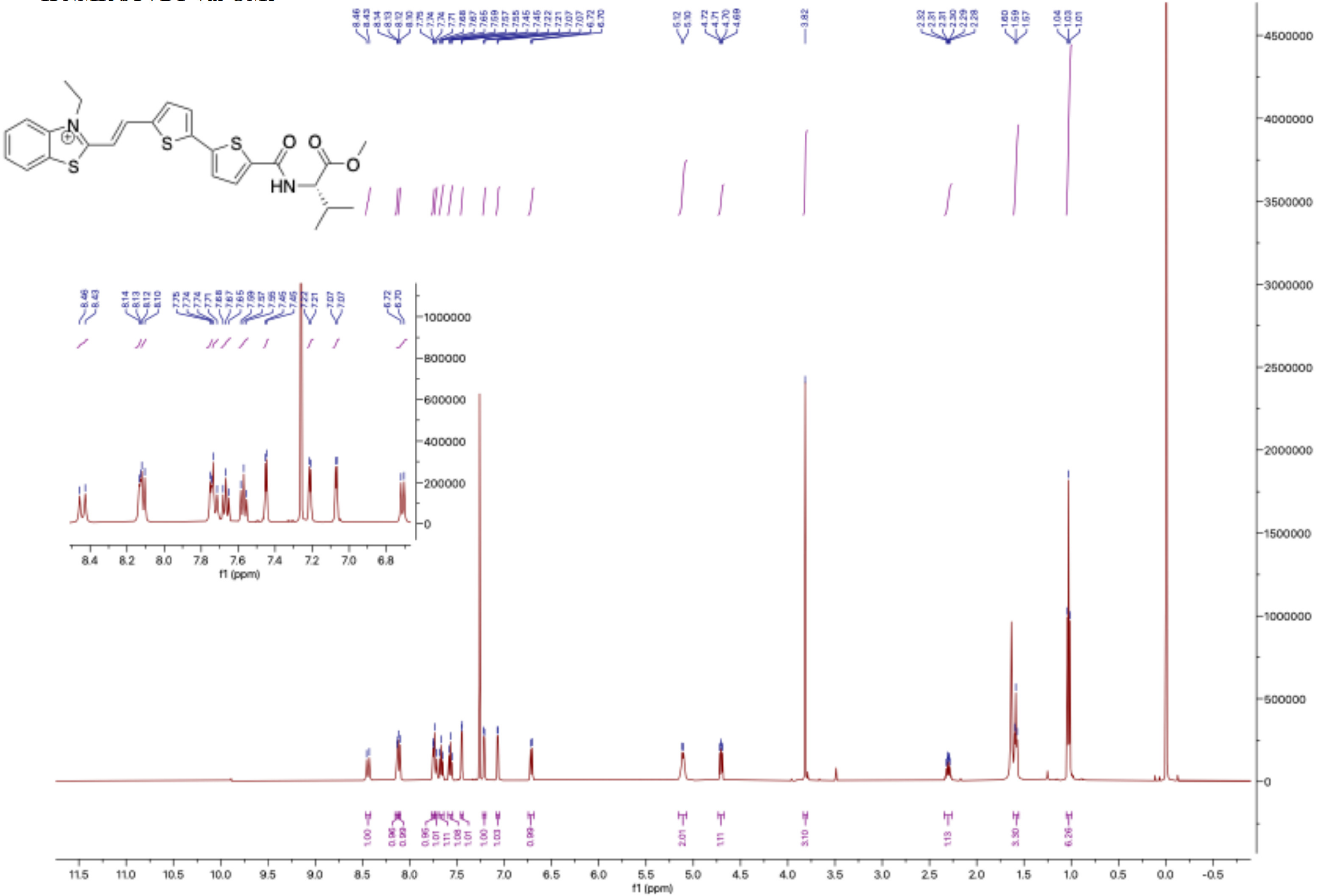

^{13}C NMR bTVBT-Val-OMe

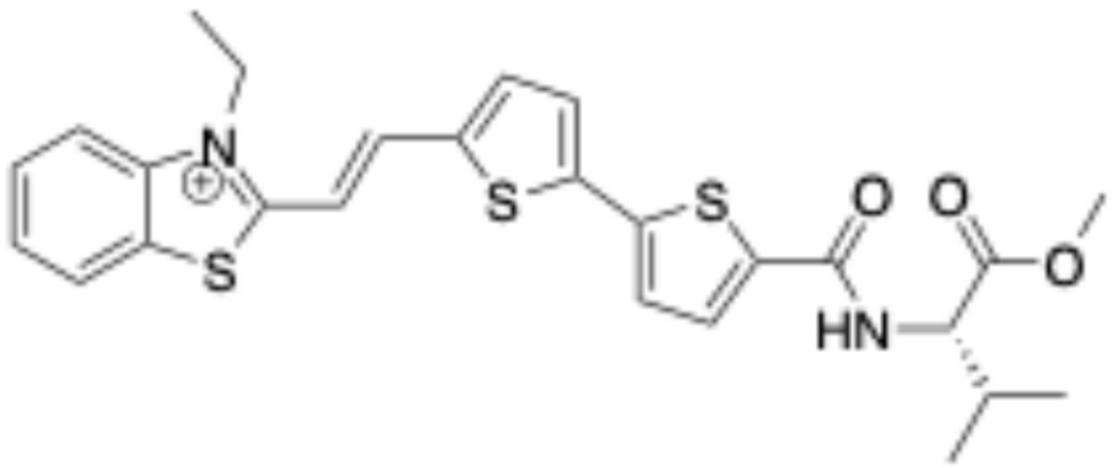

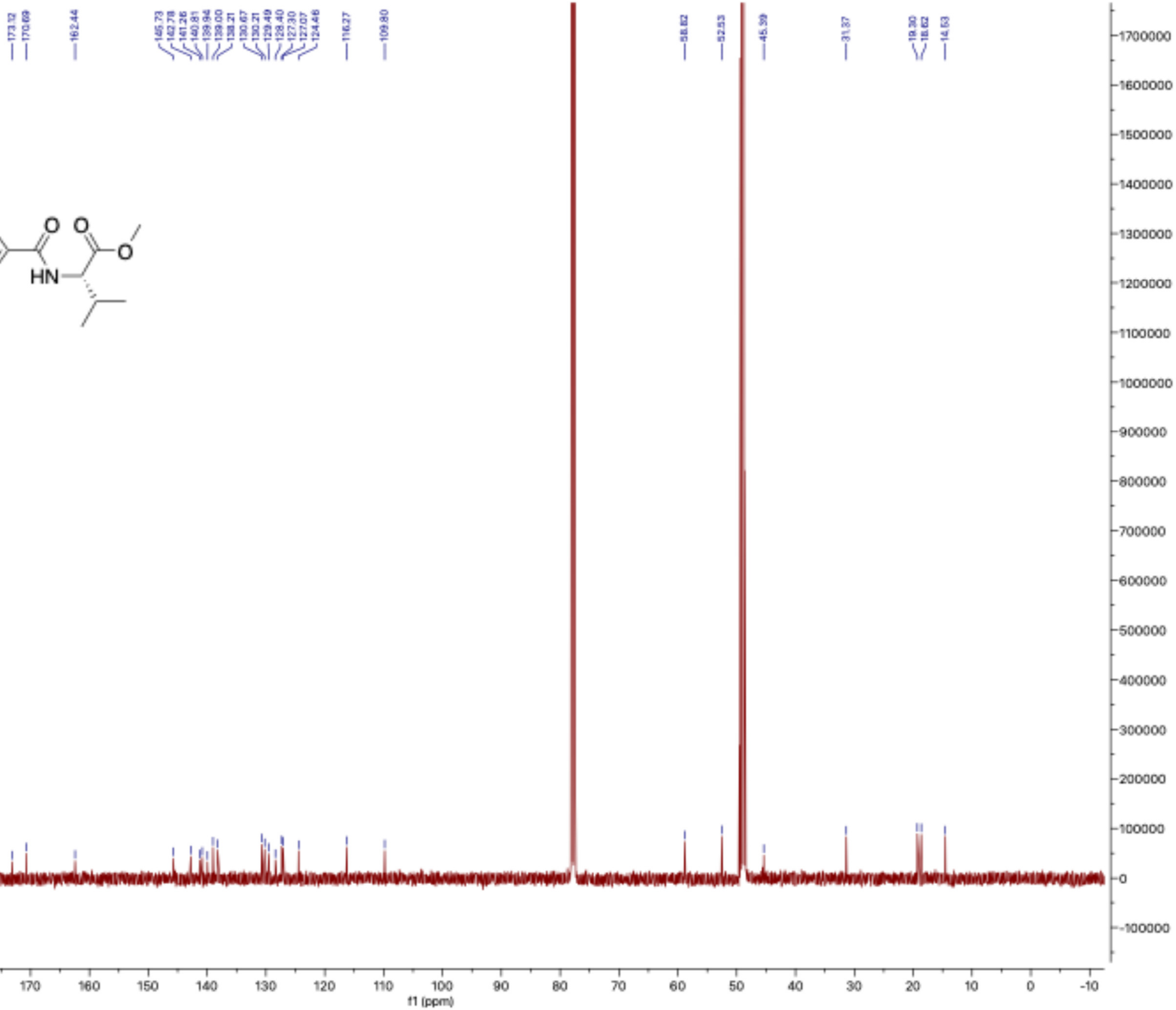

^{1}H NMR Br-thiophene-Gly-OMe

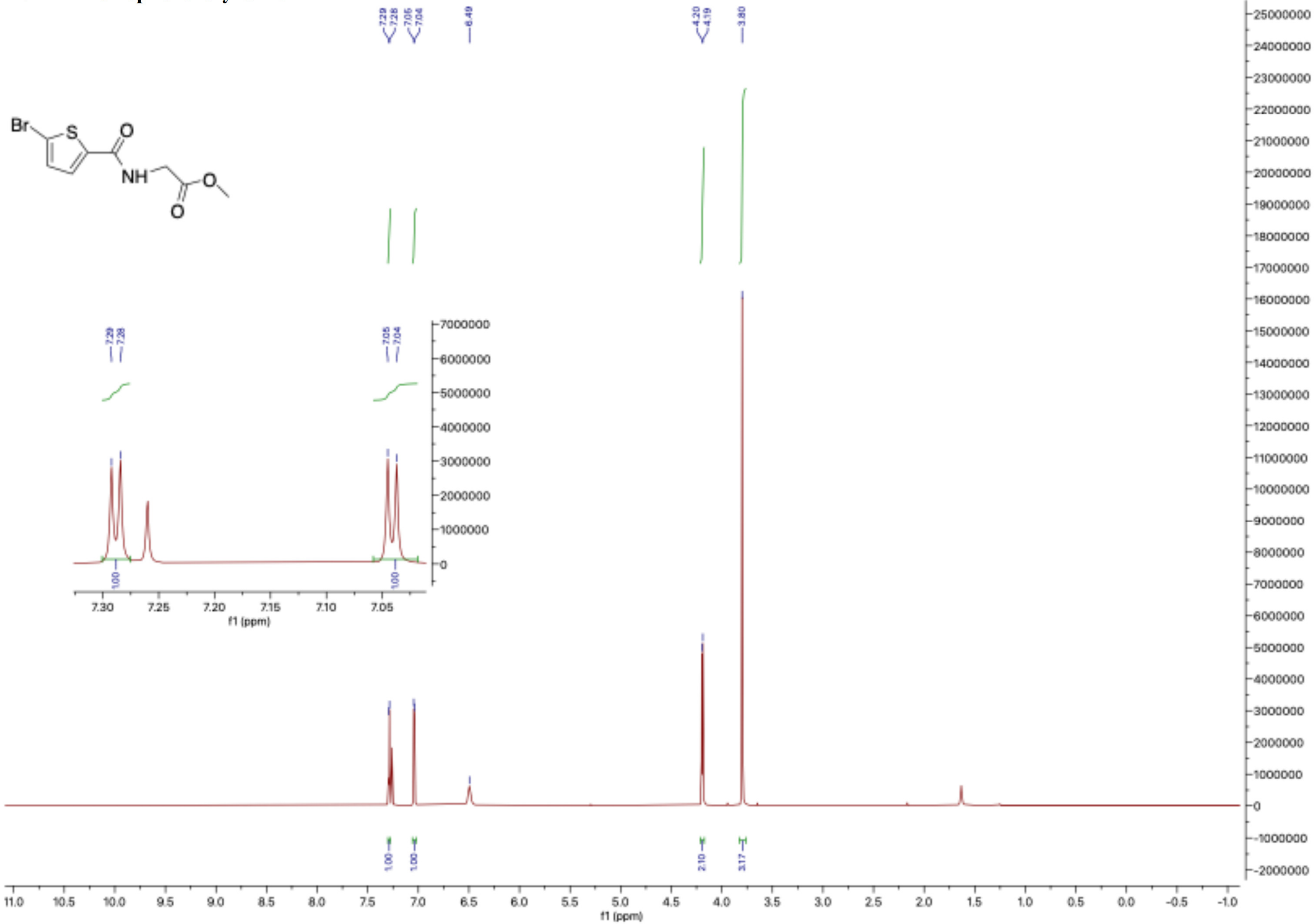

^{13}C NMR Br-thiophene-Gly-OMe

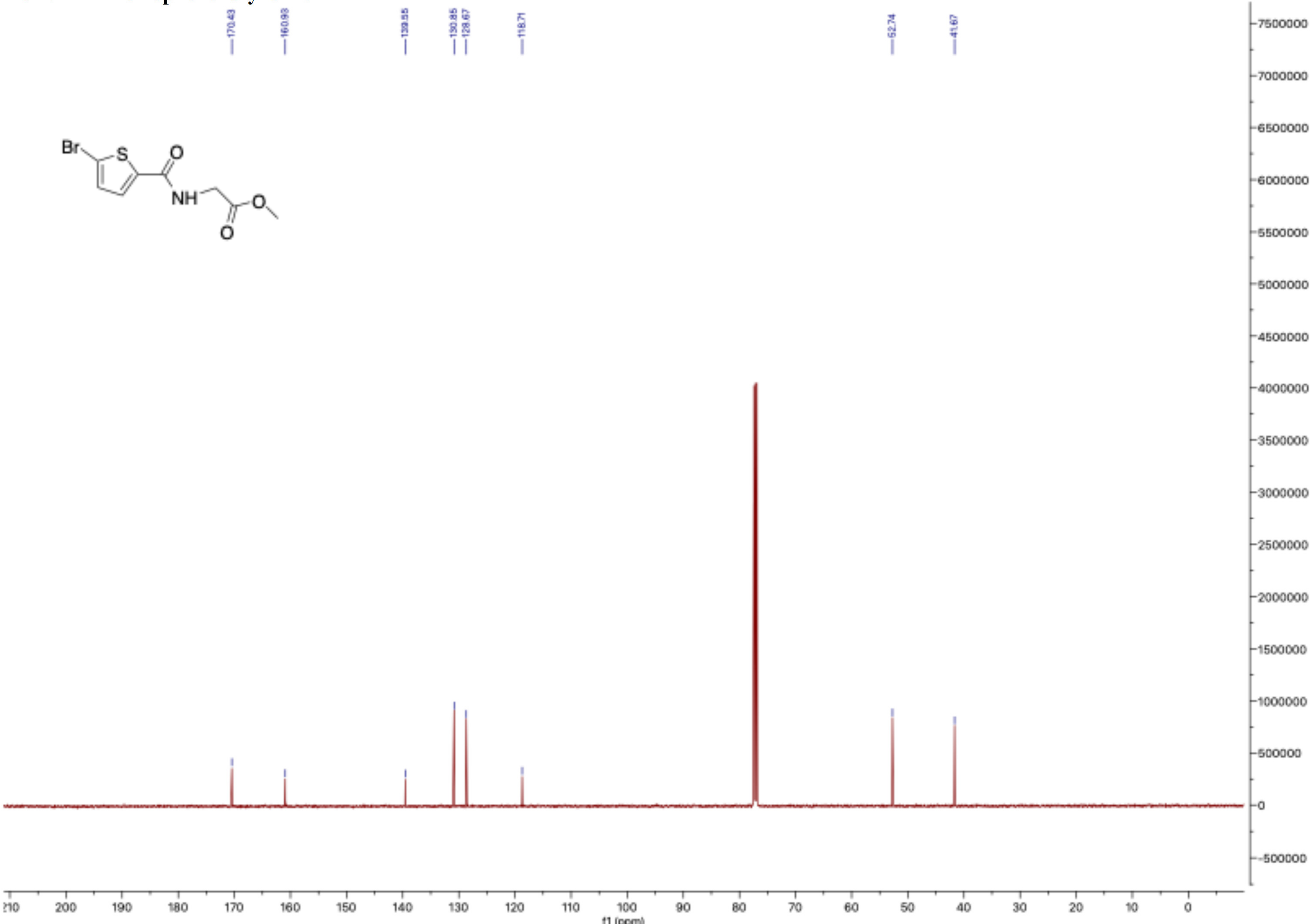

^{1}H NMR Formyl-bi-thiophene-Gly-OMe

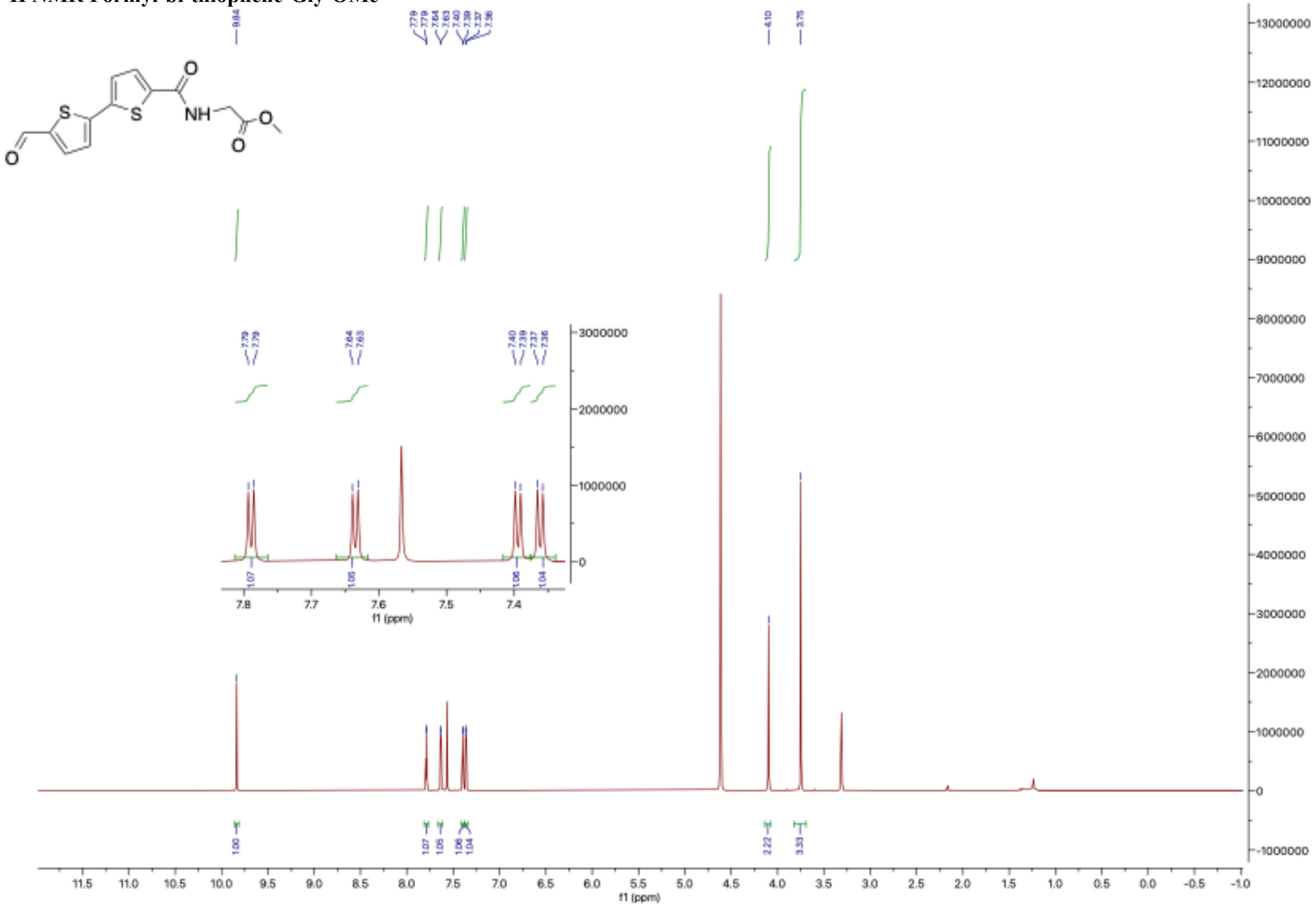

^{13}C NMR Formyl-bi-thiophene-Gly-OMe

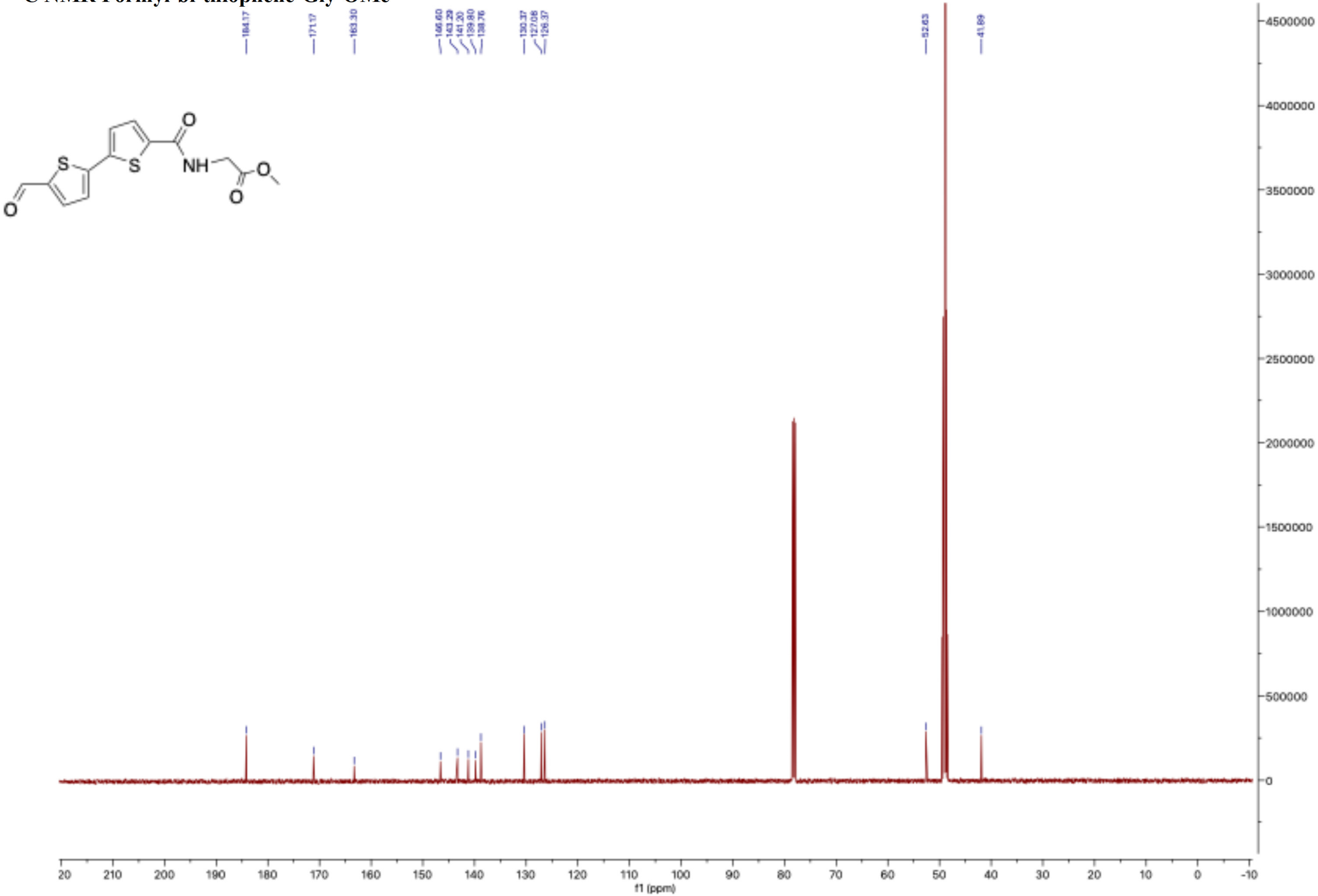

^{1}H NMR bTVBT-Gly-OMe

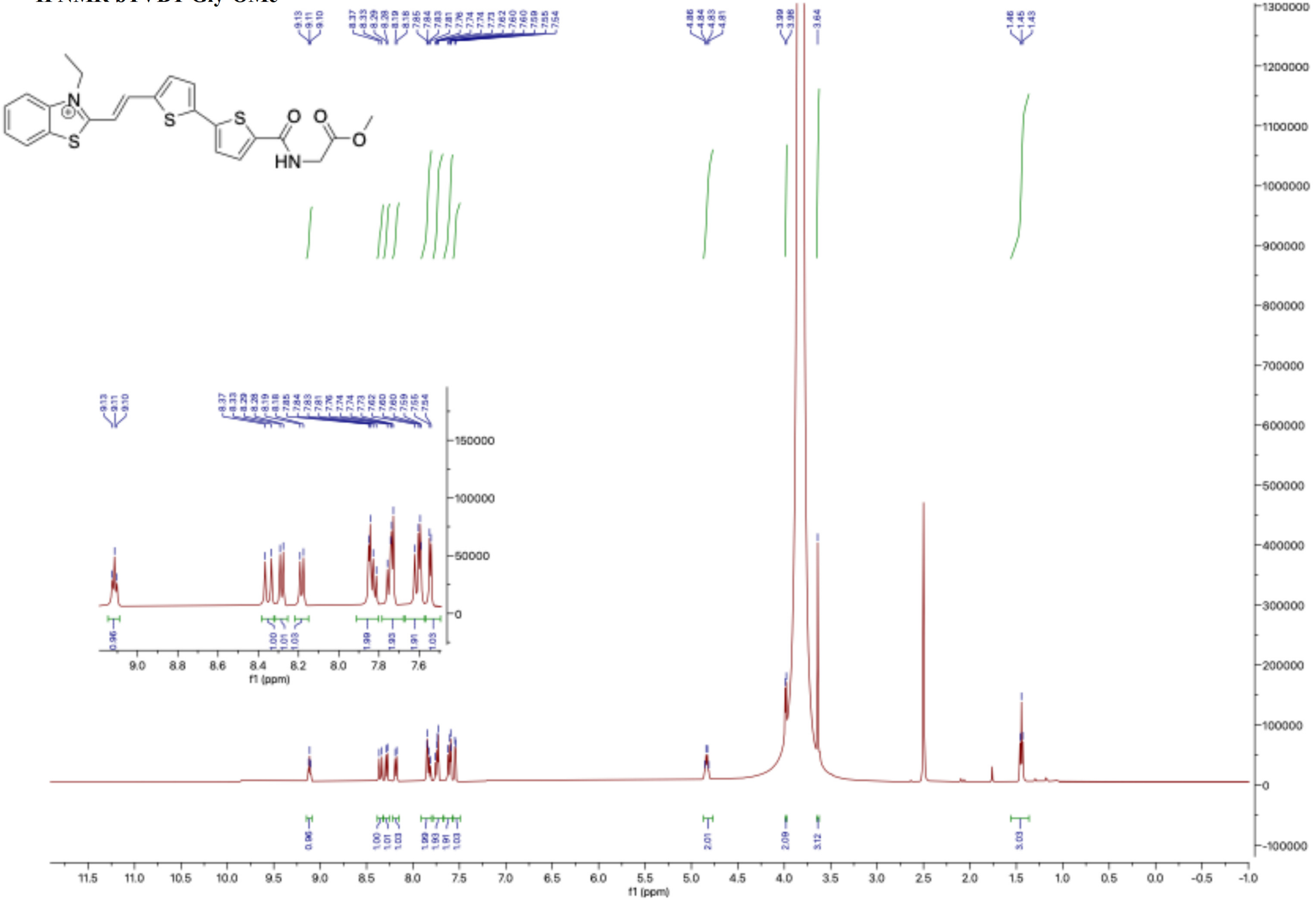

^{13}C NMR bTVBT-Gly-OMe

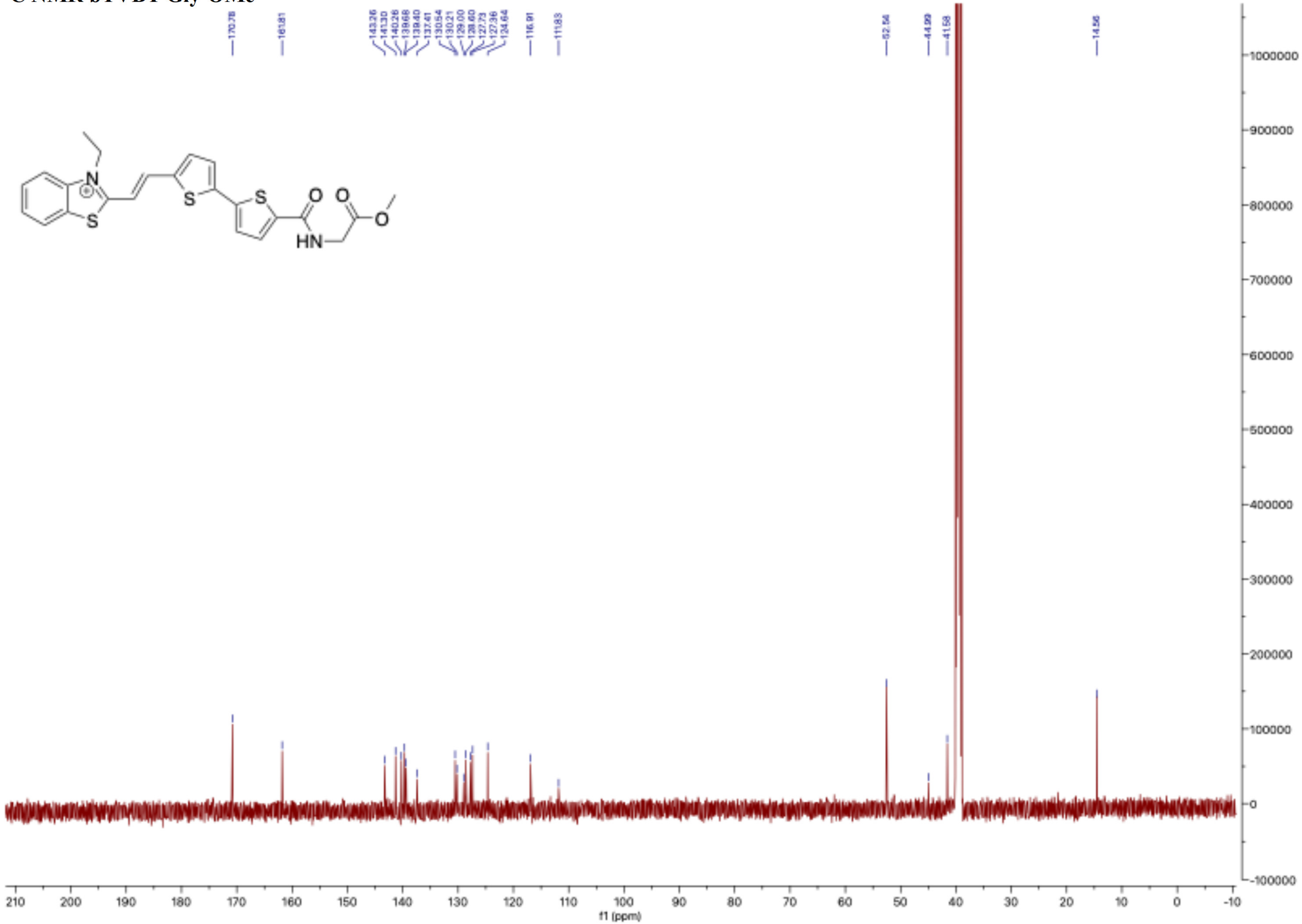

^{1}H NMR Br-thiophene-Ala-OMe

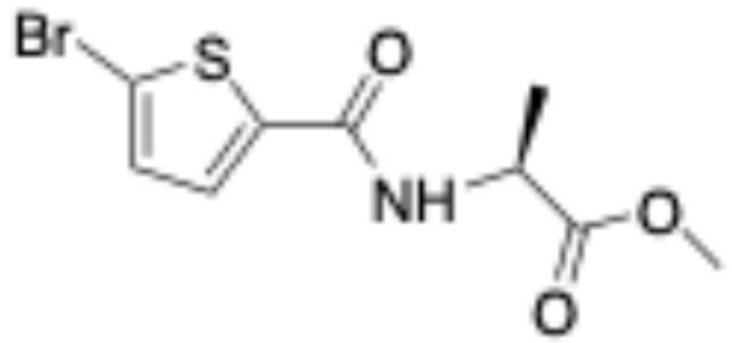

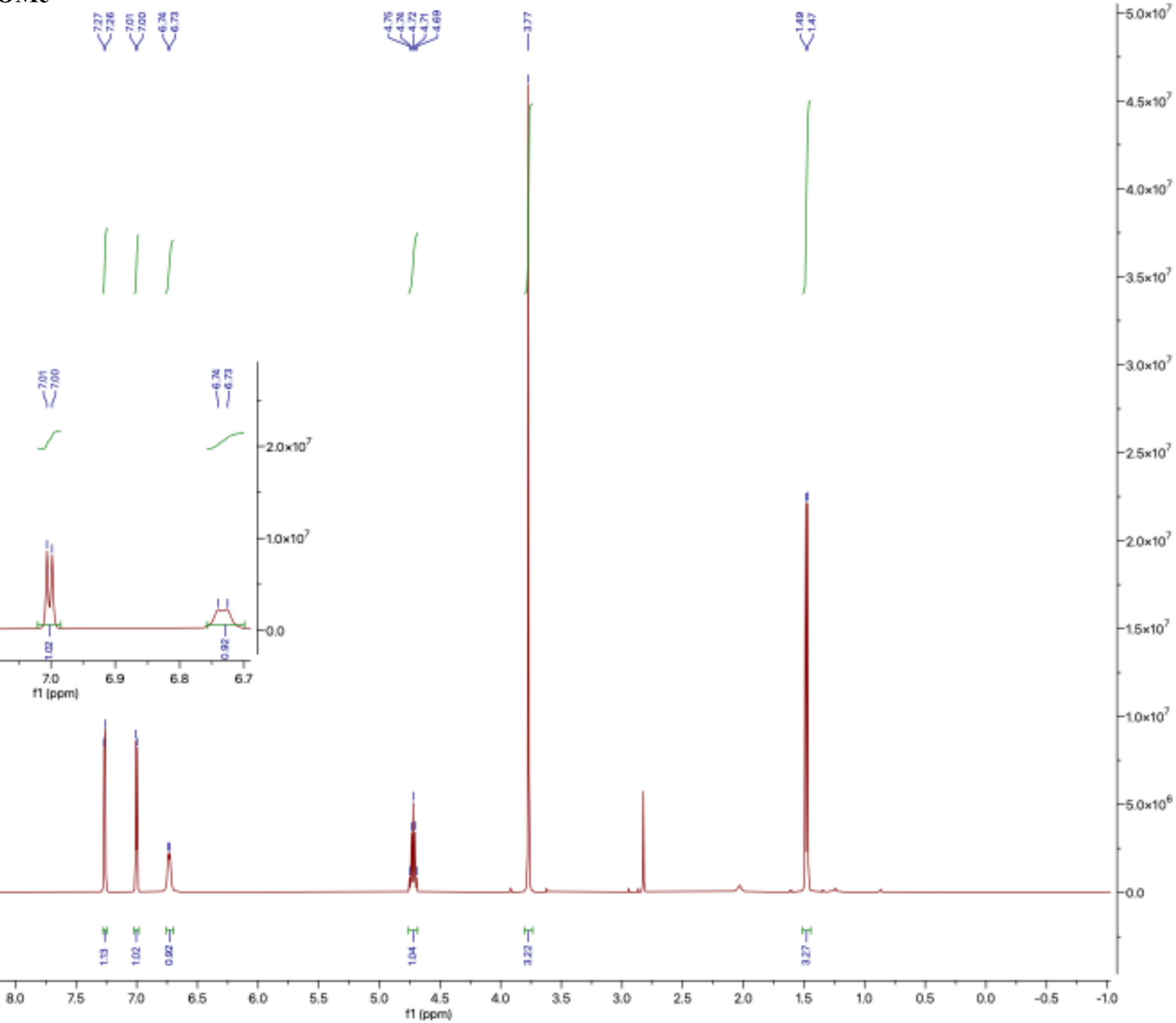

^{13}C NMR Br-thiophene-Ala-OMe

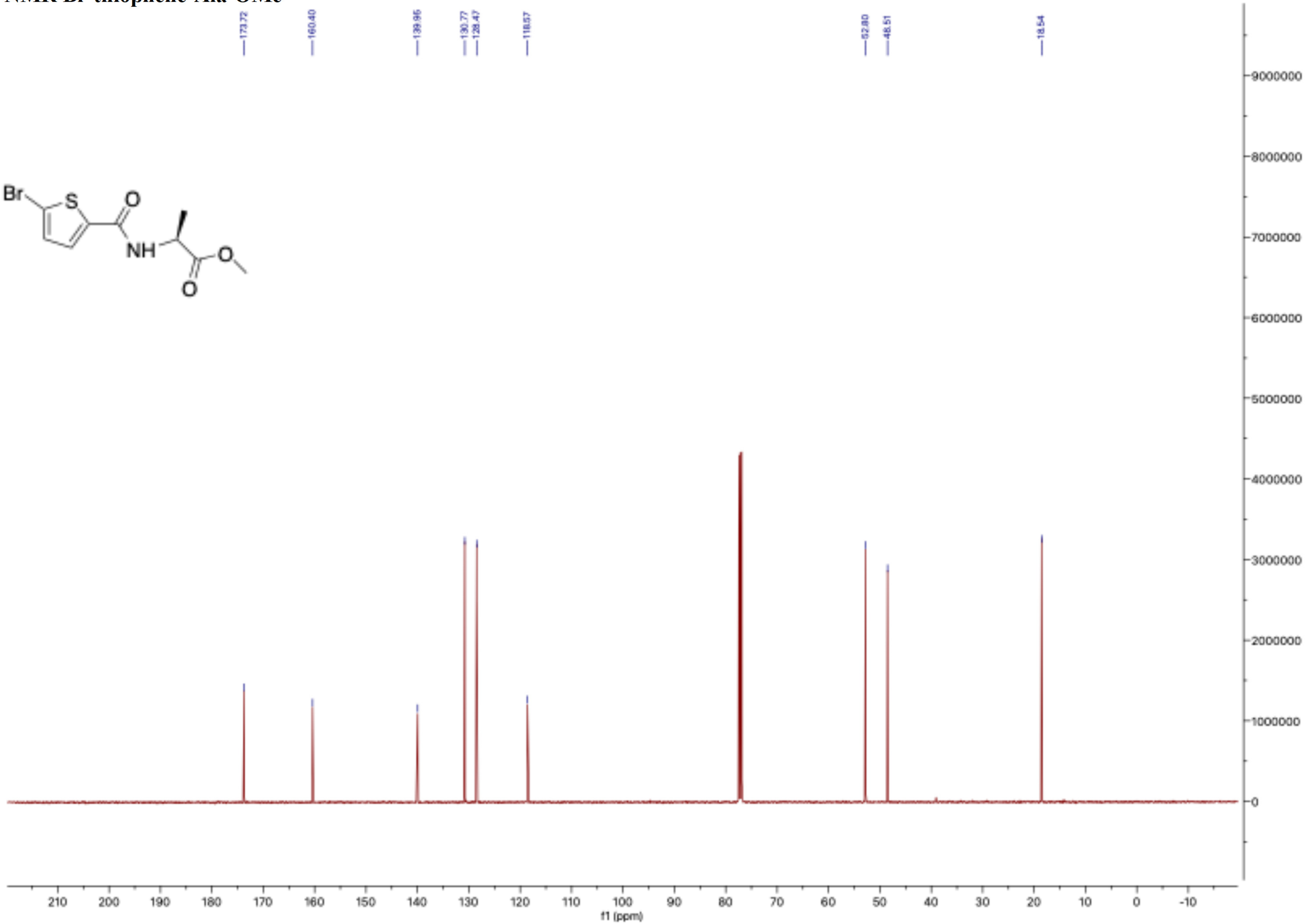

^{1}H NMR Formyl-bi-thiophene-Ala-OMe

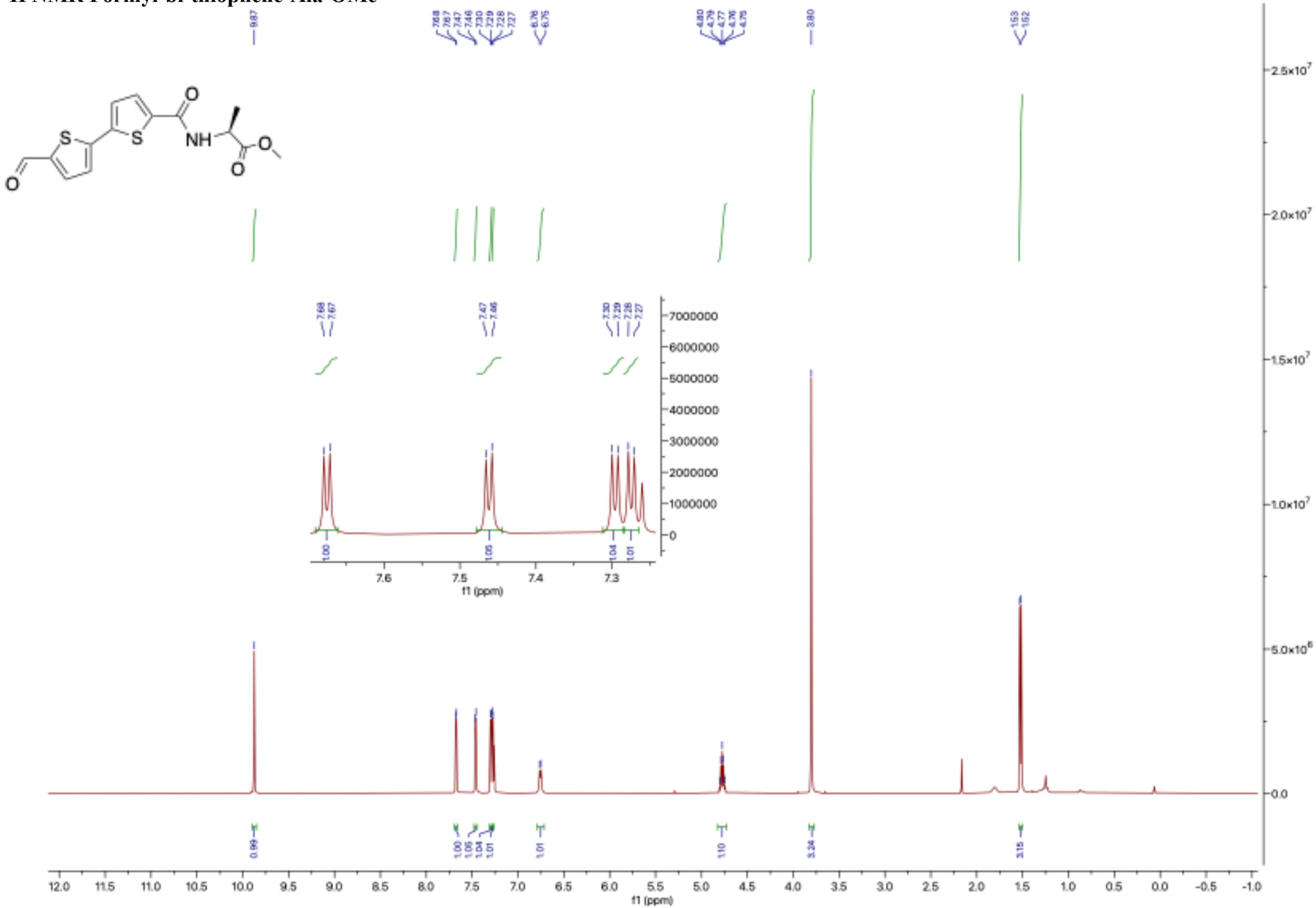

^{13}C NMR Formyl-bi-thiophene-Ala-OMe

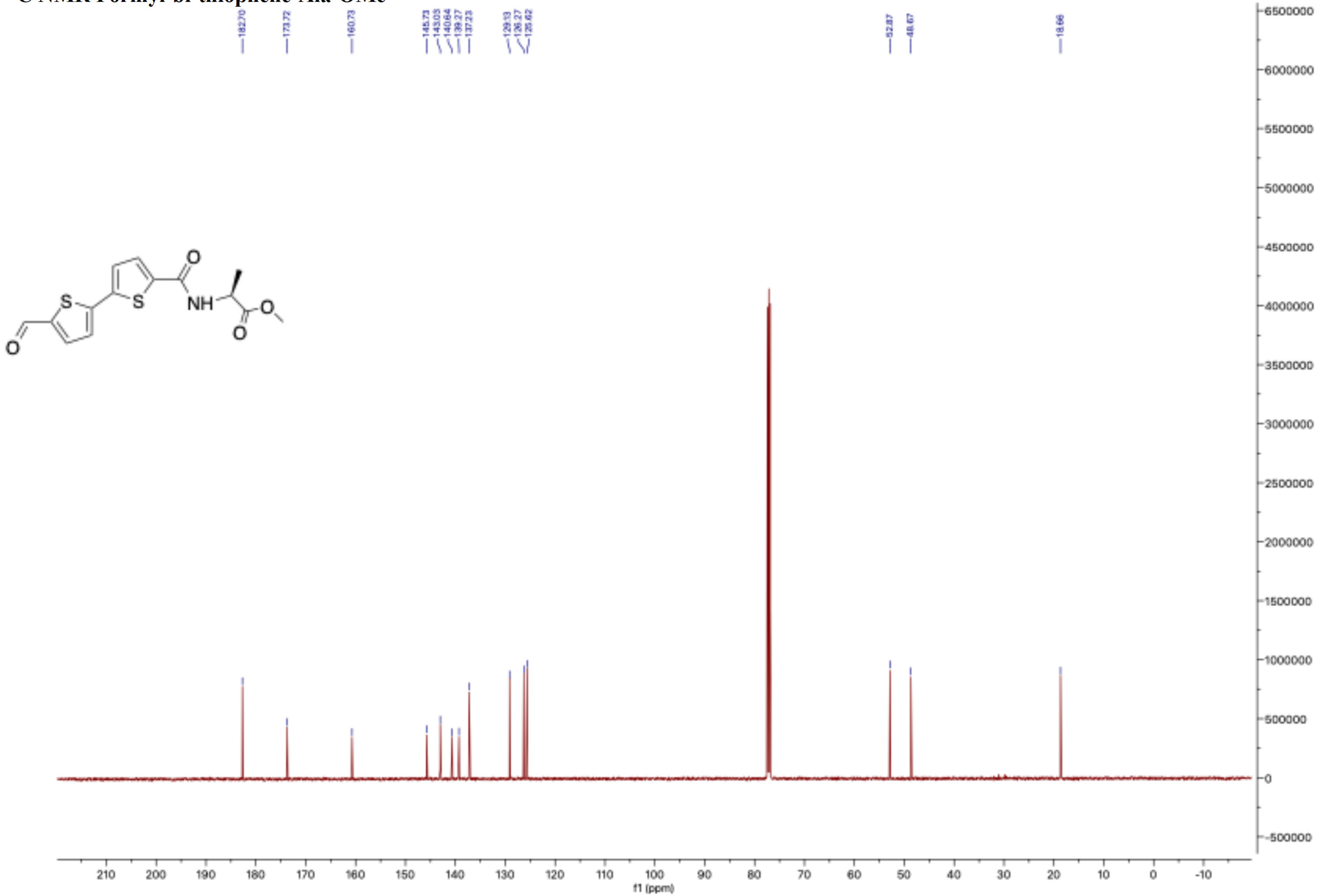

^{1}H NMR bTVBT-Ala-OMe

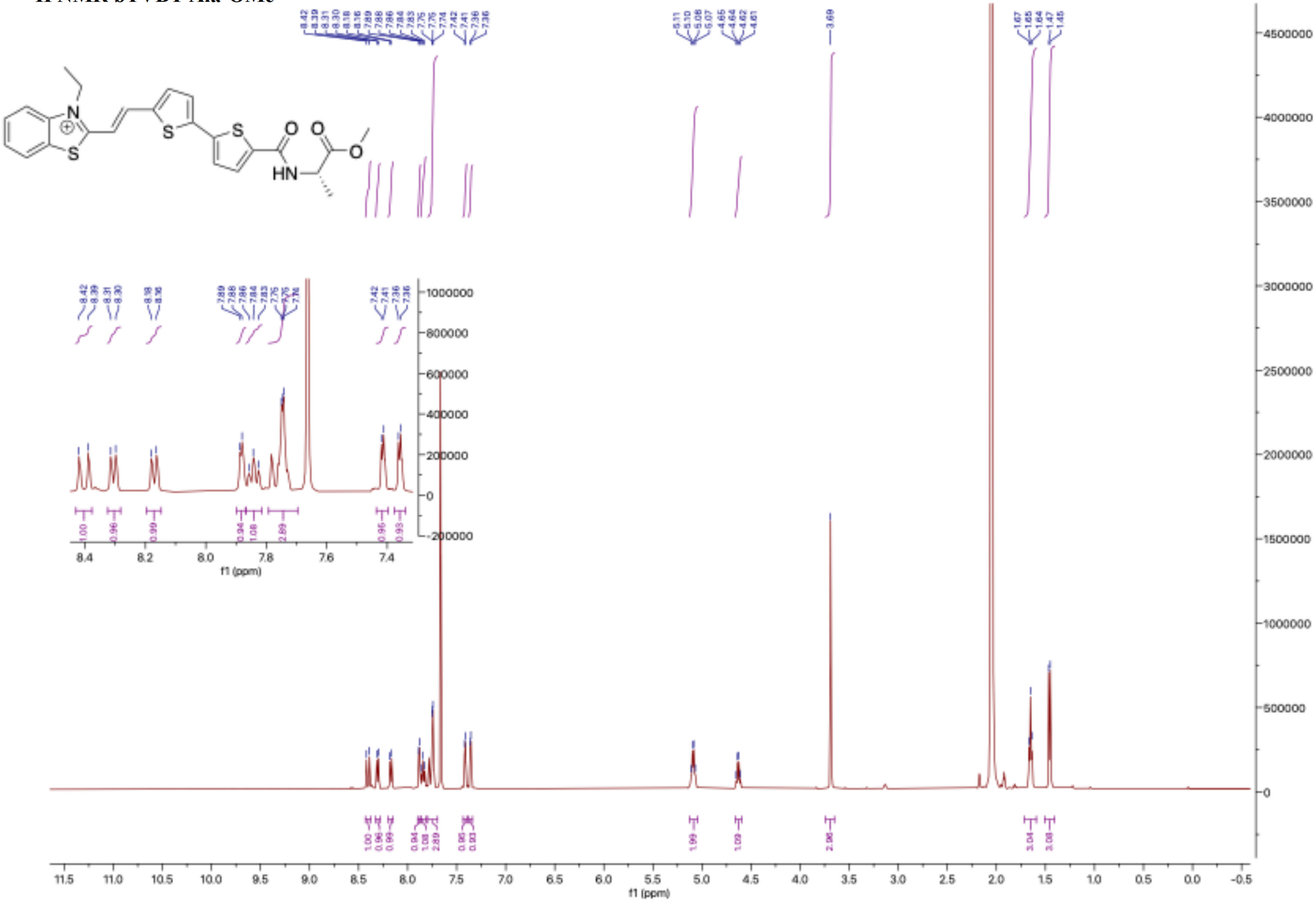

^{13}C NMR bTVBT-Ala-OMe

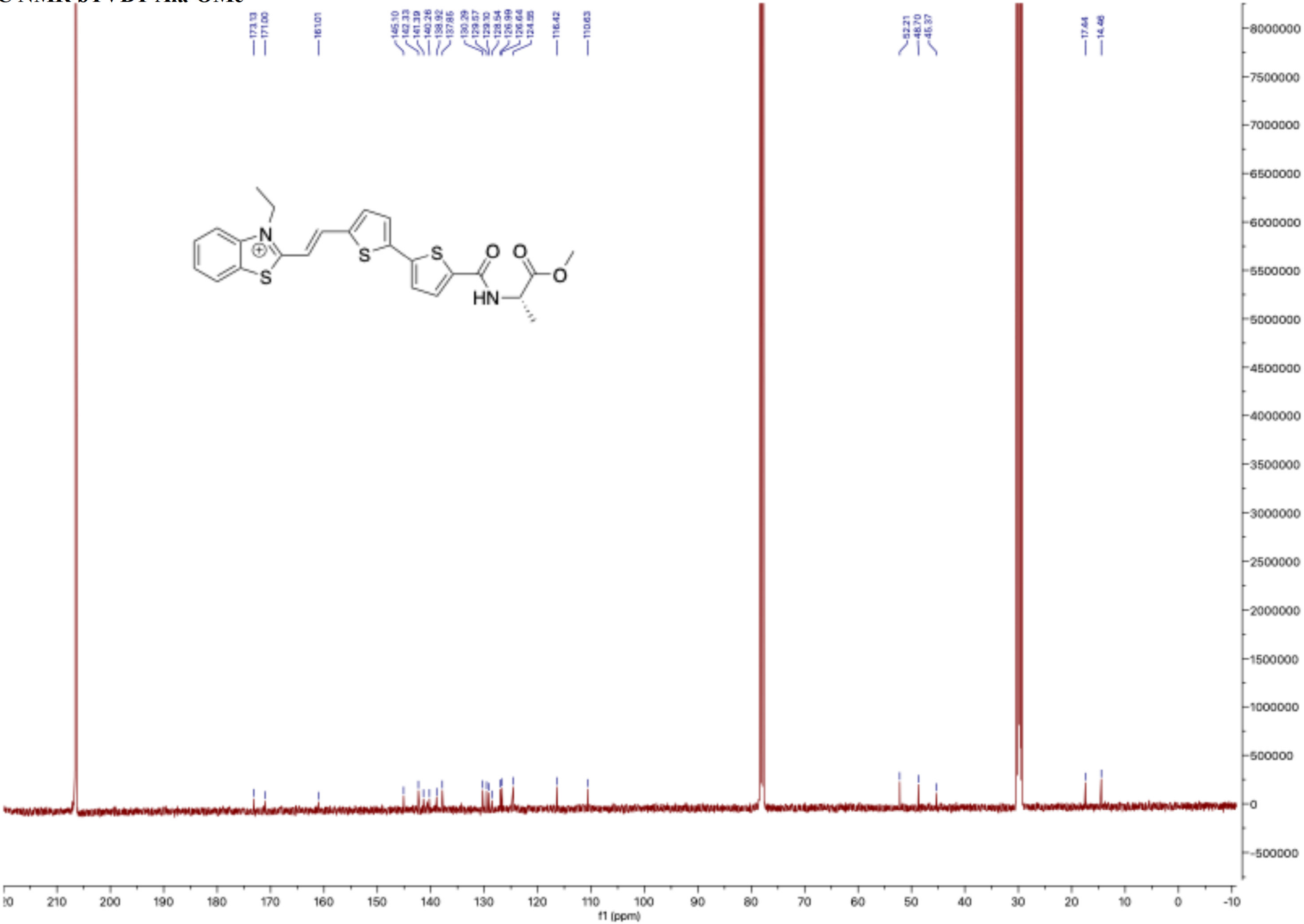

Supplement: Supplementary file 1 [file CMDC-21-e70558-s001.pdf]
